# Differences in global, regional, and national time trends in disability-adjusted life years for atrial fibrillation and flutter, 1990–2019: an age-period-cohort analysis from the 2019 global burden of disease study

**DOI:** 10.3389/fcvm.2024.1401722

**Published:** 2024-08-29

**Authors:** Juan Tang, Qingwei Zhang, Shengxian Peng, Huan Li, Weike Hu, Min Hao, Yue Liu, Mengyan Sun, Wenzhai Cao, Niying Yin, Xiaozhu Liu, Te Xu

**Affiliations:** ^1^ Department of Scientific Research, Zigong First People’s Hospital, Zigong, China; ^2^ Division of Gastroenterology and Hepatology, Key Laboratory Gastroenterology and Hepatology, Ministry of Health, State Key Laboratory for Oncogenes and Related Genes, Renji Hospital, School of Medicine, Shanghai Jiao Tong University, Shanghai Institute of Digestive Disease, Shanghai, China; ^3^ Chongqing College of Electronic Engineering, Chongqing, China; ^4^ Department of Continuing Education, Oxford University, Oxford, United Kingdom; ^5^ Department of Cardiology, Zigong First People’s Hospital, Zigong, China; ^6^ Department of Blood Transfusion, Suqian First Hospital, Suqian, China; ^7^ Department of Critical Care Medicine, Beijing Shijitan Hospital, Capital Medical University, Beijing, China; ^8^ Department of Cardiovascular Medicine, The Third Affiliated Hospital of Wenzhou Medical University, Wenzhou, China

**Keywords:** atrial fibrillation/flutter, age-period-cohort, global burden, disability-adjusted life years, atrial fibrillation/flutter

## Abstract

**Background:**

Atrial fibrillation and flutter, collectively referred to as AF/AFL, pose substantial public health challenges across nations of different economic statuses.

**Abjective:**

This research is intended to assess the discrepancies in global, regional, and national trends in DALYs for atrial fibrillation and flutter throughout 1990 and 2019.

**Methods:**

The GBD 2019 report included statistics on AF/AFL. An age-period-cohort (APC) model was used to calculate the changes in DALYs from ages 30 to 34 years up to 95 + years. The model calculated both net drifts and local drifts in DALYs. In addition, we analysed the relative risks for certain time periods and birth cohorts from 1990 to 2019 in order to assess their impact. In order to measure the changes over time in the age-standardized rate (ASR) of DALYs caused by AF/AFL, we calculated the average annual percentage changes (AAPCs) based on age, gender, socio-demographic index (SDI), and location. This approach enables us to analyse the impact of age, period, and cohort on trends in DALYs, which may uncover disparities in the management of AF/AFL.

**Results:**

The global number of DALYs cases was 8,393,635 [95% uncertainty interval (UI): 6,693,987 to 10,541,461], indicating a 121.6% rise (95% UI: 111.5 to 132.0) compared to 1990. From 1990 to 2019, the worldwide ASR of DALYs decreased by 2.61% (95% UI −6.9 to 1.3). However, the other SDI quintiles, except for high SDI and high-middle SDI, had an increase. During the last three decades, high-income nations in the Asia Pacific region had the most significant reduction in ASR of DALYs, whereas Central Asia experienced the highest rise (with a net drift of −0.9% [95% Confidence Interval (CI): −1.0 to −0.9] and 0.6% [95% CI: 0.5 to 0.7], respectively). Approximately 50% of the burden of AF/AFL has been transferred from areas with high and high-middle SDI to those with lower SDI. There was an inverse relationship between the AAPC and the SDI. In addition, men and older individuals were shown to have a greater burden of AF/AFL DALYs.

**Conclusion:**

The findings of this research demonstrate that the worldwide impact of AF/AFL remains significant and increasing, with the burden differing depending on SDI. The exhaustive and comparable estimates provided by these results may contribute to international efforts to attain equitable AF/AFL control.

## Introduction

Atrial fibrillation and flutter (AF/AFL) represent the most prevalent forms of persistent arrhythmia or tachycardia, negatively impacting patients’ quality of life and increasing their susceptibility to severe complications. They also stand as primary contributors to cardiovascular diseases, strokes, heart failure, and sudden death (1, 2). The global prevalence of AF/AFL is extensive and continues to rise (3, 4). For instance, the Asia-Pacific region has diagnosed over 16 million individuals with atrial fibrillation, with projections indicating this number will reach 72 million by 2050 (5). This surpasses the combined count of atrial fibrillation cases in Europe and the United States, more than doubling it (5). The main factors contributing to this trend are the rapid ageing of the population and a significant increase in lifestyle-related risk factors. Adverse events associated with AF/AFL incur substantial medical expenses and pose a significant public health burden. In the Asia-Pacific region, healthcare costs related to AF/AFL increase approximately 1.8–5.6 times every 10 years (6). AF/AFL attributed a staggering 287,241 deaths in 2017 (7), and by 2019, the global number of AF/AFL patients had reached 59.7 million, double the estimated cases reported in 1990 (8). Throughout the past few decades, AF/AFL has consistently maintained its position as one of the primary causes of death worldwide.

Moreover, some countries or regions experience the paradox of a significant increase in economic income coupled with low cultural and educational levels. This phenomenon results in an elevated incidence of AF/AFL, leading to increased mortality and a heavier disease burden. This situation is a direct consequence of globalization's impact on the economy (9). Since the Global Burden of Disease Study 2019 (GBD 2019), no systematic research has comprehensively updated the trends in DALYs related to AF/AFL. By converting the estimation of morbidity and mortality rates into a unified metric, the burden of disease assessments facilitate comparisons with other causes of disease and injury (10). We achieve this by standardising the impact of morbidity and mortality as a measure of population health loss over time, utilising the composite metric of DALYs (10). DALYs amalgamate the years of healthy life forfeited to disability and premature death, offering a uniform gauge of the total population health burden resulting from a particular condition. Researchers and policymakers can effectively assess and compare the burden of various health conditions using DALYs, which aids in the development of targeted interventions and resource allocation strategies. To inform the development of effective control and prevention strategies, it is critical to accurately assess the current burden of AF/AFL in terms of DALYs.

Hence, the aim of this research is to do a methodical and thorough examination of the illness burden linked to AF/AFL, using the most recent GBD 2019 estimations. The research attempts to distinguish the relative impacts of age, period, and cohort effects on DALYs using APC models. The study will include global, regional, and national scales, investigating the variations in AF/AFL DALYs across 204 nations and territories from 1990 to 2019, and evaluating the trends of their occurrence.

## Methods

### Data sources

The GBD 2019 database included the latest and most thorough estimates of descriptive epidemiological data for 369 diseases and injuries affecting 204 nations and territories. Raw data was retrieved from this database. The database includes information on incidence, prevalence, fatalities, and DALYs spanning the years 1990 through 2019. A collaborative effort involving researchers from multiple countries collected and compiled the data by sourcing information from published literature, surveys, and epidemiological sources (8). Previous publications have provided in-depth descriptions of the contents and methods employed by GBD 2019 (11–13). The University of Washington's Institutional Review Board approved the use of deidentified data in the GBD research and waived the requirement for informed permission.

### Atrial fibrillation/flutter definition

The identification of AF/AFL was conducted using the International Classification of Diseases and Injuries (ICD-9 and ICD-10). More precisely, the study categorised all cardiovascular disorders within the range of 427.3–427.32 in the ICD-9 and I48–I48.92 in the ICD-10 as AF/AFL. The diagnosis of AF/AFL was determined using electrocardiograms (ECGs), as has been done in previous worldwide investigations (13, 14).

Multiplying the number of deaths by the standard life expectancy at the time of death yielded the years of life lost (YLLs). The calculation of Years Lived with Disability (YLDs) involved incorporating the severity of the disability into the weights assigned to the number of YLLs with disability. Combined DALYs, the sum of YLDs and YLLs attributable to premature mortality in the population was utilised.

A SDI was allocated to each nation for the purpose of this research. The University of Washington's Institute for Health Metrics and Evaluation Research and the 2015 Global Burden of Disease Study came out with a new development categorization index they call the SDI (14). It has a strong correlation with a population's socioeconomic position and health outcomes. The index is a comprehensive metric that combines three factors: the lagged distribution of per capita income, the average education level of those aged 15 and above, and the total fertility rate among those under 25. The SDI, a comprehensive indicator of social population development, indicates a value of 0 when the lagged distribution has the lowest per capita income, the highest total fertility rate under 25, and the lowest years of schooling for those aged 15 and above. This indicates that, in theory, the area has the lowest level of development in relation to health outcomes. A SDI score of 1 indicates that the region has reached its maximum potential level of development in terms of health outcomes. When ranked from small to large SDI values, the index is divided into five equal points, and countries are then classified into low SDI, middle (including low-middle, middle, and high-middle) SDI, and high SDI based on their respective SDI values (14). The SDI can be utilised to monitor progress in crucial areas of socio-economic development, observe imbalances in development between and within countries, and assess the impact of health policies on health outcomes (15). If significant differences in SDI exist across various regions of a country, substantial variations are anticipated in disease burden indicators.

### Statistical analysis

This study examined DALYs trends using an APC model framework. The APC model was designed to investigate how age-related biological, technological, and social variables influence patterns of illness. This approach goes beyond traditional epidemiological analysis, aiming to understand the underlying causes that shape trends in DALYs (16). In essence, the APC model employs a log-linear Poisson model to analyse observed rates on a Lexis diagram, quantifying the combined effects of age, period, and birth cohorts. The Lexis diagram functions as a coordinate system that integrates age and calendar time, representing individual lives as line segments with a unit slope. The identification issue is one statistical challenge that arises when trying to assess the effects of cohort, period, and age separately (16). This is because cohort, period, and age are completely linearly related. To solve this problem, we define estimable APC functions and parameters without forcing the underlying model to conform to any particular set of rules.

The APC model accurately estimated the overall temporal trend in DALYs by providing yearly percentage change estimates (i.e., the net drift of DALYs, given as a percentage each year). The net drift consists of two components: the time-related trend and the cohort-related trend. In addition, the APC model calculated the temporal pattern of DALYs for each age group by determining the yearly percentage change of age-specific DALYs. This represents the local change in DALYs over time, expressed as a percentage per year, and reflects the impact of birth cohort effects. A significant alteration in DALYs is defined as a deviation of ±1% or more each year (17). This estimate corresponds to an approximate variation of ±10%, ±18%, and ±26% in the fitted rate during a period of 10, 20, and 30 years, respectively (17).

Furthermore, we used the Wald Chi-square test to determine the statistical significance of the trend in the yearly percentage change (17). In the reference cohort, the APC model produces results that include estimated longitudinal age-specific rates. We correct these rates for period deviations to account for age-related natural history, commonly referred to as age effects. Additionally, we calculate the relative risks of DALYs for each period (cohort) to illustrate the impacts specific to that period (cohort) (17). By comparing the rates for each period (cohort) by age to the rates for the reference period (cohort), we can determine the relative risk. Both rate ratio curves, which span distinct time periods (cohorts), reflect the full extent of the net drift. The choice of the referent period (cohort) does not impact the interpretation of results (11, 17).

We quantified the DALYs measures to assess the AF/AFL burden in the GBD 2019. We reported the percentage change in ASR with a 95% uncertainty interval (UI) using the estimated ASR per 100,000 people. The findings were categorised by global, gender, SDI quintile, and geographic region. We determined the ASR by directly adjusting for the worldwide distribution of ages. We generated 1,000 draw-level estimates for each parameter to acquire the UIs. We calculated the range between the 25th and 975th values over all 1,000 draws to get the 95% UI (14). A *p*-value less than 0.05 determined statistical significance, and all tests were two-tailed. We conducted all analyses using R (version 3.6.3).

### Ethics approval and consent to participate

The authors’ study was granted an exemption from ethical clearance by the medical ethics commission of the First People's Hospital of Zigong. This exemption was granted since the GBD public database was used, and all participant data in the database was made anonymous.

## Results

### 1990–2019 global and regional disease burden trends in AF/AFL DALYs

**Figure 1:**
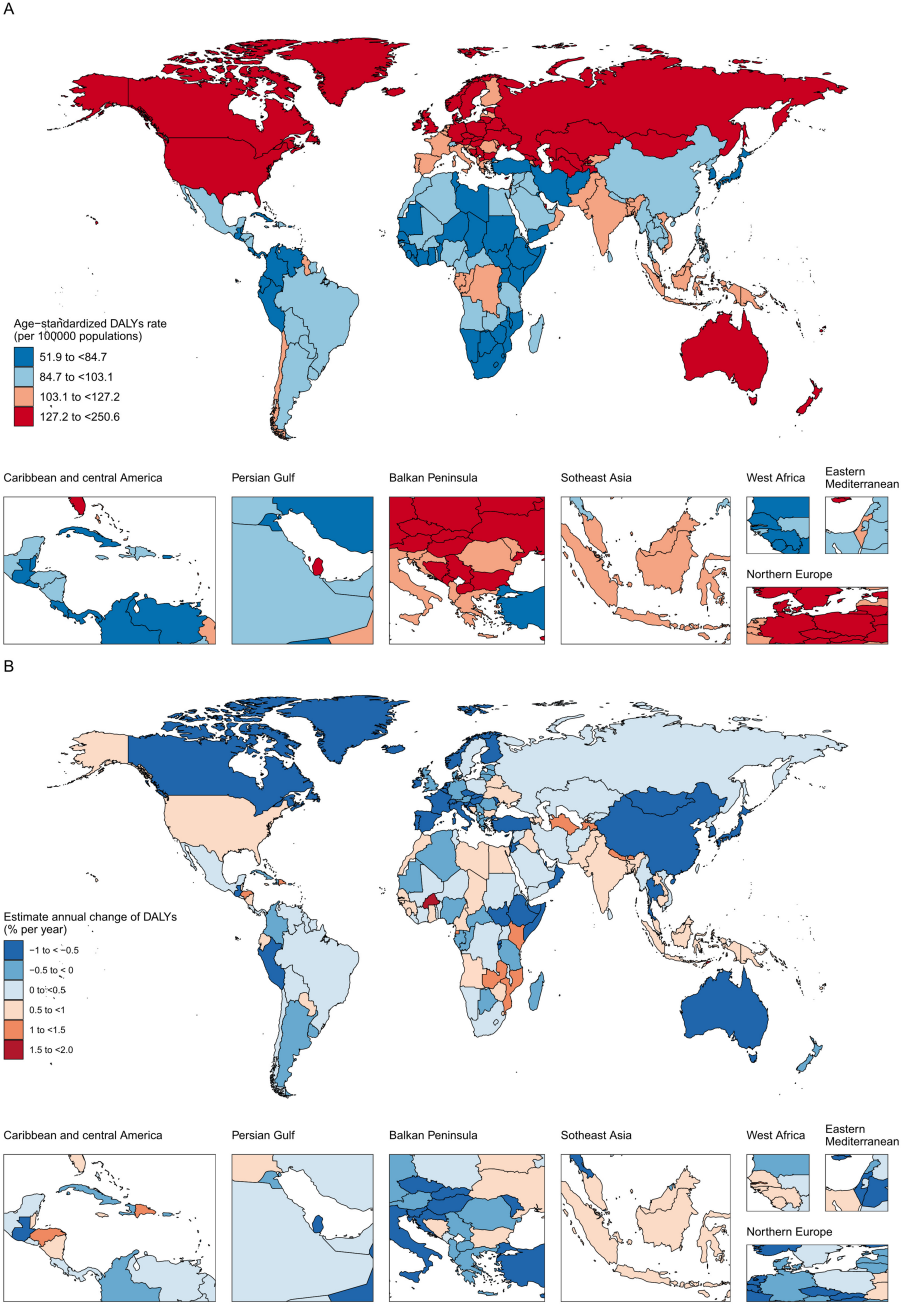
The age-standardized DALY in 2019 **(A)** and net drift of DALY during 1990–2019 **(B)** for atrial fibrillation and flutter in 204 countries and territories **(A)** world map of age-standardized DALY for atrial fibrillation and flutter in 2019, **(B)** world map of net drifts for atrial fibrillation and flutter DALY, ie, estimated annual percentage change of DALY from age-period-cohort model. Net drift captures components of the trends attributable to calendar time and successive birth cohorts.

Table 1, Figure 1, and Supplementary Table S1 present the overall count of DALYs cases, age-standardized DALYs, and the net drift of DALYs. The net drift is estimated from the APC model and represents the annual percentage change of DALYs, incorporating both the effects of calendar time and successive birth cohorts (17). From 1990 to 2019, there was a substantial rise in the global disease burden of AF/AFL (Table 1). DALYs cases increased from 3,787,838 [95% uncertainty interval (UI): 2,961,188 to 4,832,673] in 1990 to 8,393,635 [95% uncertainty interval (UI): 6,693,987 to 10,541,461] in 2019, marking an increase of 121.59%. All quintiles of SDI showed an increase between 1990 and 2019, with the exception of high and high-middle SDI, where the global DALYs ASR fell by 2.61% (95% UI −6.88 to 1.25). The greatest relative reduction in the age-standardized rate of DALYs occurred in high-income Asia Pacific [−19.3% (−24.9 to −14.4)] and Australasia [−8.4% (−12.7 to −3.1)], while the lowest reduction was observed in Andean Latin America [−1.4% (−17.2 to 18.4)]. Conversely, the largest relative increase in the age-standardized DALYs rate was observed in Central Asia [26.0% (11.8–39.7)] and high-income North America [13.9% (6.9–20.4)]. The percentage change in the age-standardized rate of DALYs cases also decreased in all SDI regions except for Middle SDI, Low-Middle SDI, and Low-SDI regions, where increases of 4.8% (−2.2 to 12.3), 10.4% (0.0–21.5), and 9.1% (−2.0 to 22.8) were observed, respectively. Significantly, over half of the burden of AF/AFL has transferred from areas with high and high-middle SDI to regions with lower SDI.

**Table 1:** Trends in atrial fibrillation and flutter DALY across socio-demographic index quintiles and 21 regions, 1990–2019.

Location	Case_1990	Case_2019	Case_percent_change	ASR_1990	ASR_2019	ASR_percent_change	AAPC	Netdrift
Global	3,787,838 (2,961,188, 4,832,673)	8,393,635 (6,693,984, 10,541,461)	121.59 (111.51, 131.96)	110 (87.66, 139.16)	107.13 (86.18, 133.73)	−2.61 (−6.88, 1.25)	−0.08 (−0.11, −0.06)	−0.04 (−0.06, −0.02)
High SDI	1,361,228 (1,075,403, 1,754,752)	2,517,229 (2,012,613, 3,144,013)	84.92 (74.37, 94.56)	128.62 (101.73, 165.21)	122.64 (97.3, 153.57)	−4.65 (−9.7, −0.56)	−0.11 (−0.17, −0.05)	0.04 (0.01, 0.08)
High-middle SDI	1,108,696 (863,012, 1,438,273)	2,214,107 (1,734,630, 2,823,347)	99.7 (90.71, 108.04)	116.99 (92.57, 149.81)	110.21 (86.73, 140.14)	−5.8 (−10.4, −2.46)	−0.22 (−0.26, −0.17)	−0.26 (−0.28, −0.23)
Middle SDI	745,665 (576,304, 952,661)	2,113,471 (1,653,609, 2,694,931)	183.43 (164.52, 202.78)	93.38 (74.4, 116.56)	97.9 (78.07, 122.72)	4.84 (−2.21, 12.28)	0.15 (0.11, 0.18)	0.16 (0.14, 0.19)
Low-middle SDI	421,268 (319,702, 540,873)	1,173,891 (929,505, 1,468,081)	178.66 (154.12, 208.38)	91.5 (71.71, 114.77)	101.01 (81.22, 123.92)	10.39 (0.04, 21.52)	0.37 (0.34, 0.41)	0.29 (0.26, 0.33)
Low SDI	149,125 (112,096, 190,132)	370,883 (291,504, 456,045)	148.71 (125.67, 178.17)	84.28 (63.03, 105.84)	91.91 (71.89, 111.57)	9.05 (−2.03, 22.82)	0.3 (0.27, 0.33)	0.2 (0.17, 0.23)
High-income Asia Pacific	124,789 (101,117, 156,715)	265,228 (212,780, 329,103)	112.54 (93.78, 131.87)	66.35 (54.42, 82.7)	53.53 (43.1, 66.58)	−19.32 (−24.87, −14.36)	−0.85 (−1.02, −0.69)	−0.94 (−1.03, −0.85)
High-income North America	514,635 (396,815, 674,657)	1,059,274 (837,806, 1,336,169)	105.83 (92.46, 117.79)	140.63 (108.35, 183.61)	160.18 (125.7, 202.67)	13.91 (6.94, 20.42)	0.51 (0.36, 0.65)	0.82 (0.77, 0.88)
Western Europe	860,796 (682,643, 1,101,763)	1,347,220 (1,087,687, 1,676,380)	56.51 (43.58, 67.42)	144.62 (114.54, 185.07)	132.81 (105.93, 168.11)	−8.17 (−15.88, −3.09)	−0.23 (−0.37, −0.08)	−0.27 (−0.35, −0.18)
Australasia	42,220 (33,196, 53,571)	89,702 (71,277, 113,671)	112.46 (101.43, 126.39)	183.72 (145.83, 231.54)	168.28 (132.54, 214.32)	−8.4 (−12.73, −3.1)	−0.3 (−0.36, −0.25)	−0.31 (−0.39, −0.23)
Andean Latin America	11,255 (9,596, 13,090)	33,839 (28,083, 40,058)	200.65 (151.71, 262)	64.49 (55.4, 74.42)	63.61 (52.67, 75.22)	−1.37 (−17.21, 18.37)	0.04 (−0.27, 0.34)	−0.03 (−0.12, 0.07)
Tropical Latin America	73,160 (60,354, 90,768)	235,075 (193,674, 285,763)	221.32 (191.27, 238.41)	98.38 (81.69, 120.63)	102.34 (84.28, 124.34)	4.03 (−5.8, 8.7)	0.26 (0.07, 0.45)	0.49 (0.42, 0.57)
Central Latin America	56,099 (47,310, 68,851)	185,647 (152,660, 228,151)	230.92 (201.51, 260.23)	80.65 (68.44, 98.18)	82.84 (68.32, 101.95)	2.72 (−6.2, 11.21)	0.09 (−0.01, 0.19)	0.03 (−0.03, 0.09)
Southern Latin America	40,566 (33,439, 50,252)	86,620 (71,626, 107,603)	113.53 (100.39, 129.23)	96.08 (79.7, 117.41)	100.95 (83.35, 125.77)	5.07 (−1.35, 12.28)	0.2 (0.12, 0.28)	0.1 (0.03, 0.18)
Caribbean	18,756 (15,784, 22,495)	43,197 (36,148, 51,752)	130.31 (108.67, 154.53)	79.4 (66.52, 94.7)	83.27 (69.65, 99.83)	4.88 (−4.74, 15.72)	0.18 (0.09, 0.28)	0.18 (0.09, 0.26)
Central Europe	188,357 (145,003, 244,839)	302,864 (237,301, 385,693)	60.79 (50.19, 71.63)	134.74 (105.22, 173.65)	136.04 (106.47, 173.32)	0.97 (−5.35, 6.88)	0.05 (0.02, 0.09)	0.03 (−0.01, 0.08)
Eastern Europe	316,576 (238,789, 415,981)	481,406 (368,863, 621,279)	52.07 (43.7, 60.86)	120.85 (92.65, 157.62)	136.59 (104.82, 177.07)	13.03 (6.79, 19.12)	0.43 (0.28, 0.59)	0.4 (0.36, 0.44)
Central Asia	46,069 (34,294, 60,476)	81,519 (63,510, 104,678)	76.95 (59.5, 92.05)	109.82 (82.86, 143.91)	138.35 (109.64, 176.31)	25.97 (11.82, 39.73)	0.82 (0.61, 1.02)	0.58 (0.48, 0.69)
North Africa and Middle East	106,968 (84,330, 135,033)	289,998 (229,100, 361,786)	171.11 (148.36, 207.34)	79.91 (63.99, 99.66)	81.61 (65.1, 100.78)	2.12 (−6.72, 17.86)	0.01 (−0.01, 0.04)	−0.01 (−0.07, 0.05)
South Asia	407,485 (301,692, 533,787)	1,257,580 (971,301, 1,609,102)	208.62 (173.69, 248.48)	96.83 (73.96, 124.55)	106.05 (82.89, 132.86)	9.52 (−3.92, 24.3)	0.32 (0.15, 0.5)	0.28 (0.23, 0.33)
Southeast Asia	195,204 (146,920, 256,580)	544,420 (417,735, 695,212)	178.9 (159.19, 201.73)	95.11 (73.3, 122.94)	105.7 (82.22, 133.52)	11.14 (2.64, 20.74)	0.37 (0.34, 0.39)	0.34 (0.32, 0.37)
East Asia	654,562 (493,067, 852,620)	1,793,188 (1,356,400, 2,304,890)	173.95 (148.95, 198.48)	98.28 (76.84, 124.34)	96.78 (75.08, 122.56)	−1.52 (−11.31, 7.75)	−0.08 (−0.15, −0.02)	−0.04 (−0.08, 0)
Oceania	2,539 (1,907, 3,307)	6,579 (5,066, 8,433)	159.14 (131.64, 190.33)	109.88 (83.38, 140.9)	116.58 (91.06, 148.01)	6.09 (−5.35, 18.08)	0.2 (0.17, 0.22)	0.24 (0.07, 0.42)
Western Sub-Saharan Africa	53,962 (41,706, 68,541)	118,338 (94,717, 145,563)	119.3 (80.73, 147.37)	82.99 (65.49, 103.45)	86.19 (70, 103.25)	3.86 (−16.3, 18.27)	0.12 (0.08, 0.16)	0.07 (0.02, 0.12)
Eastern Sub-Saharan Africa	40,780 (28,065, 51,395)	94,303 (66,626, 115,072)	131.25 (94.07, 179.12)	73.06 (48.45, 92.35)	77.09 (53.33, 94.33)	5.51 (−11.41, 26.89)	0.18 (0.09, 0.27)	−0.01 (−0.06, 0.04)
Central Sub-Saharan Africa	16,297 (10,915, 24,704)	40,758 (28,803, 53,877)	150.09 (100.07, 203.36)	99.09 (65.94, 148.49)	104.25 (72.95, 138.1)	5.2 (−14.35, 28.28)	0.16 (0.11, 0.21)	0.05 (−0.03, 0.14)
Southern Sub-Saharan Africa	16,762 (13,518, 20,930)	36,881 (30,132, 45,192)	120.02 (106.49, 134.44)	71.59 (58.23, 88.42)	79.24 (65.78, 95.69)	10.69 (3.17, 18.48)	0.38 (0.27, 0.5)	0.02 (−0.05, 0.09)

Age-standardised DALY rate is computed by direct standardisation with global standard population in GBD 2019. Net drifts are estimates derived from the age-period-cohort model and denotes overall annual percentage change in DALY, which captures the contribution of the effects from calendar time and successive birth cohorts.

SDI, socio-demographic index; APC, age-period-cohort; AAPCs, average annual percentage changes.

For each regions with an SDI level, Table 1 shows the number of DALYs, the percentage change in DALYs, the ASR, the percentage change in ASR, the AAPC, and the net drift of DALYs calculated using the APC model. Furthermore, Supplementary Table S1 presents comparable data, with a special emphasis on the uniformity of epidemiological characteristics and the proximity of geographical locations. Supplementary Table S2 presents the results of tests conducted for atrial fibrillation and flutter DALYs for both sexes, spanning the SDI quintiles and 21 regions from 1990 to 2019.

### Distribution of DALY trends in AF/AFL disease burden across regions and countries, 1990–2019

Out of the 204 countries and territories, 150 had at least 1,000 cases of AF/AFL DALYs in 2019. China had the highest number of DALYs with 1.7 million [(95% UI 1.3 to 2.2 million)], followed by India with 1.0 million [(0.8–1.3 million)], the United States of America with 1.0 million [(0.8–1.2 million)], Germany with 0.3 million [(0.3–0.4 million)], and the Russian Federation with 0.3 million [(0.2–0.4 million)]. These top five countries accounted for 51.9% of the global cases of AF/AFL DALYs. Moreover, our analysis revealed that Montenegro had the highest age-standardized DALYs worldwide for AF/AFL, with a rate of 250.6 (95% UI: 205.8–313.7) per 100,000 individuals. Conversely, Singapore had the lowest age-standardized DALYs, with a rate of 51.94 (95% UI: 40.0–67.0) per 100,000 people. Out of the 150 countries, 134 exhibited either an increasing trend (net drifts ≥0.0% per year) or a modest reduction (−0.5 to 0.0%) in DALYs cases. Uzbekistan experienced the largest rise in the ASR, going from 93.7 [65.6–131.7] to 164.0 [134.7–200.8], with a net drift of DALYs at 1.67% (1.6 to 1.8) per year. This was followed by Bahrain and Kenya, with a net drift of DALYs at 1.9% (0.9–2.9) per year and 1.1% (1.0–1.3) per year, respectively. High-middle SDI, high-income Asia Pacific, Western Europe, and Australasia all saw a reduction in DALYs in 2019, but high-income North America saw an unexpected increase, with a relatively flat net drift in DALYs of 0.8% (0.8 to 0.9) per year. In addition, the net drift of DALYs has been falling in some countries, with the most substantial decreases occurring in Bermuda (−1.0%; −3.3 to 1.3) and Burundi (−1.0%; −1.3 to −0.7) and Rwanda (−0.9%; −1.2 to −0.7). Due to their large populations, China and India recorded the highest number of DALYs cases. The ASR in China decreased by 1.6% (−11.6 to 8.1), while in India, it increased by 8.5% (−6.0 to 23.4). Both countries exhibited relatively modest net drifts of DALYs, with China experiencing a slight decrease of −0.04% (−0.01 to 0), and India observing a small increase of 0.3% (0.2–0.3). Taken together, these findings indicate that the trends in DALYs for AF/AFL varied across countries, and the gains in DALYs did not always align with expectations based on the national-level SDI status. This was evident in countries such as Bahrain and Burundi.

### Time trends in distribution of DALYs in AF/AFL disease burden across different age groups

**Figure 2:**
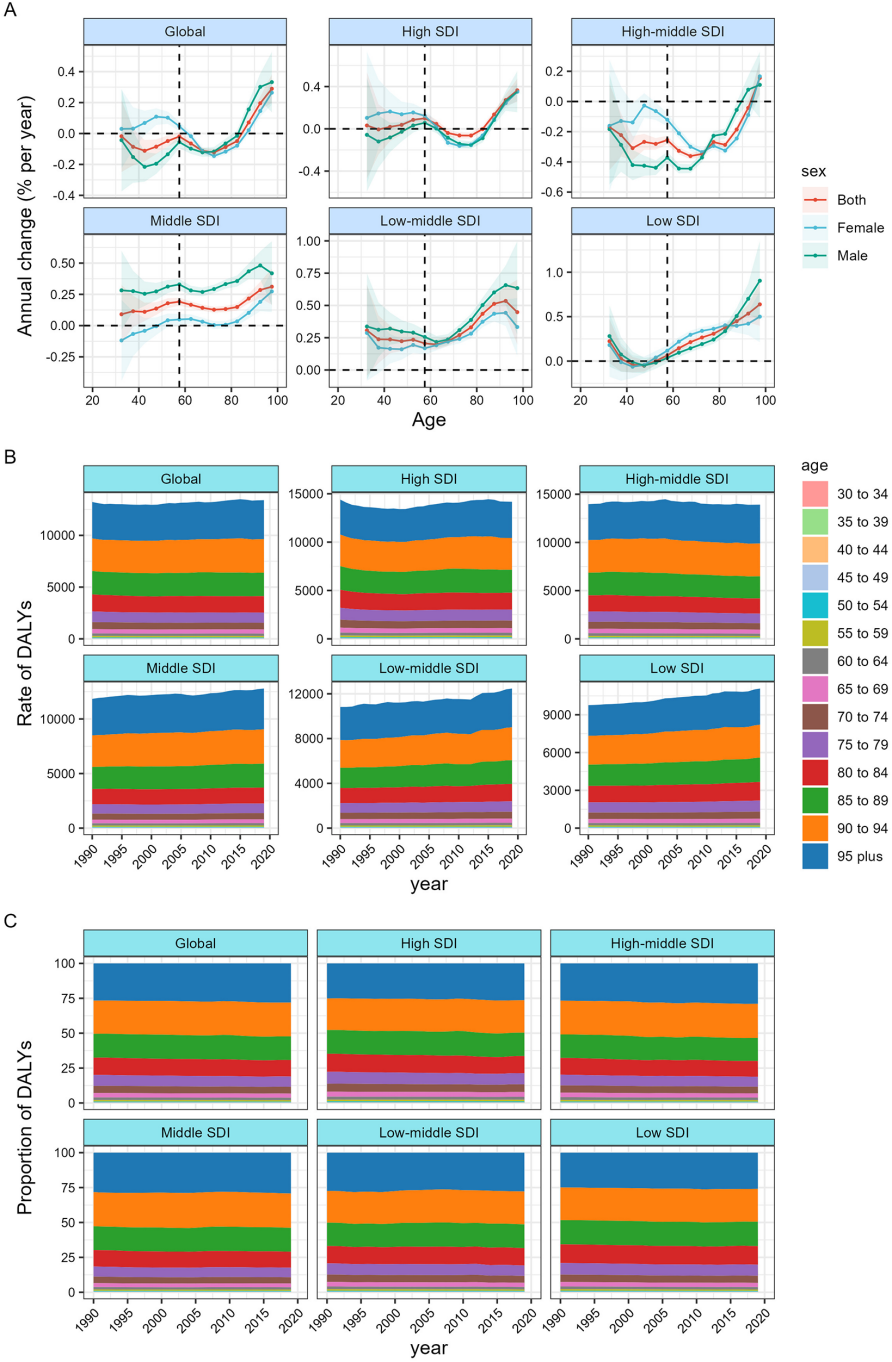
Local drifts of atrial fibrillation and flutter DALY and age distribution of atrial fibrillation and flutter DALY by SDI quintiles, 1990–2019. **(A)** Local drifts of atrial fibrillation and flutter DALY (estimates from age-period-cohort models) for 19 age groups (5–9 to 95 plus years), 1990–2019. The dots and shaded areas indicate the annual percentage change of DALY (% per year) and the corresponding 95% CIs. **(B)** DALY rates of Atrial fibrillation and flutter across age groups,1990–2019. **(C)** Temporal change in the relative proportion of atrial fibrillation and flutter DALY across age groups, 1990–2019. SDI = Socio-demographic Index.

Figure 2A illustrates the annual percentage fluctuation in the rate of AF/AFL DALYs for different age groups, ranging from 30 to 34 years old to 95 years and above. This data represents the local drift of DALYs computed using the APC model and encompasses the trends associated with birth cohort effects. Globally, AF/AFL DALYs showed decreasing trends in the under-60 age groups (*p* < 0.001), and this trend diminished as people became older. Additionally, in the high-middle SDI area, AF/AFL DALYs in almost all age groups decreased to some extent. However, other SDI areas exhibited varying degrees of an upward trend. It is worth noting that in almost all SDI regions, there is a significant upward trend in AF/AFL DALYs for those over 80 years of age. In the middle SDI, the annual percentage change in AF/AFL DALYs was greater for men than for women. In the high-middle SDI region, the AF/AFL DALYs of men under 65 years of age, without overlapping at 95% confidence intervals, are significantly slower than those of women. The local drift of AF/AFL DALYs for each country is shown in Supplementary Figures S1–S6.

Figures 2B,C demonstrate the chronological variations in the age distribution of AF/AFL DALYs. Globally, the disease burden, as measured in DALYs, is transitioning from impacting populations under 80 years old to those over 80 years old. This trend is particularly noticeable in nations with a middle-level SDI, a high-middle SDI, or a high SDI. The majority of DALYs in all nations with a SDI were predominantly concentrated in the age group of 50 and above in the year 2019. The age distribution of AF/AFL DALYs for each nation may be seen in Supplementary Figures S7–S18.

### Age, period, and cohort effects on AF/AFL DALYs

**Figure 3:**
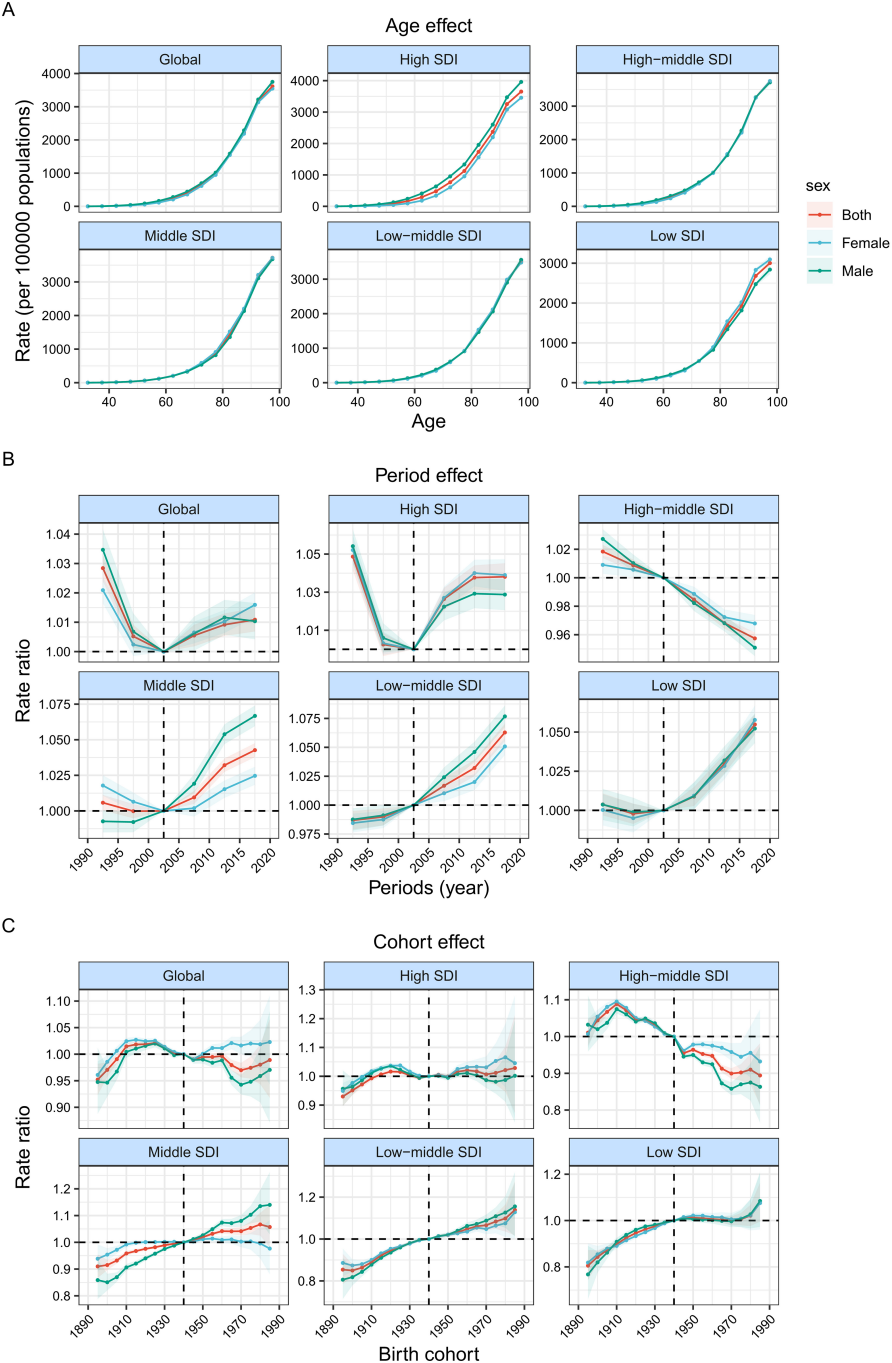
Age, period and cohort effects on atrial fibrillation and flutter DALY by SDI quintiles. **(A)** Age effects are shown by the fitted longitudinal age curves of DALY (per 100,000 person-years) adjusted for period deviations. **(B)** Period effects are shown by the relative risk of DALY (DALY rate ratio) and computed as the ratio of age-specific rates from 1990 to 1994 to 2015–2019 (2000–2005 as the referent period). **(C)** Cohort effects are shown by the relative risk of DALY and computed as the ratio of age-specific rates from the 1895 cohort to the 1985 cohort, with the referent cohort set at 1940. The dots and shaded areas denote DALY rates or rate ratios and their corresponding 95% CIs. SDI = Socio-demographic Index.

**Figure 4:**
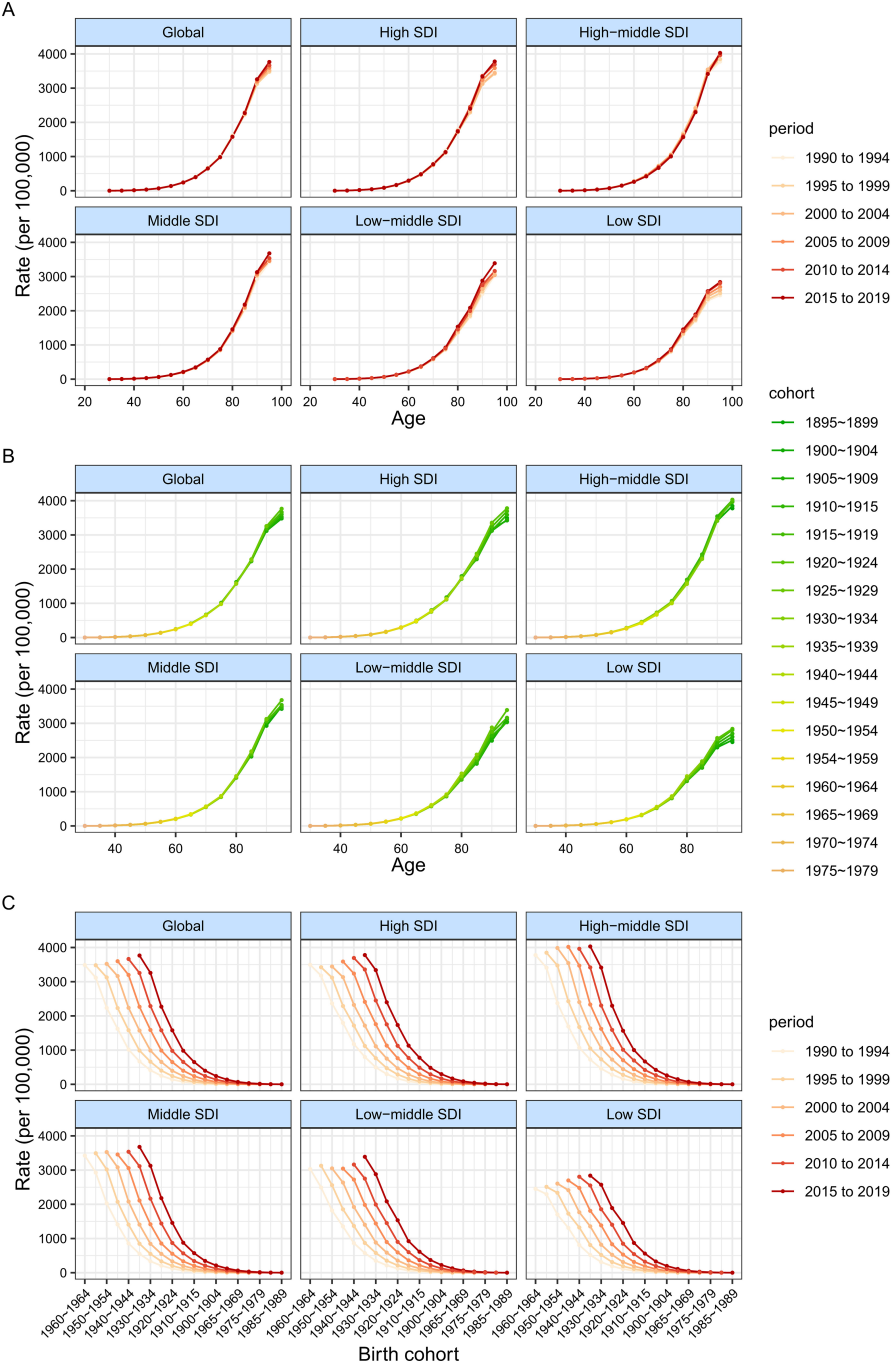
DALY rates of atrial fibrillation and flutter across different age groups, periods and birth cohorts during 1990–2019. **(A)** DALY rates of atrial fibrillation and flutter across age groups by periods. **(B)** DALY rates of atrial fibrillation and flutter across age groups by birth cohorts. **(C)** DALY rates of atrial fibrillation and flutter across abirth cohorts by periods.

Figure 3 presents the estimates obtained from the APC model, highlighting the impacts of age, period, and cohort based on SDI quintiles. Conversely, Figure 4 displays the rates of DALY for AF/AFL across various age categories, time periods, and birth cohorts from 1990 to 2019. To be more specific, the picture shows how age affects the DALYs linked to AF/AFL by showing longitudinal age curves that show how this age-related condition normally changes over time. Additionally, we display the period impacts as the relative risk of DALYs across multiple time periods, allowing for the tracking of progress over time. Finally, we show the cohort effects as the comparative risk of DALYs among cohorts, enabling the monitoring of changes in DALYs for various birth cohorts.

Overall, the age effect exhibited a similar pattern across different SDI quintiles, with the ratio of DALYs increasing as age advanced. In countries with a higher SDI, there were higher DALYs rates across all age groups over 80 years. This can be attributed to the overall higher standard of medical care and greater awareness of healthcare in these countries. Additionally, the average life expectancy for the entire population is longer compared to countries with a lower SDI.

During the study period, the number of DALYs linked to AF/AFL first slowed down and then slowly began to rise globally and in countries with high SDI. Conversely, countries with a high-middle SDI often had a decline in DALY ratios. In contrast, countries with middle SDI, low-middle SDI, and low SDI had an increase in DALY ratios. Moreover, it is important to highlight that the rate of DALYs for males in nations with middle and low-middle SDI is rising at a much faster rate than that of females.

Globally, the risk of DALYs across different birth cohorts demonstrated a predominantly steady trend, with minor fluctuations observed throughout the period. While the risk in lower SDI nations has been increasing since the 1910 cohort, individuals born after the 1910s in high-middle SDI countries saw gradual improvements in DALYs. Supplementary Figures S16–S54 show how age, period, and cohort affect DALYs associated with AF/AFL in each specific nation.

To illustrate the primary trends in DALYs associated with AF/AFL, we have included representative countries from various SDI quintiles. This selection allows for a more comprehensive characterization of the age-period-cohort effects on AF/AFL DALYs globally. Japan and Bermuda exemplify the trends observed in high SDI countries, where DALYs showed a decline across all age groups. Furthermore, this decline was more pronounced in women compared to men, with favourable decreases in both period and cohort risks. Turkey, categorised as a high-middle SDI country, exhibited a distinct pattern with a notable net drift. However, it is worth noting that the reduction in DALYs was somewhat attenuated in adults over the age of 80. It is noteworthy that Burundi, despite being categorised as a low SDI country, demonstrates a gradual decline in the relative risk of DALYs across different time periods and birth cohorts.

Furthermore, among the high-middle SDI countries, Bahrain exhibited the most concerning trends in terms of DALYs related to AF/AFL. The country experienced local drifts exceeding 0% per year across all age groups, indicating a worsening situation. At the same time, we note that the period and cohort risks in Bahrain continue to worsen, highlighting the seriousness of the problem. In Sweden, there was a prominent trend of increasing DALYs among individuals aged 30 years and older. This upward trend was characterised by the intensification of both period and cohort effects throughout the study period, until recent years, when the rate of increase started to decrease. In Uzbekistan and Ecuador, the trends in both period and cohort effects over the past three decades have shown a consistent and concerning pattern of increase, suggesting a lack of improvement. Efforts to enhance the management of AF/AFL could be strengthened in the following ways: 1. Elevating Public Awareness: Further raise awareness of AF/AFL across society by collaborating extensively to disseminate AF/AFL concepts. Extend awareness initiatives to county-level, grassroots hospitals, and remote areas. Expand these efforts nationwide, reaching all regions and patient populations. This comprehensive approach aims to ensure that the public has a thorough understanding of AF. Once individuals grasp knowledge about AF, they are more likely to prioritise early diagnosis, actively seek medical advice, understand related complications, and actively seek treatment, thus increasing the rate of medical consultations. 2. Utilising Advanced Diagnostic Methods: Traditional methods such as electrocardiograms, Holter monitors, and auscultation can be effective in preliminary screening or diagnosing AF/AFL. In the era of internet-based healthcare, wearable smart devices offer a quicker and more convenient means of diagnosis. These emerging diagnostic methods may have more practical value than traditional approaches. Unlike the former, they can promptly record the occurrence of AF/AFL or capture AF/AFL events, synchronising this information to the internet and significantly enhancing the detection rate of AF/AFL. 3. Professional Training and Education: Provide standardised and scientifically-oriented education on AF/AFL management for professionals. Enhance the training of personnel involved in AF/AFL management. Ensure they have a comprehensive understanding of various AF/AFL screening techniques tailored for different population groups. This includes techniques like electrocardiograms, Holter monitors, long-term cardiac monitoring, implantable cardiac monitoring devices, and portable devices widely accessible to the general population. 4. Active Participation in International Exchanges: actively engage in international exchanges to introduce and adopt advanced experiences in the diagnosis and management of AF/AFL.5. Focus on Understanding Diseases in the Elderly: Strengthen awareness of disease characteristics specific to the elderly population. Advocate for the establishment of dedicated records for AF/AFL patients, emphasising the importance of specialised management. Provide assistance and encourage family members to undergo AF/AFL-related education and training to assist in the management of AF/AFL in elderly patients.

## Discussion

Our study provides a comprehensive global assessment of the age-period-cohort effects on DALYs associated with AF/AFL, highlighting its significant impact on global public health. In 2019, the burden of AF/AFL and its temporal trends between 1990 and 2019 showed significant disparities with respect to gender, SDI quintile, and geographic region. Interestingly, the DALY growth over the past 30 years did not consistently align with a country's socio-economic development, despite a declining trend in the ASR of DALYs globally. The existence of unfavourable period and cohort effects indicates that it is necessary to reassess and reposition the current preventative and treatment measures for AF/AFL. It is crucial to adopt more efficient methods and therapies that focus on changeable risk factors, particularly in areas with a significant or growing prevalence of AF/AFL. This highlights the urgent need for focused efforts to address this persistent global health challenge.

Gaining global insight into the long-term trends of AF/AFL illness is essential for developing successful preventative strategies and improvements in healthcare. Previous research has investigated the global impact of AF/AFL using data from the GBD study in 2017 and 2019. However, these studies did not clearly explore the individual influences of age, period, and cohort impacts on DALYs (7–9, 14, 18–20). Drawing upon high-quality data, this study offers a comprehensive and updated epidemiological analysis of spatio-temporal trends in DALYs associated with AF/AFL. The analysis covers all age groups over 30 and encompasses global, regional, and national levels from 1990 to 2019. The findings indicate a persistently high and escalating burden of AF/AFL on a global scale, with variations observed across different SDI categories. Over the past 30 years, the number of DALY cases for AF/AFL has nearly doubled—a 2.2-fold increase. Population growth, longer lifespans, and increased exposure to important risk factors such as high systolic blood pressure, a high body mass index, alcohol consumption, salt consumption, and lead exposure may be contributing to the rise in AF/AFL DALYs cases. These factors have led to a higher prevalence of AF/AFL despite a decrease in incidence and improved survival rates in some higher SDI countries (7–9, 14, 21, 22). Additionally, it is important to acknowledge that the quality of data can vary significantly across countries. Countries with a low SDI and politically volatile areas, such as North Africa, the Russian Federation, and the Middle East (including Libya and Syria), constrain epidemiological research. This can introduce uncertainties in data reliability. Additionally, there are difficulties in using sub-national data to make estimations for the whole country, which further affects the accuracy of AF/AFL calculations based on these primary statistics. Additional primary investigations should validate our conclusion regarding the lack of significant enhancement in AF/AFL DALYs. If there is a strong link between differences in how well healthcare works for this group and the SDI, then the actual drops in the number of years lost to atrial fibrillation and atrial flutter (AF/AFL DALYs) do not match what would be expected based on a country's socioeconomic status. For instance, countries like Sweden, Bahrain, and Burundi exhibit variations that deviate from the anticipated trends.

To the best of our knowledge, this study represents the first application of APC models on a global scale to analyse trends in AF/AFL DALYs, allowing for meaningful comparisons between different countries. Specifically designed to study and solve collinearity problems between age, period, and cohort effects is the APC model, a multivariate analysis method. These three dimensions often intertwine and influence trends in health and disease in epidemiological and demographic studies. The model, through its structured design, is able to distinguish the independent effects of age, period, and cohort, even though these variables are highly correlated mathematically (13). This is a challenge for traditional statistical methods, which may fail to accurately estimate the effects of each variable due to collinearity (13). In addition, the APC model allows researchers to consider the effects of multiple dimensions simultaneously within the same framework, providing an integrated approach to analysis rather than considering each factor in isolation. At the same time, APC models can reveal trends over time, including how populations (cohorts) born in different decades exhibit different health conditions at certain ages over time (periods). Of course, one can flexibly apply the APC model to various data structures and research questions, be it an analysis of an entire population or a specific health condition. In comparison to the previous GBD 2019 publication, our study provides a comprehensive and in-depth analysis of the field, offering detailed insights into disease trends and valuable implications for public health. A key strength lies in our examination of period and cohort effects, enabling us to identify the underlying factors contributing to DALYs trends within specific time periods and birth cohorts in each country. This information is crucial for assessing the effectiveness of AF/AFL-related healthcare services. Additionally, an important advancement in our study is the estimation of local drift values, enabling us to capture time trends in DALYs for each age group while accounting for period effects (12, 17).

Several factors contribute to the lower burden of illness in low-income countries, including age-standardised prevalence, mortality, and DALYs. These include younger population demographics, higher birth rates, and limited access to essential healthcare interventions necessary for basic survival. Consequently, the overall burden of illness remains considerably lower compared to estimates that encompass all age groups (23). Hence, relying solely on age-standardised rates to monitor trends in AF/AFL DALYs can be misleading, particularly for regions with a lower SDI. Moreover, evaluating changes based on an overall rate overlooks valuable information regarding variations across different age groups, time periods, and birth cohorts. In our study, we looked at AF/AFL DALYs using age-standardised rates and estimates from the APC model to get a full picture of how AF/AFL DALYs are changing over time. This approach allowed us to gain deeper insights into the patterns and dynamics of AF/AFL DALYs.

The literature reports that the peak occurrence of DALYs attributed to AF/AFL occurred in males between the ages of 70 and 74 and in females between the ages of 80 and 84. Additionally, the number of DALYs was higher in males than in females within the same age group until the 70–74 age range (7, 8, 14). Moreover, age-specific DALYs for AF/AFL increased with age in both males and females. Li and colleagues observed gender differences across various age groups (9). Specifically, they found that females exhibited a higher overall incidence of the condition compared to males after the age of 65. Additionally, females have a greater prevalence than males after reaching 75 years of age. Furthermore, females experience a significantly higher number of deaths than males beyond the age of 65, and they also have a higher number of DALYs than males after the age of 70. According to their findings, the increased number of DALYs in women compared to men could be attributed to several factors, including the diminished quality of life experienced by female patients and income disparity between men and women, which leads to limited access to healthcare for women (9). Moreover, the study highlights that female AF/AFL patients tend to utilise rhythm control strategies less frequently than their male counterparts (24, 25). This includes less use of antiarrhythmic therapy, cardioversion, and catheter ablation. In light of these observations, the study suggests the need for greater emphasis on rhythm control strategies and the treatment of complications in female AF/AFL patients. By addressing these aspects, healthcare providers can work to reduce the burden of disability and improve outcomes for female patients with AF/AFL. To address these gender inequalities, further research and a rational allocation of resources are imperative. Nevertheless, our research reveals a global trend where the number of DALYs attributed to AF/AFL is progressively rising with advancing age. Furthermore, within the same age group, the number of DALYs is greater in males compared to females. Age, surpassing other factors like gender, smoking, alcohol consumption, hypertension, body mass index, major heart murmur, left ventricular hypertrophy, myocardial infarction, and heart failure, is the most significant risk factor for the development of AF/AFL, according to 50-year research. Our investigation revealed that the burden of DALYs due to AF/AFL was most pronounced among older individuals. In particular, the large increase in DALYs linked to AF/AFL in older people suggests that the current care for AF/AFL in this age group is not enough.

In the ASR of DALYs trends of AF/AFL, there was a lot of variance between SDI quintiles, consistent with the GBD 2017 study on AF/AFL (14). The impact of AF/AFL is growing, albeit to different degrees, across every region, according to recent research (26). However, in developed regions, the overall rates of DALYs associated with AF/AFL tend to decrease. Several factors contribute to this trend. Firstly, developed regions tend to receive a higher proportion of resources, allowing for better allocation of healthcare facilities and services. These regions generally have more robust healthcare systems, allowing for better management and prevention of AF/AFL and its complications. Secondly, patients in developed regions often have higher levels of education, making them more aware and proactive in seeking treatment and taking preventive measures for AF/AFL.

In population areas with high and high-middle SDI ratings, population ageing has become the most important demographic variable. Areas with poor SDI ratings, on the other hand, had short life expectancy and high fertility rates, which were the main drivers of population growth (9). It is gratifying to see that epidemiological improvements have had a favourable effect on low and low-middle SDI regions by evaluating the patterns of ASR of DALYs. Conversely, there seems to be some success in the fight against AF/AFL in areas with high and high-middle SDI, since these areas show a negative correlation with epidemiological trends. This study highlights the fact that AF/AFL is a serious issue in economically disadvantaged areas. In this regard, it is important to objectively understand that several circumstances typically result in a significantly lower DALY for AF/AFL than the actual situation. Undiagnosed Cases: Some individuals may have atrial fibrillation, but due to the absence of obvious symptoms or a lack of cardiac monitoring, their condition remains undiagnosed in a timely manner. Asymptomatic Cases: Certain individuals may have atrial fibrillation without experiencing apparent symptoms. Since atrial fibrillation can be asymptomatic, patients may not actively seek medical assistance, leading to an underestimation of the disease burden. Inequality in Healthcare Services: In certain regions or demographic groups, inadequate healthcare resources or uneven distribution may result in some patients being unable to receive timely and appropriate diagnosis and treatment. Sociocultural Factors: In some cultures, cultural perceptions or attitudes towards healthcare may exist, influencing individuals’ willingness to seek medical attention or undergo cardiac health screening. Incomplete Health Records: Occasionally, patients’ health records may be incomplete or inadequately documented, contributing to inaccuracies in statistical data. Similarly, various factors could explain the higher DALY rates among individuals in high SDI countries compared to those in low SDI countries: Ageing Population: High SDI countries typically have a higher life expectancy, resulting in a larger elderly population. Atrial fibrillation is age-related, with a higher risk among the elderly. Therefore, the prevalence of atrial fibrillation is likely higher in countries with high SDI. High-SDI countries usually have advanced and widely accessible healthcare systems, better healthcare infrastructure, and increased opportunities for individuals to access medical services. This can lead to a more timely diagnosis and recording of atrial fibrillation cases. Lifestyle Factors: Individuals in high SDI countries generally adopt healthier lifestyles, including better dietary habits, increased physical activity, and fewer detrimental habits. These factors can influence cardiovascular health, including the prevalence of atrial fibrillation. Widespread Health Education: High-SDI countries may implement extensive health education programmes, enhancing public awareness of cardiac health. This could prompt more individuals to seek medical assistance, thereby increasing the detection and reporting of atrial fibrillation. In summary, understanding the complex interplay of these factors is crucial for a comprehensive interpretation of the observed differences in atrial fibrillation burden across regions and socioeconomic indices. Today, it is crucial for national and regional health departments to aggressively seek ways that focus on health, education, and economics when managing AF/AFL. Modifiable variables such as elevated systolic blood pressure, increased body mass index, salt-rich diets, alcohol use, and smoking may account for a considerable proportion of the disease burden associated with AF/AFL, including mortality and DALYs (9). We should tailor preventive and control strategies to the specific attributes of each location, taking into account the diverse risk factors encountered by nations with varying socio-economic statuses.

Based on this study's findings, we propose the following recommendations for the management and control of AF/AFL in countries with a lower SDI: 1. Promote the Construction of Basic Healthcare Facilities: In countries with lower SDI, the construction of basic healthcare facilities is crucial. Governments can formulate policies and plans to improve the coverage of medical facilities, including the establishment of more hospitals, clinics, and health centres, to ensure that more patients have access to diagnostic and treatment services. 2. Improve medical personnel training: Governments can encourage more healthcare professionals to undergo training in the diagnosis and treatment of cardiac arrhythmias, including the identification, management, and treatment of AF/AFL, by providing training programmes and incentive mechanisms. 3. Develop diagnostic and treatment guidelines and standards. Develop diagnostic and treatment guidelines and standards applicable to low-resource environments to guide healthcare professionals in the best practices for diagnosing and treating AF/AFL. These guidelines should take into account local resource and facility constraints, as well as provide simplified diagnostic methods and cost-effective treatment options. 4. Promote Electrocardiogram (ECG) Technology: The ECG is an important tool for diagnosing AF/AFL. Governments can invest in promoting ECG technology, which includes providing ECG equipment, training technical personnel, and establishing remote ECG diagnostic services, to ensure that more patients have access to ECG examinations. 5. Strengthen Patient Education and Screening: Conduct patient education and screening activities for AF/AFL to raise public awareness of cardiac arrhythmias and encourage high-risk populations to undergo regular cardiac arrhythmia screening and examination. 6. Encourage Drug Therapy and Non-drug Treatment Methods: Through policy support and partnerships, governments can promote the accessibility and affordability of drug therapy and non-drug treatment methods, including anticoagulant therapy, antiarrhythmic drugs, electrophysiological treatment, and surgical treatment. 7. Set up tracking and monitoring systems: For patients with cardiac arrhythmias, establish tracking and monitoring systems to promptly detect and intervene in changes in patients’ conditions, thereby improving treatment effectiveness and patient quality of life. These policy and practice recommendations can help improve AF/AFL management and control in countries with lower SDI, reduce the burden of cardiovascular diseases, and enhance patients’ quality of life. However, achieving these goals requires collaboration among governments, healthcare institutions, healthcare professionals, and communities to collectively promote the improvement of cardiac arrhythmia management.

Direct oral anticoagulants (DOACs) are very important for treating AF/AFL and venous thromboembolism (VTE). They work better than vitamin K antagonists (VKAs) at stopping thrombotic events and intracerebral haemorrhage (ICH) (27). These benefits include fewer drug interactions and less monitoring. However, drug-drug interactions, the need for adjustments during surgery or invasive procedures, and considerations for patients with kidney, liver disease, or cancer are significant challenges. Patient adherence is critical for effectiveness, and improved understanding and adherence can lead to better outcomes. DOACs also lower the disability-adjusted life years (DALY) burden in AF patients by better preventing thrombotic events and intracranial bleeding. Their ease of use may make people more likely to stick with their treatment, which lowers DALYs even more (28).

The complex relationship between AF/AFL and ICH presents clinical dilemmas (29). AF/AFL increases the risk of ischemic stroke, prompting the use of oral anticoagulants (OACs) such as VKAs or DOACs, while also increasing the risk of ICH. The decision to initiate or resume OAC therapy after ICH is challenging due to the risk of ICH recurrence vs. the risk of ischemic stroke without OACs. While randomised controlled trials (RCTs) often exclude patients with recent ICH, observational studies suggest that OACs reduce stroke incidence and mortality post-ICH. Delaying anticoagulant therapy may lower the risk of recurrent ICH, but it could also increase the risk of ischemic events. Tailored risk assessment is crucial for determining the optimal timing for anticoagulation. For high-risk ICH patients, left atrial appendage occlusion (LAAO) may offer an alternative to reduce thrombosis and reliance on OACs. Considering the impact on DALYs, a multidisciplinary approach is essential for managing AF/AFL post-ICH, as ICH heightens the risk of death, disability, and reduced quality of life, thus increasing the DALY burden. Balancing thromboembolic event prevention with ICH risk reduction is critical to minimizing DALYs for AF/AFL patients.

Reversal agents play a vital role in managing AF/AFL, particularly in emergencies. They rapidly counteract DOAC-induced anticoagulation in major bleeding incidents, reducing the risk of death and complications (30). Clinical trials have validated their effectiveness and safety, providing an essential tool for clinicians. However, adherence to guidelines is suboptimal, indicating a need for enhanced education and training for physicians to ensure proper use in emergencies.

There are several key factors to consider when interpreting our findings in relation to public health. Firstly, it is important to exercise caution when interpreting estimates for low- and middle-income countries (LMICs) where primary data is lacking, such as Papua New Guinea, Yemen, and Sudan. These estimates are based on modelling and would benefit from validation through independent primary studies. Secondly, our analysis relied on country-level DALY estimates as input for the APC model. It is critical to recognize the importance of not directly extrapolating our study's conclusions to regions within countries. Healthcare disparities and variations in health outcomes persist within countries, leading to differing levels of progress across different regions. Considering these factors is critical for a comprehensive understanding of our research's implications in the context of public health. Our study serves as an example of how to conduct a detailed analysis of disease trends using data from the GBD study. We can extend our approach to analyse disease trends associated with other non-communicable diseases (NCDs). By using APC models to analyse trends over different ages, periods, and cohorts, we may get a more comprehensive understanding of how well the health system has responded, beyond the limitations of standard epidemiological measures. This approach may make it easier to track how each nation is doing in terms of meeting the Sustainable Development Goals (SDGs).

We must acknowledge the study's several limitations. First, our analyses are limited by the GBD models, which use estimates from data from settings with more resources and limited primary data from LMICs (31). For many countries with high DALYs, there is a lack of primary data on AF/AFL, resulting in wide uncertainty bounds in GBD DALY estimates due to heavy reliance on covariates (32). This has the potential to affect the precision of estimates for age, period, and cohort trends, and it may lead to an overestimation of improvements in some low SDI countries. We urgently need to obtain better primary data on AF/AFL DALYs in LMICs, including longer-term cohort studies. Secondly, the research primarily examines the epidemiological aspects of AF/AFL and does not fully explore the socioeconomic disparities across different groups. More focused investigations are required to determine the weight of the many variables that cause these discrepancies. Additionally, the GBD may not adequately reflect regional disparities in economic development within countries, thereby limiting the overall representation of certain regions or countries. Thirdly, despite advancements in diagnostic techniques for AF/AFL, a significant number of asymptomatic or sporadically occurring instances in the population may remain undiagnosed. This might result in an inaccurate calculation of the true number of AF/AFL cases (14, 33, 34). According to X.-J. Dong et al., the age-adjusted prevalence of AF/AFL went down from 1990 to 2019. However, improvements in diagnostic technologies, greater awareness of AF/AFL, population growth, and aging may have led to an overall rise in numbers. It's also possible that the sharp rise in the number of AF/AFL DALY cases is due to people not getting diagnosed with asymptomatic or intermittent AF/AFL and the lack of standardised treatment. Furthermore, the growth rate of DALY cases for AF/AFL surpasses that of prevalence. Therefore, there is an urgent need not only for early AF/AFL detection but also for the development of more effective treatment measures to actively manage AF/AFL (14). Fourth, AF/AFL encompasses different subtypes, such as paroxysmal, persistent, and permanent forms. However, the GBD database does not account for this complexity, and it only recognises the presence of AF/AFL in the population without differentiating subtypes. This limitation prevents us from stratifying the analysis according to specific clinical subtypes. Fifth, we did not modify the current data analysis to account for any possible publication bias, as we did not attempt to obtain unpublished sources. This might potentially lead to a bias in the outcomes. Finally, the research is susceptible to the overall constraints outlined by the GBD cooperation group (8, 35–37). Differences in races, nationalities, and socioeconomic systems within nations might influence the amount of information included in the database and perhaps affect the precision of the projected outcomes.

## Data Availability

Publicly available datasets were analyzed in this study. This data can be found here: the GBD 2019 database.
