# Supplementary material for: Differences in global, regional, and national time trends in disability-adjusted life years for atrial fibrillation and flutter, 1990–2019: an age-period-cohort analysis from the 2019 global burden of disease study
Source: Front Cardiovasc Med. 2024 Aug 29;11:1401722. doi: 10.3389/fcvm.2024.1401722 (PMC11390633; doi:10.3389/fcvm.2024.1401722)
Supplement: Supplementary file 1 [file Table1.pdf]

Table S1. Trends in atrial fibrillation and flutter DALY for both sexes in 204 countries, 1990-2019

Age-standardised DALY rate is computed by direct standardisation with global standard population in GBD 2019. Net drifts are estimates derived from the age-period-cohort model and denotes overall annual percentage change in DALY, which captures the contribution of the effects from calendar time and successive birth cohorts. SDI=Socio-demographic Index; APC=age-period-cohort;AAPCs=Average annual percentage changes.

location	case_1990	case_2019	case_percent_change	ASR_1990	ASR_2019	ASR_percent_change	AAPC	netdrift
Afghanistan	4052 (3019, 5410)	7079 (5364, 9386)	74.71 (51.76, 100.02)	69.57 (51.66, 91.18)	76.41 (59.48, 98.79)	9.82 (-4.5, 24.46)	0.33 (0.3, 0.35)	0.27 (0.08, 0.45)
Albania	2174 (1650, 2809)	5202 (3928, 6861)	139.31 (113.92, 168.62)	117.77 (90.7, 149.63)	120.14 (91.29, 157.68)	2.02 (-8.45, 14.12)	0.07 (-0.01, 0.15)	0.1 (-0.21, 0.41)
Algeria	7956 (6164, 10067)	24352 (18956, 30290)	206.1 (162.2, 257.37)	92.14 (73.11, 115.68)	91.18 (71.78, 111.62)	-1.04 (-16.16, 16.42)	-0.02 (-0.09, 0.04)	-0.02 (-0.23, 0.18)
American Samoa	24 (19, 29)	56 (44, 69)	134.97 (107.92, 167.17)	127.49 (103.53, 157.99)	131.02 (104.81, 162.47)	2.77 (-8.76, 16.02)	0.11 (0.03, 0.19)	0.15 (-1.67, 2)
Andorra	69 (50, 92)	197 (146, 259)	184 (136.84, 244.21)	142.87 (106.75, 187.56)	133.72 (99.85, 176.65)	-6.4 (-22.06, 10.65)	-0.24 (-0.3, -0.19)	-0.46 (-2.03, 1.14)
Angola	2377 (1743, 3054)	7878 (5868, 9853)	231.36 (171.34, 308.56)	84.07 (61.56, 108.39)	99.37 (74.9, 123.73)	18.2 (-3.68, 45.71)	0.57 (0.55, 0.59)	0.41 (0.2, 0.62)

Antigua and Barbuda	55 (46, 65)	100 (84, 121)	82.05 (59.17, 106.13)	95.99 (81.48, 115.08)	114.01 (96.92, 139.06)	18.76 (4.19, 34.21)	0.66 (-0.03, 1.35)	0.09 (-1.74, 1.95)
Argentina	27479 (22489, 34047)	53856 (44272, 66837)	95.99 (81.14, 112.46)	92.61 (76.56, 113.49)	96.73 (79.44, 120.36)	4.45 (-3.5, 13.06)	0.23 (0.07, 0.39)	0.04 (-0.06, 0.14)
Armenia	2492 (1843, 3318)	4990 (3809, 6604)	100.27 (82.95, 123.6)	105.21 (79.34, 138.6)	122 (93.57, 160.53)	15.95 (5.02, 28.91)	0.42 (0.24, 0.6)	0.42 (0.13, 0.71)
Australia	35100 (27762, 44428)	75139 (59551, 95423)	114.07 (102.82, 130.91)	183.86 (146.41, 231.11)	167.01 (131.41, 213.42)	-9.16 (-13.75, -2.62)	-0.33 (-0.39, -0.27)	-0.33 (-0.42, -0.24)
Austria	17609 (13912, 22564)	31779 (24891, 40121)	80.48 (46.26, 97.81)	143.07 (112.76, 182.68)	163.04 (126.86, 207.93)	13.95 (-7.71, 25.12)	0.55 (0.28, 0.82)	0.74 (0.6, 0.89)
Azerbaijan	4796 (3543, 6300)	9149 (6883, 12077)	90.75 (70.4, 118.87)	106.79 (78.98, 140.22)	131.39 (103.7, 165.3)	23.03 (8.72, 46.71)	0.71 (0.53, 0.88)	0.63 (0.45, 0.81)
Bahamas	132 (109, 159)	355 (293, 441)	169.1 (132.75, 213.24)	100 (83.33, 120.14)	104.43 (86.3, 129.47)	4.43 (-9.54, 21.49)	0.3 (-0.26, 0.86)	0.19 (-0.63, 1.01)
Bahrain	121 (98, 151)	946 (717, 1174)	679 (455.35, 864.43)	100.24 (82.24, 126.38)	165.78 (129.43, 198.77)	65.37 (14.2, 105.21)	1.84 (1.27, 2.42)	1.87 (0.85, 2.91)
Bangladesh	35235 (26123, 45690)	118576 (88486, 151181)	236.53 (182.27, 300.39)	92.04 (68.78, 118.86)	105.11 (78.77, 133.15)	14.2 (-4.42, 36.13)	0.4 (0.13, 0.67)	0.4 (0.33, 0.47)
Barbados	257 (213, 314)	456 (376, 561)	77.28 (53.57, 98.51)	83.58 (69.51, 101.03)	92.33 (76.17, 113.36)	10.47 (-4.41, 23.78)	0.26 (0.2, 0.33)	0.09 (-0.88, 1.07)
Belarus	14511 (10600, 19262)	22656 (16908, 29587)	56.13 (38.2, 75.44)	114.42 (84, 151.57)	138.54 (103.48, 181.55)	21.08 (7.18, 35.46)	0.65 (0.59, 0.72)	0.58 (0.44, 0.73)
Belgium	20500 (16428, 25943)	30619 (25209, 37943)	49.36 (35.87, 61.58)	129.91 (104.26, 164.12)	119.42 (96.46, 149.63)	-8.07 (-15.91, -1.53)	-0.31 (-0.39, -0.23)	-0.28 (-0.41, -0.15)
Belize	63 (50, 80)	201 (167, 247)	219.09 (160.33, 269.66)	71.7 (57.21, 90.73)	83.32 (69.27, 101.13)	16.2 (-4.88, 34.2)	0.45 (0.01, 0.88)	0.45 (-0.75, 1.67)

Benin	1255 (933, 1588)	3063 (2442, 3764)	144.15 (112.13, 187.25)	75.12 (56.18, 94.07)	83.71 (67.66, 102.29)	11.44 (-4.53, 32.29)	0.37 (0.34, 0.41)	0.28 (-0.04, 0.59)
Bermuda	54 (44, 66)	108 (88, 134)	100.06 (74.79, 134.51)	96.93 (78.94, 116.98)	76.91 (62.35, 95.72)	-20.66 (-31.05, -7.75)	-0.77 (-0.85, -0.69)	-1.01 (-3.25, 1.29)
Bhutan	168 (121, 226)	539 (403, 691)	221.88 (166.4, 296.03)	91.26 (66.96, 122.65)	111.59 (83.06, 143.06)	22.27 (-0.1, 49.46)	0.7 (0.67, 0.73)	0.46 (-0.38, 1.3)
Bolivia (Plurinational State of)	1969 (1467, 2734)	6739 (5107, 8988)	242.24 (168.72, 335.36)	77.6 (58.68, 105.57)	92.97 (70.89, 121.81)	19.8 (-6.03, 50.77)	0.6 (0.57, 0.62)	0.2 (-0.02, 0.43)
Bosnia and Herzegovina	4708 (3573, 6093)	8538 (6614, 10978)	81.35 (61.25, 109.75)	133.26 (103.75, 169.82)	145.72 (113.24, 186.08)	9.35 (-2.24, 26.24)	0.21 (-0.12, 0.53)	0.28 (0.05, 0.52)
Botswana	319 (243, 403)	817 (625, 1036)	156.05 (107.29, 213.12)	72.28 (56.44, 89.83)	79.07 (60.67, 100.19)	9.39 (-12.05, 35.07)	0.28 (0.19, 0.37)	-0.05 (-0.57, 0.47)
Brazil	71421 (58908, 88622)	230116 (189167, 279886)	222.2 (190.45, 239.67)	98.67 (81.8, 121.28)	102.5 (84.33, 124.48)	3.88 (-6.41, 8.67)	0.26 (0.06, 0.45)	0.5 (0.43, 0.58)
Brunei Darussalam	96 (78, 117)	265 (224, 315)	176.92 (127.52, 224.92)	132.29 (110.09, 158.33)	127.1 (110.49, 146.41)	-3.92 (-20.06, 13.05)	-0.11 (-0.63, 0.42)	-0.2 (-1.28, 0.88)
Bulgaria	16507 (12214, 22426)	23545 (18078, 31008)	42.64 (24.86, 65.78)	145.4 (109.8, 195.57)	156.83 (120.82, 204.6)	7.86 (-5.53, 23.55)	0.28 (0.06, 0.51)	0.25 (0.11, 0.39)
Burkina Faso	2467 (1798, 3219)	6470 (4941, 8115)	162.28 (125.59, 205.84)	76.77 (55.47, 99.5)	97.29 (75.09, 121.7)	26.73 (7.07, 49.2)	0.89 (0.73, 1.05)	0.8 (0.56, 1.04)
Burundi	1548 (1047, 2115)	2222 (1553, 2911)	43.48 (7.76, 84.72)	81.77 (54.57, 111.24)	67.89 (46.25, 88.39)	-16.97 (-36.34, 5.59)	-0.68 (-0.81, -0.56)	-0.97 (-1.26, -0.68)
Cabo Verde	171 (135, 211)	414 (336, 496)	142.42 (103.54, 201.9)	70.79 (56.2, 87.73)	100.8 (81.8, 121.1)	42.39 (19.63, 75.93)	1.33 (1.11, 1.55)	0.52 (-0.55, 1.6)
Cambodia	2943 (2120, 3891)	9447 (7052, 12396)	220.99 (188.05, 254.64)	82.06 (60.63, 107.52)	95.56 (72.43, 124.5)	16.45 (4, 29.34)	0.53 (0.5, 0.55)	0.43 (0.25, 0.62)

Cameroon	2730 (2043, 3540)	8128 (6452, 9994)	197.71 (145.69, 272.11)	85.91 (64.61, 109.4)	97.63 (78.11, 119.12)	13.64 (-7.75, 43.69)	0.44 (0.34, 0.53)	0.43 (0.23, 0.63)
Canada	47742 (36228, 62767)	103817 (78062, 136908)	117.46 (106.88, 128.28)	147.9 (112.36, 193.6)	142.69 (106.55, 188.65)	-3.52 (-8.31, 1.55)	-0.14 (-0.19, -0.08)	-0.22 (-0.29, -0.15)
Central African Republic	826 (566, 1094)	1465 (1043, 1934)	77.35 (46.34, 119.06)	98.19 (67.53, 129.14)	97.78 (69.1, 127.48)	-0.41 (-17.45, 22.1)	-0.02 (-0.1, 0.06)	-0.06 (-0.47, 0.35)
Chad	1680 (1189, 2186)	3320 (2538, 4230)	97.63 (69.67, 130.62)	72.55 (51.25, 94.33)	79.93 (61.33, 100.2)	10.16 (-5.87, 30.36)	0.34 (0.32, 0.36)	0.35 (0.05, 0.64)
Chile	9467 (7755, 11959)	27026 (22203, 33915)	185.49 (168.76, 203.18)	109.91 (91.28, 136.9)	112.55 (92.57, 141.1)	2.4 (-3.09, 8.09)	0.05 (-0.09, 0.19)	0.17 (0.04, 0.3)
China	631474 (475313, 824192)	1729840 (1309686, 2226445)	173.94 (147.86, 199.51)	98.61 (76.85, 124.84)	97.08 (75.21, 123.12)	-1.56 (-11.61, 8.1)	-0.08 (-0.15, -0.02)	-0.04 (-0.08, 0)
Colombia	10167 (8236, 13208)	38546 (30514, 48095)	279.12 (209.85, 346.84)	69.85 (57.07, 90.51)	70.54 (55.84, 88.37)	0.99 (-17.81, 18.17)	-0.07 (-0.36, 0.22)	-0.06 (-0.16, 0.04)
Comoros	137 (94, 177)	302 (226, 372)	119.96 (76.41, 193.75)	74.94 (51.37, 95.54)	72.7 (54.24, 89.34)	-2.99 (-21.91, 25.17)	-0.07 (-0.14, 0.01)	-0.32 (-1.26, 0.63)
Congo	908 (670, 1166)	2164 (1682, 2658)	138.4 (89.16, 197.99)	113.78 (83.83, 144.52)	113.56 (90.29, 139.8)	-0.2 (-19.27, 24.5)	0.06 (-0.06, 0.17)	-0.2 (-0.6, 0.21)
Cook Islands	14 (11, 18)	30 (24, 38)	107.04 (77.25, 143.28)	128.6 (101.85, 159.51)	124.97 (99.65, 157.03)	-2.82 (-16.87, 14.66)	-0.07 (-0.16, 0.02)	-0.1 (-2.63, 2.48)
Costa Rica	1087 (881, 1334)	3733 (2930, 4709)	243.49 (199.32, 300.34)	67.87 (55.02, 83.02)	73.08 (57.4, 92.39)	7.68 (-5.99, 25.17)	0.21 (-0.12, 0.55)	0.09 (-0.26, 0.44)
Croatia	7006 (5383, 9174)	9496 (7231, 12316)	35.54 (23.07, 51.88)	116.02 (90.29, 150.78)	103.77 (78.57, 135.05)	-10.56 (-18.26, -1.13)	-0.44 (-0.57, -0.32)	-0.26 (-0.49, -0.02)
Cuba	7184 (5864, 8884)	15261 (12276, 18913)	112.44 (83.82, 145.94)	73.17 (59.95, 90.27)	75.58 (60.87, 94.11)	3.31 (-10.14, 19.24)	0.14 (-0.02, 0.31)	-0.09 (-0.26, 0.08)

Cyprus	1248 (995, 1540)	2775 (2280, 3420)	122.42 (96.05, 154.31)	177.96 (143.39, 215.59)	150.07 (124.21, 182.95)	-15.67 (-27.4, -3.29)	-0.67 (-0.93, -0.42)	-0.88 (-1.35, -0.4)
Czechia	18729 (14399, 24570)	29179 (22356, 38060)	55.8 (37.92, 72.01)	137.03 (105.7, 177.5)	133.84 (102.33, 174.44)	-2.33 (-13.26, 7.16)	0.16 (-0.1, 0.42)	0.27 (0.13, 0.41)
C 么 te d'Ivoire	1980 (1489, 2555)	5979 (4698, 7555)	201.98 (159.67, 254.61)	79.13 (60.37, 99.23)	83.68 (66.98, 103.23)	5.76 (-11.1, 25.3)	0.19 (0.11, 0.27)	0.15 (-0.07, 0.37)
Democratic People's Republic of Korea	11784 (8960, 15257)	28155 (21699, 35964)	138.93 (114.38, 170.38)	97.13 (74.76, 122.91)	96.57 (75.12, 122.93)	-0.58 (-10.91, 12.11)	-0.05 (-0.1, 0)	0.06 (-0.05, 0.17)
Democratic Republic of the Congo	11531 (7393, 18829)	27824 (18995, 38915)	141.3 (87.52, 201.31)	101.41 (64.49, 165.61)	104.42 (70.43, 145.61)	2.96 (-19.53, 29.3)	0.09 (0.04, 0.15)	0 (-0.11, 0.1)
Denmark	11759 (9487, 14789)	16963 (13877, 20897)	44.26 (32.08, 56.23)	137.76 (110.85, 172.83)	137.23 (111.86, 170.64)	-0.38 (-8.32, 7.5)	-0.05 (-0.17, 0.08)	-0.36 (-0.56, -0.16)
Djibouti	71 (46, 94)	313 (219, 414)	342.19 (243.45, 484.75)	76.71 (48.63, 101.31)	77.52 (53.58, 100.61)	1.06 (-20.4, 31.65)	0 (-0.13, 0.12)	-0.13 (-1.16, 0.91)
Dominica	82 (70, 95)	109 (92, 131)	33.29 (12.48, 57.81)	110.46 (93.45, 127.95)	119.33 (100.15, 142.62)	8.03 (-9.21, 28.05)	0.3 (0.2, 0.4)	0.28 (-1.52, 2.12)
Dominican Republic	2323 (1892, 2784)	7736 (6211, 9458)	233.08 (181.18, 300.87)	76.12 (62.67, 90.23)	90.95 (73.12, 111.08)	19.49 (0.76, 42.7)	0.65 (0.37, 0.94)	0.88 (0.67, 1.1)
Ecuador	2498 (2193, 2964)	9551 (7759, 11645)	282.29 (212.08, 367.34)	55.06 (48.21, 65.75)	72.69 (59.44, 88.27)	32.01 (6.53, 60.27)	1.01 (0.84, 1.18)	1.09 (0.89, 1.28)
Egypt	17440 (13078, 22470)	40893 (29899, 53997)	134.48 (102.09, 175.31)	78.24 (59.11, 98.86)	85.85 (62.32, 110.67)	9.71 (-5.96, 27.84)	0.3 (-0.03, 0.63)	0.34 (0.26, 0.42)
El Salvador	1844 (1517, 2195)	4685 (3712, 5708)	154.02 (115.16, 199.24)	67.23 (55.21, 79.86)	73.52 (58.16, 90.41)	9.35 (-7.5, 28.72)	0.32 (0.14, 0.5)	0.09 (-0.2, 0.38)
Equatorial Guinea	134 (92, 179)	429 (304, 581)	219.78 (122.46, 352.04)	87.52 (59.65, 117.35)	119.46 (85.19, 163.85)	36.49 (-8.05, 91.87)	1.08 (1.01, 1.14)	0.85 (-0.06, 1.77)

Eritrea	492 (320, 702)	1505 (1061, 1947)	206.15 (130.49, 306.75)	74.14 (48.35, 109.66)	85.11 (59.17, 111.05)	14.79 (-15.5, 55.01)	0.48 (0.4, 0.57)	0.08 (-0.37, 0.53)
Estonia	2388 (1826, 3121)	3911 (2963, 5111)	63.8 (46.66, 82.88)	118.26 (90.88, 154.42)	137.68 (103.3, 179.54)	16.42 (4.62, 28.89)	0.49 (0.36, 0.62)	0.48 (0.09, 0.87)
Eswatini	158 (122, 201)	332 (257, 425)	110.64 (72.94, 162.69)	68.4 (53.91, 86.51)	76.11 (59.09, 96.47)	11.27 (-8.93, 39.18)	0.36 (0.27, 0.45)	0.43 (-0.34, 1.2)
Ethiopia	9948 (6474, 13590)	21970 (15023, 27863)	120.84 (64.16, 202.09)	70.83 (44.27, 97.18)	67.64 (45.72, 85.46)	-4.5 (-28.79, 30.94)	-0.18 (-0.26, -0.09)	-0.62 (-0.73, -0.5)
Fiji	349 (274, 440)	815 (643, 1021)	133.5 (95.2, 180.56)	120.75 (96.28, 151.91)	133.13 (107.11, 164.82)	10.26 (-6.71, 30.29)	0.34 (0.27, 0.4)	0.43 (-0.08, 0.95)
Finland	9593 (7460, 12140)	16183 (12363, 20604)	68.7 (56.5, 83.6)	133.04 (104.08, 167.91)	120.88 (92.01, 154.01)	-9.14 (-15.19, -1.73)	-0.36 (-0.45, -0.27)	-0.39 (-0.6, -0.19)
France	121230 (96166, 152937)	179825 (141222, 226712)	48.33 (38.48, 57.86)	138.94 (108.97, 175.51)	116.24 (90.34, 148.61)	-16.34 (-21.42, -11.25)	-0.64 (-0.7, -0.57)	-0.68 (-0.75, -0.6)
Gabon	521 (370, 712)	999 (743, 1271)	91.7 (50.08, 140.63)	113.01 (79.53, 152.71)	120.62 (90.4, 152.56)	6.73 (-16.06, 35.43)	0.23 (0.17, 0.29)	-0.03 (-0.54, 0.49)
Gambia	189 (142, 245)	675 (540, 833)	257.58 (204.09, 331.97)	75.2 (57.17, 95.81)	89.47 (71.89, 109.22)	18.97 (1.31, 44.98)	0.65 (0.52, 0.79)	0.41 (-0.34, 1.18)
Georgia	7048 (5299, 9289)	9930 (7925, 12306)	40.88 (19.64, 64.67)	124.35 (94.41, 163.11)	155.57 (122.96, 193.37)	25.1 (2.96, 46.39)	0.85 (0.49, 1.2)	0.92 (0.69, 1.16)
Germany	207960 (159505, 280064)	333196 (273203, 414843)	60.22 (33.05, 81.6)	158.51 (120.8, 211.67)	155.89 (126.49, 196.4)	-1.65 (-17.82, 9.77)	-0.05 (-0.4, 0.3)	0.17 (-0.08, 0.43)
Ghana	3034 (2323, 3919)	9080 (7102, 11399)	199.29 (161.69, 244.51)	68.81 (54.3, 86.62)	76.96 (61.54, 94.77)	11.85 (-3.38, 30.19)	0.38 (0.34, 0.43)	0.29 (0.11, 0.48)
Greece	20076 (15558, 26909)	32485 (25371, 44510)	61.81 (49.07, 82.24)	132.94 (103.37, 178.5)	119.96 (92.52, 160.23)	-9.77 (-14.73, -2.9)	-0.38 (-0.46, -0.31)	-0.49 (-0.6, -0.37)

Greenland	58 (47, 70)	128 (103, 157)	119.89 (92.84, 149.47)	223.01 (183.07, 267.8)	214.86 (175.09, 264.43)	-3.65 (-15.2, 8.47)	-0.1 (-0.28, 0.08)	-0.32 (-2.02, 1.41)
Grenada	75 (62, 90)	94 (80, 116)	24.74 (5.71, 43.89)	93.5 (77.16, 111.95)	98.21 (83.59, 121.68)	5.04 (-10.1, 20.8)	0.15 (-0.27, 0.57)	0.01 (-1.64, 1.68)
Guam	77 (63, 95)	196 (154, 251)	154.08 (122.73, 186.22)	132.78 (109.68, 161.95)	105.84 (83.07, 136.07)	-20.29 (-30.55, -10.24)	-0.89 (-1.1, -0.69)	-0.51 (-1.67, 0.66)
Guatemala	1921 (1528, 2359)	6487 (5133, 8088)	237.63 (188.09, 294.17)	72.2 (57.32, 87.04)	67.39 (53.14, 83.25)	-6.66 (-19.47, 8.4)	-0.29 (-0.55, -0.04)	-0.43 (-0.67, -0.2)
Guinea	2025 (1493, 2595)	3687 (2870, 4581)	82.12 (54.38, 115.7)	75.77 (55.8, 96.19)	82.98 (65.41, 102.29)	9.52 (-9.12, 31.87)	0.34 (0.29, 0.39)	0.34 (0.06, 0.63)
Guinea-Bissau	229 (169, 294)	425 (329, 533)	85.94 (52.99, 126.29)	79.83 (60.07, 101.31)	87.98 (69.13, 109.87)	10.21 (-10.75, 36.49)	0.34 (0.31, 0.36)	0.31 (-0.46, 1.08)
Guyana	314 (267, 398)	558 (446, 701)	77.54 (46.98, 111.35)	100.58 (85.93, 125.68)	108.96 (88.07, 136.65)	8.33 (-9.59, 27.36)	0.19 (0.07, 0.31)	0.37 (-0.21, 0.95)
Haiti	2536 (1946, 3346)	5243 (3903, 7242)	106.7 (65.81, 164.19)	107.04 (79.8, 140.71)	98.37 (74.55, 135.09)	-8.1 (-26.6, 20.3)	-0.32 (-0.41, -0.24)	-0.13 (-0.34, 0.07)
Honduras	1186 (877, 1680)	4523 (3679, 5645)	281.38 (206.9, 360.24)	69.5 (49.9, 101.76)	92.63 (74.86, 116.14)	33.29 (5.55, 62.72)	0.92 (0.08, 1.77)	0.8 (0.48, 1.11)
Hungary	20819 (15777, 27082)	26679 (20367, 34890)	28.15 (18.78, 39.58)	144.59 (110.96, 186.34)	131.8 (100.04, 172.95)	-8.85 (-15.44, -1.61)	-0.33 (-0.42, -0.24)	-0.42 (-0.57, -0.28)
Iceland	438 (355, 552)	799 (638, 1006)	82.54 (69.25, 94.83)	147.76 (119.09, 187.48)	133.41 (105.29, 168.57)	-9.71 (-16.2, -3.72)	-0.34 (-0.63, -0.05)	-0.05 (-0.81, 0.72)
India	316554 (232128, 413070)	1024772 (786262, 1309013)	223.73 (184.78, 267.88)	97.36 (74.6, 125.5)	105.66 (81.67, 132.84)	8.52 (-6.02, 23.35)	0.29 (0.06, 0.51)	0.26 (0.19, 0.33)
Indonesia	74620 (54793, 98539)	192969 (143385, 249120)	158.6 (136.97, 184.99)	96.33 (72.41, 126.77)	113.21 (86.59, 143.72)	17.52 (6.82, 31.37)	0.56 (0.52, 0.6)	0.38 (0.35, 0.42)

Iran (Islamic Republic of)	13784 (10574, 17796)	47997 (37710, 60548)	248.22 (216.69, 301.74)	75.31 (57.33, 95.61)	75.3 (59.93, 94.58)	-0.01 (-10.41, 19.06)	-0.01 (-0.27, 0.25)	-0.1 (-0.18, -0.02)
Iraq	5745 (4386, 7901)	18169 (14107, 24797)	216.27 (167.27, 278.77)	83.87 (63.61, 116.26)	96.62 (75.38, 137.25)	15.2 (-1.91, 38.56)	0.45 (0.32, 0.58)	0.43 (0.32, 0.53)
Ireland	6213 (4987, 7854)	10588 (8349, 13245)	70.41 (54.37, 82.55)	152.66 (123.78, 192.91)	137.38 (107.66, 171.91)	-10.01 (-18.55, -4.09)	-0.34 (-0.39, -0.28)	-0.68 (-0.9, -0.46)
Israel	6545 (5282, 8310)	14140 (11109, 18197)	116.04 (101.9, 133.76)	137.75 (111.29, 173.54)	116.32 (90.74, 150.52)	-15.56 (-20.57, -9.03)	-0.62 (-0.79, -0.44)	-0.8 (-0.97, -0.62)
Italy	125086 (98191, 163261)	189787 (152217, 238078)	51.73 (37.7, 64.06)	140.1 (110.3, 182.78)	115.84 (91.37, 147.56)	-17.32 (-25.73, -11.78)	-0.65 (-0.73, -0.57)	-0.8 (-0.86, -0.73)
Jamaica	1418 (1166, 1724)	2991 (2386, 3853)	110.94 (75.42, 149.53)	78.18 (64.67, 94.84)	93.1 (74.03, 120.71)	19.07 (-1.25, 41.96)	0.7 (0.23, 1.18)	0.56 (0.24, 0.87)
Japan	107315 (87104, 134814)	211572 (169138, 263445)	97.15 (76.6, 119.42)	66.28 (54.13, 82.97)	53.15 (42.54, 66.74)	-19.81 (-25.18, -12.85)	-0.9 (-1.1, -0.71)	-0.99 (-1.11, -0.88)
Jordan	845 (666, 1068)	4033 (3169, 5063)	377.23 (315.98, 448.81)	88.73 (70.68, 110.64)	85.19 (68.51, 104.14)	-3.98 (-16.96, 10.88)	-0.16 (-0.34, 0.02)	-0.35 (-0.65, -0.05)
Kazakhstan	13396 (9640, 18386)	21272 (15655, 29564)	58.8 (37.08, 78.62)	119.72 (86.33, 165.61)	146.76 (110.39, 207.2)	22.59 (2.95, 40.41)	0.76 (0.66, 0.86)	0.26 (0.15, 0.37)
Kenya	3988 (2897, 5165)	12426 (9173, 15664)	211.6 (166.5, 285.54)	61.52 (43.95, 79.26)	78.47 (56.47, 99.88)	27.55 (7.59, 60.41)	0.94 (0.74, 1.14)	1.14 (0.98, 1.29)
Kiribati	39 (31, 49)	70 (54, 88)	78.23 (49.46, 113.97)	125.08 (98.8, 157.28)	124.71 (98.8, 154.5)	-0.29 (-14.08, 16.04)	-0.04 (-0.1, 0.02)	-0.09 (-2.08, 1.95)
Kuwait	306 (232, 402)	1352 (1020, 1792)	341.4 (304.34, 388.52)	66.55 (50.97, 85.8)	64.7 (49.47, 84.46)	-2.78 (-10.76, 6.69)	-0.21 (-0.65, 0.23)	-0.07 (-0.52, 0.39)
Kyrgyzstan	2798 (2083, 3698)	4256 (3177, 5591)	52.12 (42.13, 62.95)	97.02 (72.81, 127.74)	108.95 (82.81, 140.7)	12.29 (4.51, 20.07)	0.31 (0.01, 0.6)	0.36 (0.12, 0.61)

Lao People's Democratic Republic	1452 (1031, 1984)	3504 (2643, 4555)	141.33 (113.79, 172.94)	88.84 (65.14, 117.9)	100.43 (77.33, 127.89)	13.04 (-0.02, 27.3)	0.42 (0.4, 0.44)	0.36 (0.07, 0.64)
Latvia	4014 (3077, 5279)	5436 (4163, 6989)	35.41 (24.92, 47.18)	112.9 (86.81, 148.08)	126.88 (95.98, 164.14)	12.38 (4.07, 20.93)	0.36 (0.05, 0.67)	0.46 (0.14, 0.78)
Lebanon	1597 (1264, 2022)	4428 (3336, 5559)	177.29 (126.84, 235.94)	87.09 (69.39, 109.06)	86.47 (65.25, 108.85)	-0.72 (-19.08, 21.48)	-0.03 (-0.06, 0)	-0.05 (-0.35, 0.25)
Lesotho	517 (395, 672)	774 (591, 976)	49.7 (24.83, 86.04)	62.81 (47.86, 80.52)	79.68 (61.39, 99.6)	26.86 (5.14, 57.39)	0.88 (0.73, 1.04)	1.05 (0.55, 1.56)
Liberia	677 (516, 877)	1210 (941, 1528)	78.73 (54.43, 111.47)	77.59 (59.94, 98.67)	80.71 (63.04, 101.6)	4.02 (-11.26, 23.29)	0.08 (0.01, 0.15)	0.16 (-0.31, 0.64)
Libya	1066 (802, 1414)	3095 (2248, 3981)	190.21 (148.32, 246.62)	66.48 (49.76, 88.61)	70.76 (51.83, 91.33)	6.44 (-8.69, 28.02)	0.21 (0.12, 0.3)	0.24 (-0.06, 0.54)
Lithuania	5401 (4138, 6976)	8224 (6208, 10643)	52.26 (39.58, 67.83)	120.14 (92.36, 155.08)	133.1 (99.48, 174.78)	10.79 (1.86, 20.28)	0.29 (-0.03, 0.61)	0.35 (0.08, 0.61)
Luxembourg	838 (669, 1069)	1489 (1175, 1889)	77.68 (58.73, 93.97)	152.79 (122.52, 194.4)	140.2 (109.56, 179.75)	-8.24 (-18.47, -0.16)	-0.27 (-0.36, -0.18)	-0.19 (-0.76, 0.38)
Madagascar	3696 (2515, 4643)	7346 (5264, 9513)	98.76 (54.57, 154.04)	89.87 (60.46, 112.95)	93.69 (65.65, 120.29)	4.25 (-17.91, 31.8)	0.14 (0, 0.27)	-0.1 (-0.25, 0.06)
Malawi	1786 (1193, 2297)	3897 (2642, 4943)	118.15 (75.84, 177.36)	63.56 (41.82, 81.89)	70.07 (46.97, 87.9)	10.24 (-10.15, 40.01)	0.36 (0.2, 0.53)	0.21 (-0.04, 0.46)
Malaysia	7759 (5896, 10118)	25181 (19097, 32307)	224.53 (192.38, 263.78)	98.65 (76.21, 127.75)	109.7 (84.5, 139.98)	11.2 (-0.45, 25.88)	0.25 (-0.45, 0.95)	0.37 (0.26, 0.47)
Maldives	61 (44, 83)	250 (188, 327)	309.49 (243.98, 379.87)	97.1 (72.6, 126.66)	99.21 (75.87, 127.04)	2.17 (-13.99, 17.45)	0.04 (-0.04, 0.12)	-0.18 (-1.45, 1.1)
Mali	2259 (1659, 2922)	5353 (4173, 6727)	136.92 (103.92, 174.2)	77.51 (57.29, 98.72)	84.82 (66.2, 105.27)	9.43 (-6.33, 27.81)	0.32 (0.29, 0.35)	0.25 (0.01, 0.49)

Malta	539 (422, 705)	1131 (887, 1444)	109.85 (93.82, 125.42)	130.35 (102.67, 168.94)	114.9 (89.45, 147.87)	-11.86 (-18.82, -5.62)	-0.44 (-0.58, -0.3)	-0.2 (-0.89, 0.5)
Marshall Islands	19 (14, 24)	40 (30, 51)	112.74 (75.58, 158.36)	136.72 (106.55, 172.72)	147.09 (114.96, 187.12)	7.59 (-9.67, 29.27)	0.21 (0.12, 0.3)	0.38 (-2.02, 2.84)
Mauritania	663 (521, 826)	1410 (1110, 1757)	112.56 (82.09, 148.63)	83.92 (66.62, 102.29)	83.45 (66.01, 103.11)	-0.57 (-15.99, 17.03)	-0.05 (-0.1, 0)	-0.05 (-0.54, 0.44)
Mauritius	633 (489, 831)	1703 (1312, 2192)	168.96 (145.82, 195.21)	101.37 (79.56, 131.85)	103.71 (80.14, 133)	2.31 (-6.31, 11.89)	0.09 (0.01, 0.18)	0.01 (-0.45, 0.47)
Mexico	31834 (26982, 38692)	99070 (81768, 122854)	211.21 (180.85, 242.95)	90.27 (76.84, 110.44)	91.86 (75.82, 114.22)	1.76 (-8.17, 11.63)	0.08 (-0.06, 0.22)	0.06 (-0.03, 0.16)
Micronesia (Federated States of)	54 (41, 69)	86 (65, 110)	60.43 (25.5, 101.25)	140.53 (108.57, 179.01)	156.79 (122.38, 198.07)	11.57 (-10.34, 36.3)	0.38 (0.35, 0.41)	0.36 (-1.08, 1.83)
Monaco	103 (80, 129)	130 (102, 162)	26.71 (12.42, 41.73)	132.15 (102.9, 168.33)	122.34 (95.13, 155.06)	-7.42 (-17.78, 3.15)	-0.25 (-0.27, -0.24)	-0.33 (-2.68, 2.08)
Mongolia	1088 (833, 1400)	2208 (1649, 2886)	103.05 (78.92, 129.09)	122.55 (95.05, 157.13)	127.39 (98.98, 161.65)	3.96 (-7.2, 17.48)	0.15 (0.06, 0.24)	0.01 (-0.35, 0.37)
Montenegro	1287 (1078, 1563)	2377 (1955, 2959)	84.73 (60.69, 115.14)	223.28 (188.18, 270.88)	250.62 (205.82, 313.72)	12.24 (-2.5, 31.37)	0.51 (0.23, 0.78)	0.25 (-0.19, 0.7)
Morocco	8292 (6384, 10648)	22500 (17710, 28189)	171.33 (139.35, 207.95)	76.25 (59.27, 96.92)	89.32 (70.27, 110.18)	17.13 (3.1, 32.73)	0.54 (0.36, 0.72)	0.34 (0.22, 0.47)
Mozambique	2871 (1817, 3784)	6734 (4412, 9338)	134.55 (84.15, 208.1)	65.73 (40.22, 86.87)	84.39 (54.17, 116.32)	28.38 (-0.24, 65.46)	0.91 (0.85, 0.97)	1.01 (0.81, 1.2)
Myanmar	17053 (12525, 22550)	39276 (29923, 50422)	130.32 (106.66, 158.55)	90.84 (68.29, 117.48)	99.7 (76.99, 126.11)	9.75 (-1.32, 23.05)	0.35 (0.29, 0.4)	0.19 (0.1, 0.27)
Namibia	425 (330, 534)	959 (746, 1200)	125.7 (90.45, 164.75)	71.69 (55.95, 88.7)	80.95 (63.56, 100.37)	12.91 (-5.39, 31.96)	0.47 (0.37, 0.58)	0.14 (-0.37, 0.66)

Nauru	4 (3, 6)	5 (3, 6)	8.22 (-9.47, 31.45)	146.05 (117.46, 183.03)	150.32 (117.66, 190.06)	2.93 (-10.29, 20.43)	0.11 (0.04, 0.19)	0.11 (-5.72, 6.29)
Nepal	5971 (4206, 8264)	18597 (14007, 24134)	211.45 (167, 265.82)	81.83 (58.35, 112.01)	100.29 (75.42, 129.72)	22.56 (3.95, 45.95)	0.73 (0.67, 0.78)	0.49 (0.34, 0.64)
Netherlands	26486 (21745, 32397)	43581 (34852, 55605)	64.55 (52.44, 79.67)	129.44 (106.15, 158.6)	120.3 (95.51, 153.9)	-7.06 (-13.76, 1.47)	-0.27 (-0.33, -0.2)	-0.41 (-0.55, -0.28)
New Zealand	7120 (5552, 9285)	14563 (11633, 18207)	104.52 (87.96, 121.8)	183.26 (143.76, 237.07)	175.07 (139.28, 218.7)	-4.47 (-12.07, 3.02)	-0.16 (-0.23, -0.08)	-0.19 (-0.38, 0.01)
Nicaragua	942 (787, 1123)	3508 (2940, 4158)	272.56 (226.54, 333.72)	73.65 (61.7, 87.35)	99.89 (84.02, 117.79)	35.64 (18.83, 58.15)	1.17 (0.72, 1.63)	0.5 (0.18, 0.83)
Niger	1357 (961, 1829)	4125 (3042, 5355)	203.9 (169.75, 246.99)	72.69 (51.73, 97.42)	77.14 (56.98, 98.57)	6.12 (-7.81, 21.71)	0.22 (0.18, 0.26)	0.17 (-0.1, 0.45)
Nigeria	29462 (22758, 37915)	55781 (43338, 69220)	89.33 (43.4, 121.66)	89.28 (69.61, 114.86)	87.69 (68.73, 107.82)	-1.78 (-28.37, 15.91)	-0.08 (-0.12, -0.03)	-0.17 (-0.23, -0.1)
Niue	3 (2, 4)	3 (2, 4)	-6.73 (-20.44, 7.06)	128.64 (102.77, 158.94)	130.4 (103.33, 164.13)	1.37 (-13.38, 16.4)	0.04 (0.02, 0.05)	-0.02 (-6.9, 7.36)
North Macedonia	2517 (2028, 3142)	4438 (3517, 5601)	76.34 (58.44, 97.12)	160.46 (132.03, 195.9)	159.84 (129.27, 197.35)	-0.39 (-10.88, 12.92)	0 (-0.03, 0.04)	-0.11 (-0.43, 0.21)
Northern Mariana Islands	20 (16, 25)	64 (51, 78)	220.21 (166.43, 274.88)	145.29 (117.83, 179.81)	153.03 (125.88, 182.92)	5.33 (-9.28, 20)	0.13 (0.01, 0.24)	0.31 (-1.86, 2.53)
Norway	10599 (8625, 13128)	14446 (11281, 17668)	36.29 (21.65, 44.9)	145.51 (118.14, 180.62)	138.06 (108.02, 170.33)	-5.13 (-14.54, 0.35)	-0.19 (-0.32, -0.05)	-0.43 (-0.64, -0.23)
Oman	432 (324, 552)	1094 (871, 1367)	152.97 (104.82, 225.26)	112.66 (80.36, 142.8)	120.67 (95.12, 145.85)	7.1 (-15.13, 47.72)	0.18 (-0.18, 0.54)	-0.03 (-0.46, 0.39)
Pakistan	49558 (37034, 65078)	95095 (72530, 121183)	91.89 (68.37, 120.33)	99.63 (74.85, 130.29)	112.61 (87.07, 141.19)	13.02 (-1.2, 31.47)	0.42 (0.38, 0.45)	0.35 (0.3, 0.4)

Palau	9 (7, 12)	18 (14, 24)	104.62 (69.6, 141.69)	106.25 (80.98, 136.69)	104.56 (81.28, 132.36)	-1.59 (-17.24, 15.98)	-0.05 (-0.08, -0.03)	-0.06 (-3.28, 3.27)
Palestine	631 (484, 802)	1685 (1343, 2147)	166.87 (122.76, 237.06)	85.31 (65.53, 108.51)	92.92 (74.6, 117.47)	8.92 (-8.87, 39.38)	0.39 (0.23, 0.55)	0.24 (-0.14, 0.62)
Panama	928 (770, 1129)	3109 (2443, 3933)	235.08 (188.92, 289.3)	67.54 (55.84, 81.86)	73.5 (57.6, 93.12)	8.83 (-5.8, 26.08)	0.31 (0.19, 0.43)	0.26 (-0.1, 0.62)
Papua New Guinea	1441 (1035, 1966)	4112 (3014, 5473)	185.44 (146.69, 229.61)	101.68 (73.06, 137.63)	111.18 (83.7, 145.8)	9.34 (-4.62, 25.21)	0.31 (0.28, 0.34)	0.35 (0.12, 0.58)
Paraguay	1740 (1408, 2131)	4959 (3936, 6207)	185.04 (138.34, 243.95)	86.49 (70.09, 105.91)	95.45 (75.89, 119.73)	10.36 (-7.73, 32.64)	0.42 (0.31, 0.53)	0.26 (0.02, 0.5)
Peru	6788 (5690, 8006)	17548 (13728, 21939)	158.53 (102.14, 234.01)	65.03 (54.71, 76.15)	54.39 (42.48, 68.2)	-16.37 (-34.23, 8.34)	-0.4 (-0.77, -0.03)	-0.61 (-0.75, -0.47)
Philippines	19787 (14080, 27094)	62919 (47210, 82568)	217.98 (193.68, 253.46)	85.55 (63.21, 115.27)	97.53 (74.38, 126.25)	14 (6.23, 25.44)	0.47 (0.39, 0.55)	0.78 (0.68, 0.88)
Poland	58212 (44786, 76332)	106704 (83133, 135429)	83.3 (61.84, 100.48)	138.33 (107.6, 179.68)	146.05 (113.54, 186.37)	5.58 (-6.84, 14.53)	0.22 (0.07, 0.37)	0.31 (0.2, 0.42)
Portugal	18260 (14048, 23812)	29239 (22636, 38168)	60.13 (49.16, 76.6)	134.59 (104.83, 173.58)	110.87 (84.5, 145.72)	-17.62 (-22.56, -11.45)	-0.71 (-0.76, -0.66)	-0.86 (-1, -0.72)
Puerto Rico	2586 (2154, 3142)	5941 (4678, 7426)	129.74 (98.58, 165.19)	74.69 (62.18, 90.14)	73.03 (57.64, 92.08)	-2.23 (-15.63, 12.65)	-0.08 (-0.2, 0.04)	-0.11 (-0.39, 0.18)
Qatar	122 (93, 149)	753 (565, 975)	516.91 (356.71, 723.8)	194.99 (142.12, 238.95)	178.86 (142.32, 228.96)	-8.27 (-33.19, 34.84)	-0.16 (-0.57, 0.25)	-0.79 (-1.57, -0.01)
Republic of Korea	16138 (12664, 20214)	49455 (39535, 61932)	206.46 (173.31, 243.37)	65.94 (53.47, 80.34)	56.82 (45.46, 70.8)	-13.84 (-23.44, -4.1)	-0.52 (-0.61, -0.43)	-0.6 (-0.68, -0.52)
Republic of Moldova	4665 (3438, 6231)	7005 (5189, 9336)	50.16 (40.69, 62.15)	119.7 (91.07, 156.81)	120.03 (89.33, 159.45)	0.28 (-6.68, 8.82)	-0.03 (-0.45, 0.4)	-0.11 (-0.34, 0.12)

Romania	30787 (22783, 40697)	45680 (34325, 59763)	48.37 (35.88, 63.32)	118.37 (89.69, 153.47)	116.9 (87.37, 153.71)	-1.24 (-8.84, 7.25)	-0.25 (-0.48, -0.02)	-0.45 (-0.56, -0.34)
Russian Federation	193458 (144204, 256288)	312583 (237566, 408735)	61.58 (51.84, 72.4)	115.6 (87.61, 151.51)	130.9 (99.5, 170.9)	13.23 (6.07, 20.32)	0.46 (0.29, 0.63)	0.38 (0.33, 0.43)
Rwanda	1902 (1259, 2591)	3486 (2448, 4373)	83.31 (37.44, 137.88)	87.11 (57.99, 119.21)	79.35 (54.68, 99.08)	-8.91 (-30.7, 16.3)	-0.36 (-0.5, -0.22)	-0.9 (-1.16, -0.65)
Saint Kitts and Nevis	56 (47, 67)	75 (63, 94)	34.95 (15.91, 56.02)	161.84 (138.29, 194.4)	146.73 (125.35, 182.39)	-9.34 (-21.49, 3.62)	-0.4 (-1.05, 0.26)	-0.67 (-2.6, 1.3)
Saint Lucia	89 (75, 106)	222 (185, 270)	149.1 (119.34, 178.31)	117.82 (100.79, 140.18)	110.01 (91.89, 133.88)	-6.63 (-17.73, 4.31)	-0.27 (-0.61, 0.08)	-0.51 (-1.75, 0.75)
Saint Vincent and the Grenadines	69 (56, 81)	132 (111, 164)	93.13 (64.43, 131.77)	104.09 (85.51, 120.95)	108.12 (91.09, 133.85)	3.87 (-11.43, 24.75)	0.14 (-0.08, 0.37)	0.12 (-1.38, 1.65)
Samoa	99 (77, 124)	170 (133, 213)	71.75 (46.48, 99.43)	130.54 (102.3, 161.39)	129.58 (101.89, 162.28)	-0.74 (-14.6, 15.08)	-0.05 (-0.07, -0.02)	0.01 (-0.94, 0.98)
San Marino	44 (33, 56)	89 (67, 115)	104.21 (69.94, 147.99)	130.91 (101.09, 168.61)	123.64 (91.42, 160.96)	-5.55 (-20.65, 12.77)	-0.19 (-0.23, -0.15)	-0.26 (-2.97, 2.52)
Sao Tome and Principe	39 (30, 49)	72 (58, 90)	87.15 (59.4, 122.7)	74.33 (57.42, 92.05)	90.16 (72.25, 111.18)	21.29 (2.16, 45.99)	0.7 (0.6, 0.8)	0.53 (-1.48, 2.58)
Saudi Arabia	3593 (2628, 4668)	9816 (7548, 12763)	173.23 (111.68, 275.51)	81.78 (59.92, 105.68)	85.11 (66.98, 105.14)	4.07 (-20, 43.04)	0.16 (0.08, 0.24)	0.09 (-0.05, 0.23)
Senegal	1852 (1396, 2370)	4825 (3767, 6079)	160.44 (127, 202.18)	74.54 (55.64, 93.7)	82.46 (64.54, 103.12)	10.62 (-4.33, 28.46)	0.34 (0.23, 0.44)	0.31 (0.05, 0.58)
Serbia	13491 (10493, 17623)	21640 (16702, 28024)	60.4 (43.6, 80.7)	131.11 (103.89, 169.12)	139.63 (108.59, 179.45)	6.5 (-4.48, 19.58)	0.21 (0.05, 0.37)	-0.11 (-0.25, 0.03)
Seychelles	59 (46, 75)	106 (80, 137)	80.21 (64.33, 101.29)	105.83 (82.07, 134.34)	108.85 (82.2, 139.96)	2.86 (-6.4, 16.01)	0.07 (-0.12, 0.27)	-0.03 (-1.54, 1.5)

Sierra Leone	1186 (888, 1556)	2194 (1671, 2771)	85.07 (61.13, 115.8)	73.94 (56.17, 95.1)	79.39 (61.49, 100.19)	7.38 (-7.43, 26.14)	0.27 (0.21, 0.33)	0.28 (-0.08, 0.64)
Singapore	1241 (979, 1588)	3935 (3015, 5113)	217.2 (180.63, 252.68)	64.24 (51.78, 80.19)	51.94 (40.06, 67.04)	-19.14 (-27.72, -10.61)	-0.79 (-0.95, -0.63)	-0.71 (-0.96, -0.45)
Slovakia	8893 (6854, 11371)	13586 (10485, 17529)	52.76 (37.25, 70.04)	152.24 (118.25, 193.29)	146.25 (112.91, 187.67)	-3.93 (-13.78, 6.72)	-0.04 (-0.21, 0.14)	0.02 (-0.16, 0.2)
Slovenia	3227 (2493, 4163)	5800 (4422, 7500)	79.73 (52.85, 109.44)	134.37 (104.22, 173)	124.96 (94.67, 162.2)	-7 (-20.95, 7.63)	-0.2 (-0.32, -0.08)	-0.29 (-0.62, 0.04)
Solomon Islands	125 (90, 171)	315 (237, 425)	152.7 (110, 203.62)	115.5 (84.48, 152.24)	127.94 (98.35, 163.44)	10.77 (-6.07, 28.74)	0.36 (0.26, 0.46)	0.39 (-0.85, 1.65)
Somalia	1244 (755, 1708)	2878 (1777, 3966)	131.4 (83.66, 198.63)	69.57 (40.6, 96.64)	64.46 (37.68, 89.04)	-7.34 (-26.73, 19.05)	-0.27 (-0.33, -0.21)	-0.34 (-0.63, -0.06)
South Africa	13337 (10745, 16758)	30445 (24786, 37352)	128.27 (114.93, 142.78)	72.78 (58.79, 90.96)	80.28 (66.26, 97.2)	10.3 (3.15, 17.86)	0.38 (0.19, 0.57)	-0.04 (-0.12, 0.03)
South Sudan	1414 (910, 1902)	1883 (1285, 2528)	33.13 (4.46, 71)	73.82 (46.69, 98.45)	64.93 (43.6, 87.68)	-12.04 (-30.1, 12.15)	-0.47 (-0.52, -0.42)	-0.58 (-0.88, -0.27)
Spain	79337 (62669, 101914)	135054 (108181, 168601)	70.23 (58.89, 84.06)	145.16 (115.33, 185.04)	123.88 (97.64, 157.71)	-14.66 (-19.53, -8.7)	-0.62 (-0.8, -0.44)	-0.62 (-0.77, -0.48)
Sri Lanka	7699 (5640, 10231)	21409 (16192, 28046)	178.07 (145.47, 217.38)	87.6 (65.84, 113.82)	94.03 (71.76, 122.08)	7.33 (-5.92, 22.92)	0.29 (0.2, 0.38)	0.29 (0.15, 0.42)
Sudan	5412 (4113, 7122)	11806 (8970, 15215)	118.15 (87.43, 153.99)	70.88 (54.38, 92.57)	77.82 (59.82, 99.82)	9.79 (-5.82, 27.32)	0.31 (0.28, 0.34)	0.19 (0.05, 0.34)
Suriname	202 (172, 236)	495 (408, 597)	144.58 (113.19, 178.69)	85.15 (72.82, 99.11)	89.87 (73.93, 107.84)	5.55 (-7.81, 20.28)	-0.07 (-0.66, 0.53)	0.09 (-0.65, 0.83)
Sweden	27123 (20734, 34935)	44218 (29996, 56073)	63.03 (36.63, 80.61)	168.71 (129.82, 217.59)	188.07 (132.83, 241.02)	11.47 (-3.61, 21.42)	0.46 (0.31, 0.6)	0.35 (0.19, 0.5)

Switzerland	11148 (8699, 14264)	17616 (13769, 22507)	58.02 (46.97, 71.88)	102.09 (79.11, 131.09)	92.69 (71.5, 120.19)	-9.21 (-15.3, -1.43)	-0.24 (-0.39, -0.1)	-0.13 (-0.3, 0.05)
Syrian Arab Republic	3544 (2750, 4462)	8477 (6460, 10816)	139.21 (101.52, 193.71)	83.34 (65.11, 103.79)	90.57 (69.49, 113.06)	8.67 (-8.05, 33.56)	0.3 (0.2, 0.4)	0.04 (-0.14, 0.23)
Taiwan (Province of China)	11304 (8452, 14892)	35193 (26881, 45210)	211.32 (174.34, 263.57)	87.52 (67.54, 112.49)	87.09 (66.54, 111.88)	-0.48 (-10.64, 12.76)	-0.03 (-0.11, 0.05)	-0.13 (-0.25, -0.02)
Tajikistan	3093 (2204, 4059)	4837 (3696, 6222)	56.35 (32.45, 91.59)	115.86 (83.16, 152.2)	147.07 (117.62, 182.69)	26.93 (10.1, 56.86)	0.92 (0.57, 1.28)	0.51 (0.14, 0.88)
Thailand	27539 (20949, 35355)	94884 (71374, 123673)	244.54 (202.72, 291.81)	98.48 (76.76, 124.7)	95.56 (72.27, 124.94)	-2.97 (-15.62, 10.61)	-0.16 (-0.37, 0.04)	-0.15 (-0.22, -0.09)
Timor-Leste	167 (119, 229)	654 (485, 869)	291.07 (247.85, 347.36)	80.75 (58.71, 109.99)	96.73 (72.81, 126.94)	19.78 (6.93, 37.48)	0.62 (0.59, 0.65)	0.68 (-0.05, 1.42)
Togo	705 (548, 888)	2125 (1640, 2657)	201.32 (159.19, 254.66)	80.21 (62.79, 99.53)	86.61 (68.31, 106.93)	7.98 (-9.23, 30.08)	0.26 (0.23, 0.29)	0.21 (-0.16, 0.59)
Tokelau	2 (1, 2)	2 (1, 2)	-3.4 (-19.86, 16.11)	124.41 (95.37, 160.06)	127.86 (101.81, 159.67)	2.78 (-14.76, 22.83)	0.11 (0.06, 0.16)	0.13 (-8.75, 9.88)
Tonga	52 (40, 67)	90 (70, 115)	71.44 (48.73, 99.7)	108.46 (84.47, 138.38)	117.17 (91.79, 150.11)	8.04 (-5.92, 24.05)	0.31 (0.12, 0.5)	0.22 (-1.1, 1.56)
Trinidad and Tobago	570 (462, 689)	1461 (1115, 1833)	156.42 (111.49, 205.68)	78.11 (63.87, 94.03)	82.78 (63.32, 104)	5.98 (-13.23, 26.21)	0.16 (-0.08, 0.4)	0.19 (-0.31, 0.69)
Tunisia	3059 (2404, 3925)	9335 (7127, 11851)	205.19 (159.25, 266.09)	76.74 (61.13, 97.05)	82.77 (63.74, 104.79)	7.86 (-8.51, 30.15)	0.26 (0.21, 0.3)	0.25 (0.04, 0.45)
Turkey	25981 (20569, 32275)	61729 (47405, 79021)	137.59 (105.26, 179)	85.52 (67.83, 106.82)	74.82 (57.84, 95.3)	-12.51 (-25.03, 4.03)	-0.48 (-0.75, -0.21)	-0.85 (-1.05, -0.66)
Turkmenistan	1751 (1311, 2318)	4315 (3289, 5581)	146.46 (121.48, 174.42)	107.08 (81.44, 140.39)	130.65 (100.58, 167.74)	22.01 (9.48, 35.8)	0.66 (0.33, 1)	0.56 (0.29, 0.83)

Tuvalu	8 (6, 10)	12 (10, 15)	59.34 (29.37, 94.5)	134.54 (103.49, 174.24)	135.04 (106.88, 169.56)	0.37 (-18.45, 22.55)	0 (-0.03, 0.03)	-0.02 (-3.75, 3.85)
Uganda	3242 (1929, 4326)	7937 (4967, 10423)	144.83 (97.88, 208.62)	64.55 (37.58, 86.22)	74.91 (46.54, 98.31)	16.04 (-6.1, 45.08)	0.48 (0.35, 0.61)	0.28 (0.11, 0.46)
Ukraine	92139 (71064, 120570)	121591 (94110, 156580)	31.96 (22.06, 44.06)	135.43 (104.84, 174.63)	155.92 (121.12, 200.19)	15.13 (6.17, 24.85)	0.45 (0.24, 0.67)	0.54 (0.47, 0.6)
United Arab Emirates	248 (179, 340)	2214 (1470, 3300)	793.33 (579.75, 1053.29)	94.05 (66.93, 127.04)	90.02 (60.43, 129.24)	-4.29 (-25.72, 20.54)	-0.29 (-0.5, -0.09)	-0.06 (-0.87, 0.76)
United Kingdom	137280 (108760, 175693)	199715 (158674, 248134)	45.48 (35.88, 54.19)	146.83 (116.07, 187.33)	148.14 (116.72, 184.99)	0.89 (-5.19, 6.15)	0.05 (-0.03, 0.13)	0.21 (0.1, 0.32)
United Republic of Tanzania	6915 (4781, 8642)	16457 (11439, 20869)	137.99 (97.48, 187.2)	83.56 (56.03, 105.3)	84.74 (58.4, 106.72)	1.41 (-15.61, 20.86)	0.03 (-0.07, 0.12)	-0.2 (-0.32, -0.07)
United States of America	466823 (360325, 611267)	955312 (754899, 1198324)	104.64 (90.02, 117.65)	140.01 (107.86, 182.87)	162.34 (127.34, 203.97)	15.96 (8.11, 23.19)	0.58 (0.41, 0.74)	0.94 (0.87, 1)
United States Virgin Islands	67 (56, 80)	196 (166, 231)	192.32 (152.95, 241.41)	97.97 (82.68, 115.42)	115.41 (98.6, 135.98)	17.79 (2.32, 37.16)	0.66 (0.43, 0.89)	0.64 (-0.87, 2.18)
Uruguay	3618 (2754, 4446)	5733 (4515, 7038)	58.46 (48.72, 69.39)	93.18 (71.29, 113.87)	93.87 (73.79, 116.24)	0.74 (-5.1, 7.6)	-0.06 (-0.28, 0.17)	-0.04 (-0.35, 0.28)
Uzbekistan	9607 (6688, 13442)	20563 (15839, 26570)	114.03 (68.31, 153.98)	93.72 (65.62, 131.65)	164.01 (134.73, 200.8)	75 (37.66, 120.36)	2.05 (1.9, 2.21)	1.67 (1.56, 1.79)
Vanuatu	61 (44, 81)	186 (141, 245)	207.42 (161.56, 267.44)	112.2 (82.96, 150.11)	125.63 (95.86, 161.32)	11.97 (-3.76, 32.36)	0.32 (0.22, 0.43)	0.48 (-0.79, 1.77)
Venezuela (Bolivarian Republic of)	6190 (5121, 7594)	21986 (17333, 27579)	255.18 (206.92, 316.89)	75.55 (62.46, 92)	81.03 (64.06, 101.17)	7.26 (-7.39, 25.23)	0.22 (-0.04, 0.48)	0 (-0.13, 0.14)
Viet Nam	35170 (26536, 45695)	91404 (69115, 116525)	159.89 (122.68, 202.03)	99.04 (75.74, 126.87)	114.08 (86.71, 144.54)	15.18 (-2.25, 34.57)	0.48 (0.45, 0.52)	0.53 (0.47, 0.58)

Yemen	2670 (1965, 3591)	7951 (5992, 10322)	197.77 (154.68, 255.23)	73.32 (54.02, 96.44)	77.67 (59.98, 99.56)	5.93 (-8.81, 26.49)	0.22 (0.19, 0.24)	0.24 (0.04, 0.43)
Zambia	1497 (1075, 1915)	4873 (3513, 6011)	225.61 (151.19, 327.82)	71.5 (50.04, 92.16)	99.95 (72.15, 122.67)	39.79 (6.61, 83.35)	1.22 (1.07, 1.37)	1.06 (0.81, 1.32)
Zimbabwe	2007 (1523, 2545)	3555 (2690, 4478)	77.11 (56.18, 102.21)	68.1 (51.51, 84.9)	71.95 (54.32, 89.59)	5.64 (-7.86, 22.23)	0.18 (0.07, 0.28)	0.25 (0, 0.5)
