# Supplementary material for: Differences in global, regional, and national time trends in disability-adjusted life years for atrial fibrillation and flutter, 1990–2019: an age-period-cohort analysis from the 2019 global burden of disease study
Source: Front Cardiovasc Med. 2024 Aug 29;11:1401722. doi: 10.3389/fcvm.2024.1401722 (PMC11390633; doi:10.3389/fcvm.2024.1401722)
Supplement: Supplementary file 2 [file Table2.pdf]

Table S2. Tests for atrial fibrillation and flutter DALY for both sexes across socio-demographic index quintiles and 21 regions, 1990-2019

location	sex	All Age Deviations = 0	All Cohort Deviations = 0	All Cohort RR = 1	All Gradient Shifts = CAT	All Higher-Order Age Deviations = 0	All Higher-Order Cohort Deviations = 0	All Higher-Order Period Deviations = 0	All Local Drifts = Net Drift	All Period Deviations = 0	All Period RR = 1	NetDrift = 0	THETAa = 0	THETAc = 0	THETAp = 0
Global	Both	< 0.001	< 0.001	< 0.001	< 0.001	< 0.001	< 0.001	< 0.001	< 0.001	< 0.001	< 0.001	< 0.001	< 0.001	< 0.001	< 0.001
Global	Female	< 0.001	< 0.001	< 0.001	< 0.001	< 0.001	< 0.001	< 0.001	< 0.001	< 0.001	< 0.001	0.777	< 0.001	0.607	< 0.001
Global	Male	< 0.001	< 0.001	< 0.001	< 0.001	< 0.001	< 0.001	< 0.001	< 0.001	< 0.001	< 0.001	< 0.001	< 0.001	< 0.001	< 0.001
High SDI	Both	< 0.001	< 0.001	< 0.001	< 0.001	< 0.001	< 0.001	< 0.001	< 0.001	< 0.001	< 0.001	0.016	< 0.001	0.232	< 0.001
High SDI	Female	< 0.001	< 0.001	< 0.001	< 0.001	< 0.001	< 0.001	< 0.001	< 0.001	< 0.001	< 0.001	0.083	< 0.001	0.559	< 0.001
High SDI	Male	< 0.001	< 0.001	< 0.001	< 0.001	< 0.001	< 0.001	< 0.001	< 0.001	< 0.001	< 0.001	0.379	< 0.001	0.149	< 0.001
High-middle SDI	Both	< 0.001	< 0.001	< 0.001	< 0.001	< 0.001	< 0.001	0.013	< 0.001	< 0.001	< 0.001	< 0.001	< 0.001	0.014	0.001
High-middle SDI	Female	< 0.001	< 0.001	< 0.001	0.007	< 0.001	< 0.001	< 0.001	< 0.001	< 0.001	< 0.001	< 0.001	< 0.001	0.506	0.003

High-middle SDI	Male	< 0.001	< 0.001	< 0.001	< 0.001	< 0.001	< 0.001	0.488	< 0.001	0.382	< 0.001	< 0.001	< 0.001	< 0.001	0.222
Middle SDI	Both	< 0.001	0.002	< 0.001	0.021	< 0.001	0.002	< 0.001	< 0.001	< 0.001	< 0.001	< 0.001	< 0.001	0.05	< 0.001
Middle SDI	Female	< 0.001	0.009	< 0.001	0.004	< 0.001	0.025	0.103	0.003	< 0.001	< 0.001	0.021	< 0.001	0.007	< 0.001
Middle SDI	Male	< 0.001	0.006	< 0.001	0.069	< 0.001	0.051	< 0.001	0.003	< 0.001	< 0.001	< 0.001	< 0.001	0.082	< 0.001
Low-middle SDI	Both	< 0.001	< 0.001	< 0.001	< 0.001	< 0.001	0.003	0.366	< 0.001	< 0.001	< 0.001	< 0.001	< 0.001	0.005	< 0.001
Low-middle SDI	Female	< 0.001	< 0.001	< 0.001	< 0.001	< 0.001	0.14	0.006	< 0.001	< 0.001	< 0.001	< 0.001	< 0.001	0.018	< 0.001
Low-middle SDI	Male	< 0.001	< 0.001	< 0.001	< 0.001	< 0.001	< 0.001	0.482	< 0.001	< 0.001	< 0.001	< 0.001	< 0.001	0.005	< 0.001
Low SDI	Both	< 0.001	< 0.001	< 0.001	< 0.001	< 0.001	0.27	0.819	< 0.001	< 0.001	< 0.001	< 0.001	< 0.001	< 0.001	< 0.001
Low SDI	Female	< 0.001	< 0.001	< 0.001	< 0.001	< 0.001	0.536	0.954	< 0.001	< 0.001	< 0.001	< 0.001	< 0.001	< 0.001	< 0.001
Low SDI	Male	< 0.001	< 0.001	< 0.001	< 0.001	< 0.001	0.023	0.484	< 0.001	< 0.001	< 0.001	< 0.001	< 0.001	< 0.001	< 0.001
High-income	Both	< 0.001	< 0.001	< 0.001	0.163	< 0.001	0.242	< 0.001	< 0.001	< 0.001	< 0.001	< 0.001	< 0.001	0.024	< 0.001

Asia Pacific															
High-income Asia Pacific	Female	< 0.001	< 0.001	< 0.001	< 0.001	< 0.001	0.007	< 0.001	< 0.001	< 0.001	< 0.001	< 0.001	< 0.001	0.347	< 0.001
High-income Asia Pacific	Male	< 0.001	< 0.001	< 0.001	< 0.001	< 0.001	< 0.001	< 0.001	< 0.001	< 0.001	< 0.001	< 0.001	< 0.001	< 0.001	< 0.001
High-income North America	Both	< 0.001	< 0.001	< 0.001	< 0.001	< 0.001	< 0.001	< 0.001	< 0.001	< 0.001	< 0.001	< 0.001	< 0.001	< 0.001	< 0.001
High-income North America	Female	< 0.001	< 0.001	< 0.001	< 0.001	< 0.001	< 0.001	< 0.001	< 0.001	< 0.001	< 0.001	< 0.001	< 0.001	0.042	< 0.001
High-income North America	Male	< 0.001	< 0.001	< 0.001	< 0.001	< 0.001	< 0.001	< 0.001	< 0.001	< 0.001	< 0.001	< 0.001	< 0.001	< 0.001	< 0.001
Western Europe	Both	< 0.001	< 0.001	< 0.001	< 0.001	< 0.001	0.009	0.15	< 0.001	0.016	< 0.001	< 0.001	< 0.001	0.013	0.005
Western Europe	Female	< 0.001	< 0.001	< 0.001	< 0.001	< 0.001	< 0.001	0.037	< 0.001	< 0.001	< 0.001	< 0.001	< 0.001	0.042	< 0.001

Western Europe	Male	< 0.001	< 0.001	< 0.001	< 0.001	< 0.001	0.012	0.428	< 0.001	0.27	< 0.001	< 0.001	< 0.001	0.023	0.089
Australasia	Both	< 0.001	0.093	< 0.001	0.285	< 0.001	0.071	0.191	0.038	0.019	< 0.001	< 0.001	< 0.001	0.466	0.024
Australasia	Female	< 0.001	0.192	< 0.001	0.156	< 0.001	0.605	0.124	0.092	0.028	< 0.001	< 0.001	< 0.001	0.978	0.06
Australasia	Male	< 0.001	0.214	< 0.001	0.293	< 0.001	0.171	0.929	0.102	0.094	0.002	< 0.001	< 0.001	0.757	0.009
Andean Latin America	Both	< 0.001	0.119	0.026	0.073	< 0.001	0.987	< 0.001	0.083	< 0.001	< 0.001	0.596	< 0.001	0.008	< 0.001
Andean Latin America	Female	< 0.001	0.257	0.294	0.8	< 0.001	0.212	0.061	0.437	0.115	0.175	0.572	< 0.001	0.522	0.608
Andean Latin America	Male	< 0.001	< 0.001	< 0.001	< 0.001	< 0.001	0.406	0.003	< 0.001	< 0.001	< 0.001	0.963	< 0.001	< 0.001	< 0.001
Tropical Latin America	Both	< 0.001	0.08	< 0.001	0.498	< 0.001	0.238	< 0.001	0.03	< 0.001	< 0.001	< 0.001	< 0.001	0.935	0.211
Tropical Latin America	Female	< 0.001	0.034	< 0.001	0.346	< 0.001	0.27	< 0.001	0.014	< 0.001	< 0.001	< 0.001	< 0.001	0.758	0.094
Tropical Latin America	Male	< 0.001	0.051	< 0.001	0.208	< 0.001	0.054	< 0.001	0.02	< 0.001	< 0.001	< 0.001	< 0.001	0.462	0.824

Central Latin America	Both	< 0.001	0.037	0.052	0.021	< 0.001	0.339	0.002	0.015	0.002	0.002	0.276	< 0.001	0.185	0.937
Central Latin America	Female	< 0.001	0.891	0.148	0.722	< 0.001	0.952	0.002	0.756	0.002	0.002	0.061	< 0.001	0.587	0.847
Central Latin America	Male	< 0.001	0.007	< 0.001	0.001	< 0.001	0.218	0.454	0.004	0.605	< 0.001	< 0.001	< 0.001	0.104	0.932
Southern Latin America	Both	< 0.001	< 0.001	< 0.001	< 0.001	< 0.001	< 0.001	0.092	< 0.001	< 0.001	< 0.001	0.005	< 0.001	0.008	< 0.001
Southern Latin America	Female	< 0.001	< 0.001	< 0.001	0.016	< 0.001	< 0.001	0.113	< 0.001	< 0.001	< 0.001	0.538	< 0.001	0.062	< 0.001
Southern Latin America	Male	< 0.001	0.046	< 0.001	0.128	< 0.001	0.856	0.691	0.082	0.205	< 0.001	< 0.001	< 0.001	0.045	0.048
Caribbean	Both	< 0.001	0.956	< 0.001	0.996	< 0.001	0.95	0.286	0.933	< 0.001	< 0.001	< 0.001	< 0.001	0.625	< 0.001
Caribbean	Female	< 0.001	0.98	0.468	0.99	< 0.001	0.989	0.39	0.97	< 0.001	< 0.001	0.153	< 0.001	0.684	< 0.001
Caribbean	Male	< 0.001	0.977	< 0.001	0.996	< 0.001	0.964	0.499	0.945	0.046	< 0.001	< 0.001	< 0.001	0.604	0.011
Central Europe	Both	< 0.001	< 0.001	< 0.001	0.01	< 0.001	< 0.001	0.001	< 0.001	< 0.001	< 0.001	0.156	< 0.001	0.039	< 0.001

Central Europe	Female	< 0.001	< 0.001	< 0.001	0.008	< 0.001	0.014	0.041	0.003	< 0.001	< 0.001	0.007	< 0.001	0.028	< 0.001
Central Europe	Male	< 0.001	0.002	< 0.001	0.01	< 0.001	0.002	0.002	0.003	< 0.001	< 0.001	< 0.001	< 0.001	0.036	< 0.001
Eastern Europe	Both	< 0.001	< 0.001	< 0.001	< 0.001	< 0.001	< 0.001	< 0.001	< 0.001	< 0.001	< 0.001	< 0.001	< 0.001	0.072	< 0.001
Eastern Europe	Female	< 0.001	< 0.001	< 0.001	0.014	< 0.001	< 0.001	< 0.001	< 0.001	< 0.001	< 0.001	< 0.001	< 0.001	0.167	< 0.001
Eastern Europe	Male	< 0.001	< 0.001	< 0.001	< 0.001	< 0.001	< 0.001	0.005	< 0.001	< 0.001	< 0.001	< 0.001	< 0.001	< 0.001	< 0.001
Central Asia	Both	< 0.001	< 0.001	< 0.001	< 0.001	< 0.001	0.159	0.988	< 0.001	0.985	< 0.001	< 0.001	< 0.001	< 0.001	0.606
Central Asia	Female	< 0.001	< 0.001	< 0.001	< 0.001	< 0.001	0.26	0.995	< 0.001	0.986	< 0.001	< 0.001	< 0.001	0.006	0.59
Central Asia	Male	< 0.001	< 0.001	< 0.001	< 0.001	< 0.001	0.994	0.842	< 0.001	0.768	< 0.001	< 0.001	< 0.001	< 0.001	0.267
North Africa and Middle East	Both	< 0.001	0.053	0.048	0.001	< 0.001	0.211	0.637	0.022	0.015	0.029	0.645	< 0.001	0.121	0.004
North Africa and Middle East	Female	< 0.001	0.043	0.059	0.001	< 0.001	0.334	0.57	0.016	0.167	0.24	0.987	< 0.001	0.177	0.026
North Africa and	Male	< 0.001	0.579	0.249	0.153	< 0.001	0.581	< 0.001	0.464	< 0.001	< 0.001	0.192	< 0.001	0.149	0.004

Middle East															
South Asia	Both	< 0.001	0.02	< 0.001	0.005	< 0.001	0.014	0.018	0.024	0.018	< 0.001	< 0.001	< 0.001	0.131	0.462
South Asia	Female	< 0.001	0.139	< 0.001	0.573	< 0.001	0.248	< 0.001	0.144	< 0.001	< 0.001	< 0.001	< 0.001	0.769	0.512
South Asia	Male	< 0.001	< 0.001	< 0.001	< 0.001	< 0.001	< 0.001	0.403	< 0.001	0.148	< 0.001	< 0.001	< 0.001	0.01	0.079
Southeast Asia	Both	< 0.001	< 0.001	< 0.001	< 0.001	< 0.001	< 0.001	0.662	< 0.001	0.006	< 0.001	< 0.001	< 0.001	0.022	< 0.001
Southeast Asia	Female	< 0.001	< 0.001	< 0.001	< 0.001	< 0.001	< 0.001	0.613	< 0.001	< 0.001	< 0.001	< 0.001	< 0.001	0.067	< 0.001
Southeast Asia	Male	< 0.001	< 0.001	< 0.001	< 0.001	< 0.001	< 0.001	0.513	< 0.001	0.641	< 0.001	< 0.001	< 0.001	< 0.001	0.498
East Asia	Both	< 0.001	< 0.001	< 0.001	< 0.001	< 0.001	< 0.001	< 0.001	< 0.001	< 0.001	< 0.001	0.047	< 0.001	0.389	< 0.001
East Asia	Female	< 0.001	< 0.001	< 0.001	< 0.001	< 0.001	< 0.001	0.018	< 0.001	< 0.001	< 0.001	< 0.001	< 0.001	0.162	< 0.001
East Asia	Male	< 0.001	0.199	< 0.001	0.114	< 0.001	0.658	< 0.001	0.125	< 0.001	< 0.001	< 0.001	< 0.001	0.22	< 0.001
Oceania	Both	< 0.001	1	0.589	0.867	< 0.001	1	0.979	0.999	0.988	0.192	0.007	< 0.001	0.149	0.787
Oceania	Female	< 0.001	1	0.934	0.811	< 0.001	1	0.981	0.999	0.988	0.56	0.052	< 0.001	0.136	0.772
Oceania	Male	< 0.001	1	0.993	1	< 0.001	1	0.994	1	0.999	0.571	0.053	< 0.001	0.941	0.954

Western Sub-Saharan Africa	Both	< 0.001	0.983	0.355	0.65	< 0.001	0.997	0.099	0.944	0.177	0.015	0.009	< 0.001	0.943	0.867
Western Sub-Saharan Africa	Female	< 0.001	0.999	1	0.994	< 0.001	0.999	0.246	0.998	0.253	0.299	0.617	< 0.001	0.642	0.427
Western Sub-Saharan Africa	Male	< 0.001	0.325	< 0.001	0.059	< 0.001	0.806	0.556	0.168	0.718	< 0.001	< 0.001	< 0.001	0.384	0.81
Eastern Sub-Saharan Africa	Both	< 0.001	< 0.001	< 0.001	< 0.001	< 0.001	0.717	0.004	< 0.001	< 0.001	< 0.001	0.692	< 0.001	< 0.001	< 0.001
Eastern Sub-Saharan Africa	Female	< 0.001	< 0.001	< 0.001	< 0.001	< 0.001	0.998	0.017	< 0.001	< 0.001	< 0.001	0.283	< 0.001	< 0.001	< 0.001
Eastern Sub-Saharan Africa	Male	< 0.001	< 0.001	< 0.001	< 0.001	< 0.001	0.878	0.223	< 0.001	< 0.001	< 0.001	0.995	< 0.001	< 0.001	< 0.001
Central Sub-	Both	< 0.001	0.047	0.04	0.059	< 0.001	0.96	0.413	0.016	< 0.001	< 0.001	0.234	< 0.001	0.025	< 0.001

Saharan Africa															
Central Sub-Saharan Africa	Female	< 0.001	0.842	0.047	0.556	< 0.001	1	0.944	0.678	< 0.001	< 0.001	0.013	< 0.001	0.074	< 0.001
Central Sub-Saharan Africa	Male	< 0.001	0.989	0.298	0.726	< 0.001	0.999	0.195	0.955	< 0.001	< 0.001	0.08	< 0.001	0.498	< 0.001
Southern Sub-Saharan Africa	Both	< 0.001	< 0.001	< 0.001	< 0.001	< 0.001	< 0.001	0.016	< 0.001	< 0.001	< 0.001	0.634	< 0.001	< 0.001	< 0.001
Southern Sub-Saharan Africa	Female	< 0.001	< 0.001	< 0.001	< 0.001	< 0.001	< 0.001	0.337	< 0.001	0.022	0.008	0.186	< 0.001	< 0.001	0.003
Southern Sub-Saharan Africa	Male	< 0.001	< 0.001	< 0.001	< 0.001	< 0.001	0.521	0.015	< 0.001	< 0.001	< 0.001	0.007	< 0.001	< 0.001	< 0.001
Afghanistan	Both	< 0.001	0.999	0.081	0.928	< 0.001	1	0.947	0.991	0.91	0.074	0.004	< 0.001	0.251	0.396
Afghanistan	Female	< 0.001	1	0.211	0.944	< 0.001	1	0.971	0.997	0.991	0.208	0.01	< 0.001	0.332	0.793

Afghanistan	Male	< 0.001	1	1	0.997	< 0.001	1	0.99	1	0.933	0.899	0.443	< 0.001	0.653	0.382
Albania	Both	< 0.001	1	1	0.951	< 0.001	1	0.973	1	0.937	0.926	0.526	< 0.001	0.649	0.426
Albania	Female	< 0.001	1	1	0.997	< 0.001	1	0.988	1	0.997	0.996	0.641	< 0.001	0.84	0.861
Albania	Male	< 0.001	1	1	1	< 0.001	1	0.976	1	0.97	0.965	0.569	< 0.001	0.906	0.58
Algeria	Both	< 0.001	0.947	0.805	0.667	< 0.001	0.927	0.777	0.881	0.894	0.949	0.818	< 0.001	0.623	0.86
Algeria	Female	< 0.001	0.981	0.987	0.508	< 0.001	0.984	0.722	0.944	0.807	0.842	0.547	< 0.001	0.564	0.446
Algeria	Male	< 0.001	0.835	0.046	0.98	< 0.001	0.941	0.819	0.712	0.92	0.621	0.118	< 0.001	0.967	0.969
American Samoa	Both	0.99	1	1	1	1	1	1	1	1	1	0.876	0.142	0.944	0.991
American Samoa	Female	0.997	1	1	1	0.998	1	1	1	1	1	0.875	0.349	0.942	0.949
American Samoa	Male	0.991	1	1	1	1	1	1	1	1	1	0.936	0.27	0.979	0.969
Andorra	Both	0.083	1	1	1	1	1	0.996	1	0.99	0.991	0.573	0.007	0.841	0.671
Andorra	Female	0.565	1	1	1	1	1	0.998	1	0.993	0.998	0.872	0.201	0.921	0.699
Andorra	Male	0.353	1	1	1	0.998	1	0.999	1	0.999	0.998	0.635	0.017	0.945	0.841
Angola	Both	< 0.001	0.521	0.004	0.351	< 0.001	0.999	0.973	0.314	0.632	< 0.001	< 0.001	< 0.001	0.061	0.124

Angola	Female	< 0.001	0.967	0.038	0.732	< 0.001	1	0.993	0.892	0.946	0.011	< 0.001	< 0.001	0.122	0.403
Angola	Male	< 0.001	0.995	0.93	0.887	< 0.001	1	0.962	0.975	0.804	0.247	0.044	< 0.001	0.37	0.249
Antigua and Barbuda	Both	0.99	1	1	0.995	0.998	1	0.997	1	0.997	0.999	0.923	0.208	0.704	0.733
Antigua and Barbuda	Female	0.996	1	1	1	1	1	0.998	1	1	0.999	0.757	0.364	0.838	0.962
Antigua and Barbuda	Male	1	1	1	1	1	1	0.972	1	0.985	0.995	0.808	0.389	0.782	0.668
Argentina	Both	< 0.001	< 0.001	< 0.001	0.026	< 0.001	0.013	< 0.001	0.058	< 0.001	< 0.001	0.437	< 0.001	0.021	0.049
Argentina	Female	< 0.001	< 0.001	< 0.001	0.042	< 0.001	< 0.001	< 0.001	0.005	< 0.001	< 0.001	0.942	< 0.001	0.06	< 0.001
Argentina	Male	< 0.001	0.073	< 0.001	0.46	< 0.001	0.77	0.114	0.151	0.201	0.145	0.165	< 0.001	0.089	0.871
Armenia	Both	< 0.001	0.741	0.001	0.314	< 0.001	1	0.486	0.574	0.477	0.033	0.005	< 0.001	0.269	0.209
Armenia	Female	< 0.001	0.896	0.1	0.363	< 0.001	1	0.612	0.777	0.616	0.351	0.112	< 0.001	0.434	0.265
Armenia	Male	< 0.001	1	0.699	0.975	< 0.001	1	0.892	1	0.934	0.311	0.028	< 0.001	0.544	0.57

Australia	Both	< 0.001	0.319	< 0.001	0.838	< 0.001	0.29	0.289	0.179	0.059	< 0.001	< 0.001	< 0.001	0.353	0.05
Australia	Female	< 0.001	0.455	< 0.001	0.778	< 0.001	0.519	0.211	0.283	0.078	< 0.001	< 0.001	< 0.001	0.759	0.103
Australia	Male	< 0.001	0.775	< 0.001	0.741	< 0.001	0.725	0.919	0.604	0.146	0.001	< 0.001	< 0.001	0.726	0.017
Austria	Both	< 0.001	< 0.001	< 0.001	< 0.001	< 0.001	< 0.001	< 0.001	< 0.001	< 0.001	< 0.001	< 0.001	< 0.001	0.817	< 0.001
Austria	Female	< 0.001	0.075	< 0.001	0.818	< 0.001	0.118	0.045	0.152	< 0.001	< 0.001	< 0.001	< 0.001	0.533	< 0.001
Austria	Male	< 0.001	0.133	< 0.001	0.227	< 0.001	0.456	0.037	0.086	< 0.001	< 0.001	< 0.001	< 0.001	0.91	< 0.001
Azerbaijan	Both	< 0.001	< 0.001	< 0.001	< 0.001	< 0.001	0.002	0.055	< 0.001	0.057	< 0.001	< 0.001	< 0.001	< 0.001	0.097
Azerbaijan	Female	< 0.001	< 0.001	< 0.001	< 0.001	< 0.001	0.043	0.121	< 0.001	0.104	< 0.001	< 0.001	< 0.001	0.02	0.098
Azerbaijan	Male	< 0.001	< 0.001	< 0.001	< 0.001	< 0.001	0.713	0.489	< 0.001	0.543	< 0.001	< 0.001	< 0.001	< 0.001	0.274
Bahamas	Both	0.176	1	1	1	0.616	1	0.965	1	0.982	0.991	0.65	0.001	0.938	0.694
Bahamas	Female	0.483	1	1	1	0.881	1	0.974	1	0.987	0.995	0.71	0.024	0.943	0.726
Bahamas	Male	0.883	1	1	1	0.952	1	0.992	1	0.997	0.999	0.89	0.043	0.808	0.774
Bahrain	Both	< 0.001	0.864	< 0.001	0.727	0.095	1	0.157	0.716	0.236	0.003	< 0.001	< 0.001	0.226	0.613
Bahrain	Female	0.053	0.999	< 0.001	0.93	0.629	1	0.25	0.994	0.351	0.002	< 0.001	0.002	0.333	0.658

Bahrain	Male	0.121	1	0.984	0.983	0.44	1	0.741	0.999	0.857	0.725	0.201	0.017	0.601	0.836
Bangladesh	Both	< 0.001	< 0.001	< 0.001	< 0.001	< 0.001	< 0.001	< 0.001	< 0.001	< 0.001	< 0.001	< 0.001	< 0.001	< 0.001	0.018
Bangladesh	Female	< 0.001	< 0.001	< 0.001	< 0.001	< 0.001	0.002	< 0.001	< 0.001	< 0.001	< 0.001	< 0.001	< 0.001	< 0.001	0.064
Bangladesh	Male	< 0.001	< 0.001	< 0.001	< 0.001	< 0.001	< 0.001	< 0.001	< 0.001	< 0.001	< 0.001	< 0.001	< 0.001	< 0.001	0.068
Barbados	Both	0.612	1	1	0.998	0.842	1	0.995	1	0.999	1	0.857	0.036	0.681	0.873
Barbados	Female	0.737	1	1	1	0.967	1	0.991	1	0.998	0.995	0.591	0.151	0.832	0.897
Barbados	Male	0.965	1	1	1	0.966	1	0.973	1	0.988	0.993	0.65	0.132	0.816	0.784
Belarus	Both	< 0.001	0.892	< 0.001	0.337	< 0.001	1	0.96	0.754	0.341	< 0.001	< 0.001	< 0.001	0.305	0.038
Belarus	Female	< 0.001	0.846	< 0.001	0.284	< 0.001	1	0.874	0.703	0.77	0.006	< 0.001	< 0.001	0.532	0.266
Belarus	Male	< 0.001	0.991	< 0.001	0.601	< 0.001	1	0.959	0.96	0.684	< 0.001	< 0.001	< 0.001	0.268	0.178
Belgium	Both	< 0.001	< 0.001	< 0.001	0.368	< 0.001	< 0.001	0.073	< 0.001	< 0.001	< 0.001	< 0.001	< 0.001	0.371	< 0.001
Belgium	Female	< 0.001	< 0.001	< 0.001	0.998	< 0.001	< 0.001	0.357	< 0.001	0.089	0.066	0.076	< 0.001	0.732	0.042
Belgium	Male	< 0.001	< 0.001	< 0.001	0.372	< 0.001	< 0.001	0.283	< 0.001	< 0.001	< 0.001	< 0.001	< 0.001	0.397	< 0.001
Belize	Both	0.725	1	1	1	0.992	1	0.935	1	0.855	0.911	0.466	0.013	0.96	0.277
Belize	Female	0.976	1	1	1	1	1	0.997	1	0.966	0.989	0.777	0.113	0.912	0.453

Belize	Male	0.965	1	1	1	0.999	1	0.92	1	0.926	0.948	0.467	0.065	0.871	0.431
Benin	Both	< 0.001	0.999	0.772	0.767	< 0.001	1	0.989	0.991	0.972	0.688	0.087	< 0.001	0.513	0.564
Benin	Female	< 0.001	1	0.998	0.961	< 0.001	1	0.99	1	0.995	0.975	0.399	< 0.001	0.694	0.802
Benin	Male	< 0.001	1	0.982	0.923	0.004	1	0.998	1	0.979	0.778	0.124	< 0.001	0.544	0.535
Bermuda	Both	0.994	1	1	1	0.999	1	0.998	1	0.998	0.976	0.387	0.247	0.919	0.769
Bermuda	Female	1	1	1	1	1	1	0.987	1	0.991	0.978	0.45	0.514	0.947	0.739
Bermuda	Male	1	1	1	1	1	1	0.999	1	1	0.999	0.682	0.36	0.894	0.889
Bhutan	Both	< 0.001	1	0.996	0.978	0.949	1	1	1	0.999	0.919	0.284	< 0.001	0.495	0.764
Bhutan	Female	0.025	1	0.999	0.988	0.988	1	1	1	1	0.974	0.419	0.001	0.586	0.807
Bhutan	Male	0.005	1	1	1	0.998	1	1	1	1	0.99	0.514	< 0.001	0.709	0.841
Bolivia (Plurinational State of)	Both	< 0.001	0.034	< 0.001	0.008	< 0.001	1	0.978	0.011	0.633	0.174	0.077	< 0.001	< 0.001	0.16
Bolivia (Plurinational State of)	Female	< 0.001	0.318	< 0.001	0.087	< 0.001	1	0.989	0.165	0.998	0.364	0.03	< 0.001	0.018	0.892

Bolivia (Plurinational State of)	Male	< 0.001	0.899	0.689	0.379	< 0.001	1	0.953	0.757	0.304	0.371	0.88	< 0.001	0.02	0.053
Bosnia and Herzegovina	Both	< 0.001	0.016	< 0.001	0.002	< 0.001	0.778	0.066	0.01	0.036	0.011	0.019	< 0.001	0.077	0.144
Bosnia and Herzegovina	Female	< 0.001	0.782	0.341	0.216	< 0.001	0.986	0.382	0.665	0.352	0.351	0.234	< 0.001	0.512	0.312
Bosnia and Herzegovina	Male	< 0.001	0.068	0.022	0.006	< 0.001	0.824	0.216	0.025	0.21	0.102	0.049	< 0.001	0.085	0.351
Botswana	Both	< 0.001	1	1	1	0.012	1	0.723	1	0.61	0.717	0.852	< 0.001	0.646	0.183
Botswana	Female	0.025	1	1	1	0.396	1	0.971	1	0.978	0.993	0.773	< 0.001	0.98	0.619
Botswana	Male	0.004	1	1	0.999	0.337	1	0.695	1	0.521	0.638	0.827	< 0.001	0.581	0.133
Brazil	Both	< 0.001	0.15	< 0.001	0.586	< 0.001	0.401	< 0.001	0.063	< 0.001	< 0.001	< 0.001	< 0.001	0.817	0.182
Brazil	Female	< 0.001	0.058	< 0.001	0.324	< 0.001	0.395	< 0.001	0.026	< 0.001	< 0.001	< 0.001	< 0.001	0.682	0.074

Brazil	Male	< 0.001	0.124	< 0.001	0.363	< 0.001	0.135	< 0.001	0.057	< 0.001	< 0.001	< 0.001	< 0.001	0.556	0.792
Brunei Darussalam	Both	0.204	1	1	0.99	0.591	1	0.998	1	0.975	0.99	0.71	0.012	0.611	0.501
Brunei Darussalam	Female	0.764	1	1	1	0.964	1	0.994	1	0.998	1	0.909	0.047	0.864	0.794
Brunei Darussalam	Male	0.54	1	1	0.999	0.674	1	0.988	1	0.859	0.932	0.861	0.212	0.856	0.256
Bulgaria	Both	< 0.001	< 0.001	< 0.001	< 0.001	< 0.001	0.285	0.092	< 0.001	0.037	< 0.001	< 0.001	< 0.001	0.331	0.054
Bulgaria	Female	< 0.001	0.487	0.263	0.231	< 0.001	0.777	0.186	0.425	0.261	0.369	0.731	< 0.001	0.701	0.417
Bulgaria	Male	< 0.001	0.054	< 0.001	0.02	< 0.001	0.191	0.11	0.018	0.074	< 0.001	< 0.001	< 0.001	0.885	0.145
Burkina Faso	Both	< 0.001	0.133	< 0.001	0.523	< 0.001	0.997	0.097	0.062	0.149	< 0.001	< 0.001	< 0.001	0.09	0.841
Burkina Faso	Female	< 0.001	0.389	< 0.001	0.749	< 0.001	0.998	0.179	0.229	0.255	< 0.001	< 0.001	< 0.001	0.147	0.812
Burkina Faso	Male	< 0.001	0.997	< 0.001	0.99	< 0.001	1	0.588	0.984	0.715	0.001	< 0.001	< 0.001	0.454	0.848
Burundi	Both	< 0.001	0.992	< 0.001	0.7	< 0.001	1	0.462	0.962	0.037	< 0.001	< 0.001	< 0.001	0.243	0.006

Burundi	Female	< 0.001	1	0.003	0.931	< 0.001	1	0.53	0.999	0.183	< 0.001	< 0.001	< 0.001	0.369	0.045
Burundi	Male	< 0.001	0.999	0.004	0.906	0.009	1	0.875	0.992	0.3	0.001	< 0.001	< 0.001	0.4	0.04
Cabo Verde	Both	0.01	1	0.826	0.892	0.775	1	0.723	0.999	0.828	0.743	0.34	0.009	0.516	0.835
Cabo Verde	Female	0.137	1	0.969	0.957	0.888	1	0.961	1	0.981	0.973	0.571	0.12	0.615	0.811
Cabo Verde	Male	0.522	1	1	0.99	0.997	1	0.686	1	0.824	0.83	0.515	0.031	0.615	0.988
Cambodia	Both	< 0.001	0.911	< 0.001	0.449	< 0.001	1	0.999	0.782	0.999	< 0.001	< 0.001	< 0.001	0.166	0.861
Cambodia	Female	< 0.001	0.983	0.08	0.68	< 0.001	1	0.997	0.936	1	0.051	0.001	< 0.001	0.357	0.992
Cambodia	Male	< 0.001	1	0.227	0.915	< 0.001	1	0.999	0.999	0.999	0.047	0.001	< 0.001	0.298	0.805
Cameroon	Both	< 0.001	1	0.003	0.968	< 0.001	1	0.808	1	0.909	0.001	< 0.001	< 0.001	0.813	0.972
Cameroon	Female	< 0.001	1	0.862	0.976	< 0.001	1	0.914	1	0.912	0.367	0.063	< 0.001	0.732	0.411
Cameroon	Male	< 0.001	1	0.034	0.997	< 0.001	1	0.921	1	0.873	0.005	< 0.001	< 0.001	0.88	0.483
Canada	Both	< 0.001	0.976	< 0.001	0.708	< 0.001	1	< 0.001	0.951	< 0.001	< 0.001	< 0.001	< 0.001	0.282	0.027
Canada	Female	< 0.001	1	< 0.001	0.976	< 0.001	1	0.005	0.998	0.005	< 0.001	0.004	< 0.001	0.558	0.062

Canada	Male	< 0.001	0.078	< 0.001	0.057	< 0.001	1	< 0.001	0.031	< 0.001	< 0.001	< 0.001	< 0.001	0.032	0.031
Central African Republic	Both	< 0.001	1	1	1	< 0.001	1	0.992	1	0.994	0.997	0.762	< 0.001	0.746	0.733
Central African Republic	Female	< 0.001	1	1	1	0.003	1	0.997	1	0.98	0.993	0.912	< 0.001	0.76	0.541
Central African Republic	Male	< 0.001	1	1	1	0.097	1	0.997	1	1	0.999	0.698	< 0.001	0.927	0.938
Chad	Both	< 0.001	1	0.753	0.999	< 0.001	1	0.987	1	0.972	0.382	0.023	< 0.001	0.852	0.577
Chad	Female	< 0.001	1	0.999	1	< 0.001	1	0.981	1	0.996	0.876	0.2	< 0.001	0.862	0.951
Chad	Male	< 0.001	1	0.968	0.994	< 0.001	1	0.996	1	0.977	0.636	0.069	< 0.001	0.766	0.536
Chile	Both	< 0.001	0.824	0.047	0.777	< 0.001	0.814	< 0.001	0.655	< 0.001	< 0.001	0.01	< 0.001	0.647	< 0.001
Chile	Female	< 0.001	0.993	0.994	0.99	< 0.001	0.992	0.059	0.982	0.001	0.002	0.922	< 0.001	0.92	< 0.001
Chile	Male	< 0.001	0.981	0.034	0.637	< 0.001	0.99	0.029	0.93	< 0.001	< 0.001	< 0.001	< 0.001	0.411	< 0.001
China	Both	< 0.001	< 0.001	< 0.001	< 0.001	< 0.001	< 0.001	< 0.001	< 0.001	< 0.001	< 0.001	0.058	< 0.001	0.441	< 0.001

China	Female	< 0.001	< 0.001	< 0.001	< 0.001	< 0.001	< 0.001	0.015	< 0.001	< 0.001	< 0.001	< 0.001	< 0.001	0.19	< 0.001
China	Male	< 0.001	0.176	< 0.001	0.101	< 0.001	0.607	< 0.001	0.112	< 0.001	< 0.001	< 0.001	< 0.001	0.2	< 0.001
Colombia	Both	< 0.001	< 0.001	< 0.001	0.02	< 0.001	0.001	0.026	0.003	0.046	0.042	0.264	< 0.001	0.392	0.414
Colombia	Female	< 0.001	< 0.001	< 0.001	0.062	< 0.001	< 0.001	0.298	< 0.001	0.411	0.522	0.533	< 0.001	0.475	0.512
Colombia	Male	< 0.001	0.959	0.949	0.511	< 0.001	1	0.04	0.889	0.073	0.024	0.063	< 0.001	0.093	0.54
Comoros	Both	0.043	1	1	0.999	0.736	1	0.992	1	0.932	0.954	0.504	< 0.001	0.752	0.408
Comoros	Female	0.232	1	1	1	0.806	1	0.998	1	0.983	0.992	0.642	0.01	0.804	0.572
Comoros	Male	0.724	1	1	1	0.998	1	0.996	1	0.977	0.987	0.619	0.012	0.866	0.544
Congo	Both	< 0.001	1	0.997	0.955	< 0.001	1	0.667	0.997	0.39	0.468	0.336	< 0.001	0.425	0.131
Congo	Female	< 0.001	1	1	0.996	< 0.001	1	0.791	1	0.859	0.933	0.972	< 0.001	0.693	0.618
Congo	Male	< 0.001	1	0.989	0.989	0.056	1	0.843	0.999	0.318	0.374	0.282	< 0.001	0.561	0.064
Cook Islands	Both	1	1	1	1	1	1	1	1	1	1	0.936	0.403	0.949	0.87
Cook Islands	Female	1	1	1	1	1	1	1	1	1	1	0.946	0.695	0.881	0.87

Cook Islands	Male	1	1	1	1	1	1	1	1	1	1	0.979	0.446	0.953	0.956
Costa Rica	Both	< 0.001	0.999	1	0.999	< 0.001	0.999	0.192	1	0.27	0.341	0.619	< 0.001	0.944	0.87
Costa Rica	Female	< 0.001	1	1	1	0.065	1	0.211	1	0.276	0.397	0.688	< 0.001	0.949	0.786
Costa Rica	Male	< 0.001	1	1	1	0.005	1	0.822	1	0.917	0.758	0.234	< 0.001	0.902	0.993
Croatia	Both	< 0.001	0.731	< 0.001	0.587	< 0.001	0.683	0.255	0.535	< 0.001	< 0.001	0.033	< 0.001	0.555	< 0.001
Croatia	Female	< 0.001	0.514	< 0.001	0.145	< 0.001	0.867	0.824	0.329	< 0.001	< 0.001	0.001	< 0.001	0.52	< 0.001
Croatia	Male	< 0.001	0.991	0.995	0.769	< 0.001	0.987	0.301	0.963	< 0.001	< 0.001	0.762	< 0.001	0.467	< 0.001
Cuba	Both	< 0.001	0.039	0.053	0.172	< 0.001	0.028	0.156	0.041	< 0.001	< 0.001	0.289	< 0.001	0.081	< 0.001
Cuba	Female	< 0.001	0.277	0.267	0.305	< 0.001	0.238	0.462	0.304	< 0.001	0.001	0.127	< 0.001	0.149	< 0.001
Cuba	Male	< 0.001	0.807	0.787	0.814	< 0.001	0.752	0.363	0.715	0.004	0.007	0.92	< 0.001	0.282	0.001
Cyprus	Both	< 0.001	0.755	< 0.001	0.983	< 0.001	0.704	0.756	0.85	0.457	0.002	< 0.001	< 0.001	0.868	0.088
Cyprus	Female	< 0.001	0.948	< 0.001	1	0.003	0.925	0.828	0.992	0.916	0.288	0.02	< 0.001	0.882	0.918
Cyprus	Male	< 0.001	0.995	0.001	0.991	< 0.001	0.992	0.941	0.993	0.194	0.015	0.014	< 0.001	0.967	0.015

Czechia	Both	< 0.001	0.767	< 0.001	0.941	< 0.001	0.778	< 0.001	0.779	< 0.001	< 0.001	< 0.001	< 0.001	0.467	< 0.001
Czechia	Female	< 0.001	0.643	< 0.001	0.955	< 0.001	0.88	0.031	0.683	< 0.001	< 0.001	0.638	< 0.001	0.413	0.002
Czechia	Male	< 0.001	0.987	< 0.001	0.961	< 0.001	0.985	< 0.001	0.951	< 0.001	< 0.001	< 0.001	< 0.001	0.541	< 0.001
C 么 te d'Ivoire	Both	< 0.001	1	0.997	0.924	< 0.001	1	0.985	0.999	0.997	0.836	0.174	< 0.001	0.843	0.967
C 么 te d'Ivoire	Female	< 0.001	1	1	0.996	< 0.001	1	0.974	1	0.985	0.85	0.262	< 0.001	0.862	0.637
C 么 te d'Ivoire	Male	< 0.001	1	1	0.978	< 0.001	1	0.97	1	0.965	0.946	0.378	< 0.001	0.848	0.518
Democratic People's Republic of Korea	Both	< 0.001	0.173	0.194	0.956	< 0.001	0.826	0.615	0.074	0.003	0.006	0.285	< 0.001	0.269	< 0.001
Democratic People's Republic of Korea	Female	< 0.001	0.104	0.053	0.959	< 0.001	0.714	0.661	0.04	0.004	0.01	0.609	< 0.001	0.34	< 0.001
Democratic People's Republic of Korea	Male	< 0.001	1	1	0.998	< 0.001	1	0.947	1	0.919	0.704	0.122	< 0.001	0.907	0.525
Democratic Republic	Both	< 0.001	0.872	0.907	0.73	< 0.001	1	0.469	0.723	< 0.001	< 0.001	0.94	< 0.001	0.228	< 0.001

of the Congo															
Democratic Republic of the Congo	Female	< 0.001	0.999	0.777	0.966	< 0.001	1	0.95	0.995	< 0.001	< 0.001	0.17	< 0.001	0.293	< 0.001
Democratic Republic of the Congo	Male	< 0.001	1	0.131	0.971	< 0.001	1	0.207	1	< 0.001	< 0.001	0.007	< 0.001	0.97	< 0.001
Denmark	Both	< 0.001	< 0.001	< 0.001	< 0.001	< 0.001	0.097	0.647	< 0.001	0.002	< 0.001	< 0.001	< 0.001	0.018	< 0.001
Denmark	Female	< 0.001	< 0.001	< 0.001	< 0.001	< 0.001	0.928	0.825	< 0.001	0.008	< 0.001	0.02	< 0.001	0.213	< 0.001
Denmark	Male	< 0.001	< 0.001	< 0.001	< 0.001	< 0.001	0.112	0.843	< 0.001	0.373	0.015	0.004	< 0.001	0.041	0.055
Djibouti	Both	0.134	1	1	1	0.8	1	0.997	1	0.986	0.996	0.81	< 0.001	0.725	0.574
Djibouti	Female	0.627	1	1	1	0.94	1	0.999	1	0.989	0.997	0.765	0.012	0.703	0.584
Djibouti	Male	0.657	1	1	1	0.992	1	0.999	1	0.999	1	0.97	0.022	0.882	0.786
Dominica	Both	0.974	1	1	1	0.995	1	0.998	1	0.998	0.999	0.76	0.252	0.843	0.758
Dominica	Female	0.995	1	1	1	0.999	1	0.999	1	0.999	1	0.923	0.57	0.865	0.84
Dominica	Male	1	1	1	1	1	1	0.999	1	1	1	0.909	0.431	0.923	0.872

Dominican Republic	Both	< 0.001	< 0.001	< 0.001	0.003	< 0.001	0.057	0.189	< 0.001	0.004	< 0.001	< 0.001	< 0.001	0.031	< 0.001
Dominican Republic	Female	< 0.001	< 0.001	< 0.001	0.005	< 0.001	0.002	0.043	< 0.001	< 0.001	< 0.001	< 0.001	< 0.001	0.069	< 0.001
Dominican Republic	Male	< 0.001	0.967	< 0.001	0.475	< 0.001	0.984	0.89	0.899	0.87	< 0.001	< 0.001	< 0.001	0.24	0.409
Ecuador	Both	< 0.001	< 0.001	< 0.001	< 0.001	< 0.001	< 0.001	< 0.001	< 0.001	< 0.001	< 0.001	< 0.001	0.787	< 0.001	< 0.001
Ecuador	Female	< 0.001	< 0.001	< 0.001	< 0.001	< 0.001	0.149	< 0.001	< 0.001	< 0.001	< 0.001	< 0.001	0.004	0.009	< 0.001
Ecuador	Male	< 0.001	< 0.001	< 0.001	< 0.001	< 0.001	< 0.001	< 0.001	< 0.001	< 0.001	< 0.001	< 0.001	0.06	< 0.001	< 0.001
Egypt	Both	< 0.001	0.352	< 0.001	0.782	< 0.001	0.46	0.049	0.274	0.008	< 0.001	< 0.001	< 0.001	0.149	0.017
Egypt	Female	< 0.001	< 0.001	< 0.001	< 0.001	< 0.001	0.024	0.379	< 0.001	0.537	< 0.001	< 0.001	< 0.001	< 0.001	0.721
Egypt	Male	< 0.001	0.36	< 0.001	0.599	< 0.001	0.333	0.057	0.365	0.035	< 0.001	< 0.001	< 0.001	0.997	0.072
El Salvador	Both	< 0.001	0.902	0.695	0.517	< 0.001	0.972	0.341	0.763	0.478	0.553	0.559	< 0.001	0.691	0.909
El Salvador	Female	< 0.001	0.95	0.901	0.612	< 0.001	0.988	0.901	0.856	0.567	0.687	0.969	< 0.001	0.639	0.183

El Salvador	Male	< 0.001	1	0.998	0.991	< 0.001	1	0.206	1	0.206	0.248	0.283	< 0.001	0.949	0.193
Equatorial Guinea	Both	0.004	1	0.886	0.934	0.74	1	0.985	0.996	0.961	0.44	0.067	< 0.001	0.3	0.447
Equatorial Guinea	Female	0.055	1	0.871	0.988	0.831	1	0.976	1	0.995	0.462	0.044	< 0.001	0.456	0.851
Equatorial Guinea	Male	0.464	1	1	0.996	0.997	1	0.999	1	0.926	0.936	0.649	0.006	0.572	0.363
Eritrea	Both	< 0.001	0.996	0.998	0.884	< 0.001	1	0.869	0.978	0.949	0.974	0.725	< 0.001	0.273	0.801
Eritrea	Female	< 0.001	1	1	0.957	< 0.001	1	0.972	0.999	0.994	0.965	0.408	< 0.001	0.359	0.916
Eritrea	Male	< 0.001	1	1	0.998	0.027	1	0.888	1	0.953	0.977	0.747	< 0.001	0.71	0.711
Estonia	Both	< 0.001	1	0.001	0.907	< 0.001	1	0.717	1	0.755	0.204	0.016	< 0.001	0.562	0.536
Estonia	Female	< 0.001	1	0.522	0.897	< 0.001	1	0.827	1	0.921	0.907	0.416	< 0.001	0.65	0.898
Estonia	Male	< 0.001	1	0.214	0.988	< 0.001	1	0.788	1	0.782	0.295	0.028	< 0.001	0.571	0.487
Eswatini	Both	0.008	1	1	0.999	0.424	1	0.953	1	0.799	0.783	0.274	< 0.001	0.882	0.275
Eswatini	Female	0.677	1	1	0.999	0.958	1	0.946	1	0.908	0.955	0.652	0.005	0.736	0.461
Eswatini	Male	0.14	1	1	1	0.904	1	0.997	1	0.955	0.922	0.336	0.005	0.953	0.44

Ethiopia	Both	< 0.001	< 0.001	< 0.001	< 0.001	< 0.001	0.38	0.174	< 0.001	< 0.001	< 0.001	< 0.001	< 0.001	< 0.001	< 0.001
Ethiopia	Female	< 0.001	< 0.001	< 0.001	< 0.001	< 0.001	0.826	0.059	< 0.001	< 0.001	< 0.001	< 0.001	< 0.001	< 0.001	< 0.001
Ethiopia	Male	< 0.001	< 0.001	< 0.001	0.002	< 0.001	0.924	0.978	< 0.001	< 0.001	< 0.001	< 0.001	< 0.001	< 0.001	< 0.001
Fiji	Both	< 0.001	1	1	1	0.493	1	0.938	1	0.969	0.619	0.097	< 0.001	0.832	0.637
Fiji	Female	< 0.001	1	1	1	0.002	1	0.912	1	0.965	0.758	0.17	0.002	0.827	0.709
Fiji	Male	< 0.001	1	1	1	0.025	1	0.988	1	0.996	0.944	0.332	0.087	0.931	0.788
Finland	Both	< 0.001	0.348	< 0.001	0.299	< 0.001	0.494	0.971	0.197	0.969	0.015	< 0.001	< 0.001	0.459	0.559
Finland	Female	< 0.001	0.315	< 0.001	0.443	< 0.001	0.654	0.928	0.159	0.947	0.351	0.026	< 0.001	0.653	0.569
Finland	Male	< 0.001	0.988	< 0.001	0.832	< 0.001	0.988	1	0.964	0.886	0.024	< 0.001	< 0.001	0.588	0.287
France	Both	< 0.001	< 0.001	< 0.001	0.519	< 0.001	< 0.001	0.003	< 0.001	< 0.001	< 0.001	< 0.001	< 0.001	0.686	0.046
France	Female	< 0.001	< 0.001	< 0.001	0.89	< 0.001	< 0.001	0.002	< 0.001	0.005	< 0.001	< 0.001	< 0.001	0.785	0.926
France	Male	< 0.001	0.001	< 0.001	0.43	< 0.001	< 0.001	0.214	< 0.001	< 0.001	< 0.001	< 0.001	< 0.001	0.49	< 0.001
Gabon	Both	< 0.001	1	1	0.986	0.014	1	0.968	1	0.992	0.998	0.922	< 0.001	0.446	0.989

Gabon	Female	< 0.001	1	1	0.988	0.072	1	0.971	1	0.993	0.997	0.753	< 0.001	0.57	0.91
Gabon	Male	< 0.001	1	1	0.999	0.673	1	0.993	1	0.997	0.999	0.899	< 0.001	0.633	0.849
Gambia	Both	< 0.001	1	1	0.997	0.154	1	0.987	1	0.997	0.912	0.284	< 0.001	0.717	0.872
Gambia	Female	0.035	1	1	0.999	0.528	1	0.99	1	0.998	0.974	0.443	0.002	0.796	0.891
Gambia	Male	0.112	1	1	1	0.9	1	0.998	1	1	0.983	0.435	< 0.001	0.845	0.973
Georgia	Both	< 0.001	< 0.001	< 0.001	0.612	< 0.001	< 0.001	< 0.001	< 0.001	< 0.001	< 0.001	< 0.001	< 0.001	0.42	< 0.001
Georgia	Female	< 0.001	< 0.001	< 0.001	0.857	0.007	< 0.001	< 0.001	< 0.001	< 0.001	< 0.001	< 0.001	< 0.001	0.362	< 0.001
Georgia	Male	< 0.001	0.955	< 0.001	0.992	< 0.001	0.992	0.039	0.875	< 0.001	< 0.001	< 0.001	< 0.001	0.552	< 0.001
Germany	Both	< 0.001	< 0.001	< 0.001	< 0.001	< 0.001	0.329	< 0.001	< 0.001	< 0.001	< 0.001	0.179	< 0.001	0.007	< 0.001
Germany	Female	< 0.001	< 0.001	< 0.001	< 0.001	< 0.001	0.009	< 0.001	< 0.001	< 0.001	< 0.001	0.227	< 0.001	0.049	< 0.001
Germany	Male	< 0.001	0.018	0.015	0.027	< 0.001	0.89	< 0.001	0.01	< 0.001	< 0.001	0.58	< 0.001	0.035	< 0.001
Ghana	Both	< 0.001	1	0.128	0.969	< 0.001	1	0.974	0.999	0.359	0.003	0.002	< 0.001	0.419	0.047
Ghana	Female	< 0.001	1	0.911	0.997	< 0.001	1	0.997	1	0.427	0.07	0.058	< 0.001	0.626	0.059

Ghana	Male	< 0.001	1	0.507	0.992	< 0.001	1	0.961	1	0.854	0.048	0.005	< 0.001	0.519	0.308
Greece	Both	< 0.001	< 0.001	< 0.001	0.016	< 0.001	0.002	0.08	0.003	0.121	< 0.001	< 0.001	< 0.001	0.6	0.662
Greece	Female	< 0.001	< 0.001	< 0.001	< 0.001	< 0.001	< 0.001	0.484	< 0.001	0.354	0.01	< 0.001	< 0.001	0.907	0.213
Greece	Male	< 0.001	0.748	< 0.001	0.996	< 0.001	0.69	0.097	0.961	0.176	< 0.001	< 0.001	< 0.001	0.927	0.795
Greenland	Both	0.259	1	1	1	1	1	0.997	1	1	0.999	0.716	0.008	0.938	0.962
Greenland	Female	0.831	1	1	1	1	1	0.976	1	0.995	0.997	0.718	0.125	0.961	0.964
Greenland	Male	0.699	1	1	1	1	1	0.999	1	1	1	0.933	0.034	0.902	0.944
Grenada	Both	0.924	1	1	1	1	1	0.998	1	0.999	1	0.992	0.111	0.844	0.855
Grenada	Female	0.998	1	1	1	1	1	0.994	1	0.994	0.998	0.896	0.373	0.846	0.7
Grenada	Male	1	1	1	1	1	1	0.954	1	0.922	0.966	0.942	0.286	0.925	0.445
Guam	Both	0.06	1	1	0.982	0.999	1	0.999	1	0.971	0.956	0.393	< 0.001	0.475	0.478
Guam	Female	0.461	1	1	0.992	0.921	1	0.999	1	0.99	0.95	0.32	0.023	0.629	0.607
Guam	Male	0.316	1	1	0.999	0.978	1	0.999	1	0.993	0.998	0.877	0.01	0.666	0.618
Guatemala	Both	< 0.001	< 0.001	< 0.001	0.091	< 0.001	0.004	0.97	< 0.001	0.49	0.013	< 0.001	< 0.001	0.908	0.066
Guatemala	Female	< 0.001	< 0.001	< 0.001	0.004	< 0.001	0.049	0.996	< 0.001	0.824	0.014	< 0.001	< 0.001	0.644	0.222

Guatemala	Male	< 0.001	0.404	0.28	0.397	< 0.001	0.339	0.97	0.272	0.665	0.781	0.545	< 0.001	0.222	0.125
Guinea	Both	< 0.001	1	0.495	0.976	< 0.001	1	0.945	1	0.897	0.296	0.018	< 0.001	0.788	0.456
Guinea	Female	< 0.001	1	1	0.997	< 0.001	1	0.986	1	0.995	0.985	0.483	< 0.001	0.908	0.822
Guinea	Male	< 0.001	1	0.46	0.996	< 0.001	1	0.94	1	0.88	0.199	0.009	< 0.001	0.799	0.427
Guinea-Bissau	Both	< 0.001	1	1	1	0.469	1	0.997	1	1	0.984	0.434	< 0.001	0.842	0.944
Guinea-Bissau	Female	0.096	1	1	1	0.85	1	0.998	1	1	0.996	0.572	0.001	0.848	0.939
Guinea-Bissau	Male	0.09	1	1	1	0.953	1	0.999	1	1	0.998	0.6	< 0.001	0.929	0.954
Guyana	Both	0.002	0.999	0.999	0.999	0.099	0.999	0.998	0.997	0.999	0.88	0.216	< 0.001	0.878	0.874
Guyana	Female	0.298	1	1	1	0.641	1	0.993	1	0.997	0.996	0.691	0.002	0.944	0.807
Guyana	Male	0.114	1	1	1	0.592	1	0.997	1	1	0.861	0.184	0.001	0.839	0.982
Haiti	Both	< 0.001	1	0.991	0.929	< 0.001	1	0.592	1	0.523	0.482	0.2	< 0.001	0.213	0.18
Haiti	Female	< 0.001	1	0.97	0.89	< 0.001	1	0.682	1	0.778	0.534	0.174	< 0.001	0.204	0.71
Haiti	Male	< 0.001	1	1	0.977	< 0.001	1	0.769	1	0.203	0.284	0.868	< 0.001	0.852	0.019

Honduras	Both	< 0.001	0.764	< 0.001	0.179	< 0.001	0.999	0.015	0.586	0.027	< 0.001	< 0.001	< 0.001	0.013	0.594
Honduras	Female	< 0.001	0.899	< 0.001	0.184	< 0.001	1	0.068	0.788	0.008	< 0.001	< 0.001	< 0.001	0.051	0.012
Honduras	Male	< 0.001	0.983	< 0.001	0.64	0.001	0.996	0.166	0.934	0.096	0.001	0.004	< 0.001	0.112	0.055
Hungary	Both	< 0.001	0.987	< 0.001	0.65	< 0.001	0.978	0.49	0.964	0.649	< 0.001	< 0.001	< 0.001	0.572	0.851
Hungary	Female	< 0.001	1	< 0.001	0.944	< 0.001	1	0.815	0.999	0.899	0.033	< 0.001	< 0.001	0.684	0.76
Hungary	Male	< 0.001	0.999	< 0.001	0.822	< 0.001	0.999	0.599	0.992	0.743	< 0.001	< 0.001	< 0.001	0.667	0.752
Iceland	Both	< 0.001	1	1	0.995	0.963	1	0.9	1	0.956	0.983	0.905	< 0.001	0.921	0.87
Iceland	Female	< 0.001	1	1	0.984	1	1	0.998	1	0.999	0.99	0.48	0.03	0.865	0.847
Iceland	Male	< 0.001	1	1	0.999	0.931	1	0.837	1	0.907	0.955	0.713	< 0.001	0.911	0.802
India	Both	< 0.001	0.342	< 0.001	0.668	< 0.001	0.511	0.04	0.451	0.015	< 0.001	< 0.001	< 0.001	0.61	0.198
India	Female	< 0.001	0.078	< 0.001	0.923	< 0.001	0.777	< 0.001	0.077	< 0.001	< 0.001	< 0.001	< 0.001	0.65	0.801
India	Male	< 0.001	0.006	< 0.001	0.028	< 0.001	0.004	0.758	0.017	0.058	< 0.001	< 0.001	< 0.001	0.137	0.011
Indonesia	Both	< 0.001	< 0.001	< 0.001	< 0.001	< 0.001	< 0.001	0.516	< 0.001	0.016	< 0.001	< 0.001	< 0.001	< 0.001	0.002

Indonesia	Female	< 0.001	< 0.001	< 0.001	< 0.001	< 0.001	< 0.001	0.623	< 0.001	0.602	< 0.001	< 0.001	< 0.001	< 0.001	0.443
Indonesia	Male	< 0.001	< 0.001	< 0.001	< 0.001	< 0.001	< 0.001	0.315	< 0.001	0.002	< 0.001	< 0.001	< 0.001	< 0.001	< 0.001
Iran (Islamic Republic of)	Both	< 0.001	0.969	0.78	0.627	< 0.001	0.986	0.034	0.915	< 0.001	< 0.001	0.02	< 0.001	0.129	< 0.001
Iran (Islamic Republic of)	Female	< 0.001	0.953	0.048	0.821	< 0.001	0.991	0.03	0.942	< 0.001	< 0.001	< 0.001	< 0.001	0.173	< 0.001
Iran (Islamic Republic of)	Male	< 0.001	0.431	0.321	0.019	< 0.001	0.892	0.492	0.261	< 0.001	< 0.001	0.09	< 0.001	0.012	< 0.001
Iraq	Both	< 0.001	0.482	< 0.001	0.287	< 0.001	0.924	0.14	0.354	0.059	< 0.001	< 0.001	< 0.001	0.073	0.024
Iraq	Female	< 0.001	0.853	0.011	0.355	< 0.001	0.817	0.163	0.738	0.211	< 0.001	< 0.001	< 0.001	0.238	0.206
Iraq	Male	< 0.001	0.09	< 0.001	0.009	< 0.001	1	0.708	0.038	0.223	< 0.001	< 0.001	< 0.001	< 0.001	0.032
Ireland	Both	< 0.001	< 0.001	< 0.001	< 0.001	< 0.001	< 0.001	< 0.001	< 0.001	< 0.001	< 0.001	< 0.001	< 0.001	0.279	0.459
Ireland	Female	< 0.001	< 0.001	< 0.001	0.003	0.214	0.073	0.01	< 0.001	0.017	< 0.001	< 0.001	< 0.001	0.378	0.625

Ireland	Male	< 0.001	0.475	< 0.001	0.39	< 0.001	0.439	0.001	0.314	0.002	< 0.001	< 0.001	< 0.001	0.803	0.132
Israel	Both	< 0.001	0.781	< 0.001	1	< 0.001	0.84	0.041	0.699	0.003	< 0.001	< 0.001	< 0.001	0.971	0.016
Israel	Female	< 0.001	0.982	< 0.001	0.994	0.005	0.974	0.147	0.973	0.025	< 0.001	< 0.001	< 0.001	0.737	0.037
Israel	Male	< 0.001	0.999	< 0.001	1	< 0.001	0.999	0.305	0.998	0.124	< 0.001	< 0.001	< 0.001	0.788	0.106
Italy	Both	< 0.001	< 0.001	< 0.001	< 0.001	< 0.001	< 0.001	< 0.001	< 0.001	< 0.001	< 0.001	< 0.001	< 0.001	0.127	< 0.001
Italy	Female	< 0.001	< 0.001	< 0.001	< 0.001	< 0.001	< 0.001	< 0.001	< 0.001	< 0.001	< 0.001	< 0.001	< 0.001	0.245	< 0.001
Italy	Male	< 0.001	< 0.001	< 0.001	< 0.001	< 0.001	< 0.001	< 0.001	< 0.001	< 0.001	< 0.001	< 0.001	< 0.001	0.002	0.518
Jamaica	Both	< 0.001	0.995	0.097	0.819	< 0.001	0.998	0.279	0.978	0.421	0.006	< 0.001	< 0.001	0.453	0.592
Jamaica	Female	< 0.001	1	0.987	0.937	< 0.001	1	0.182	1	0.128	0.053	0.124	< 0.001	0.659	0.224
Jamaica	Male	< 0.001	1	0.285	0.965	0.1	1	0.217	0.998	0.115	0.008	0.001	< 0.001	0.608	0.072
Japan	Both	< 0.001	< 0.001	< 0.001	0.118	< 0.001	0.085	< 0.001	< 0.001	< 0.001	< 0.001	< 0.001	< 0.001	0.02	< 0.001
Japan	Female	< 0.001	< 0.001	< 0.001	< 0.001	< 0.001	< 0.001	< 0.001	< 0.001	< 0.001	< 0.001	< 0.001	< 0.001	0.505	< 0.001
Japan	Male	< 0.001	< 0.001	< 0.001	< 0.001	< 0.001	< 0.001	< 0.001	< 0.001	< 0.001	< 0.001	< 0.001	< 0.001	< 0.001	0.001

Jordan	Both	< 0.001	1	0.968	0.957	< 0.001	1	0.916	1	0.547	0.267	0.023	< 0.001	0.397	0.186
Jordan	Female	< 0.001	1	0.999	0.99	< 0.001	1	0.925	1	0.8	0.606	0.09	< 0.001	0.475	0.415
Jordan	Male	< 0.001	1	1	0.99	< 0.001	1	0.939	1	0.873	0.792	0.182	< 0.001	0.53	0.416
Kazakhstan	Both	< 0.001	< 0.001	< 0.001	< 0.001	< 0.001	0.93	< 0.001	< 0.001	< 0.001	< 0.001	< 0.001	< 0.001	< 0.001	0.385
Kazakhstan	Female	< 0.001	< 0.001	< 0.001	< 0.001	< 0.001	0.962	< 0.001	< 0.001	0.002	< 0.001	0.08	< 0.001	< 0.001	0.612
Kazakhstan	Male	< 0.001	0.417	< 0.001	0.333	< 0.001	1	0.193	0.264	0.162	< 0.001	< 0.001	< 0.001	0.019	0.114
Kenya	Both	< 0.001	0.864	< 0.001	0.251	< 0.001	1	< 0.001	0.708	< 0.001	< 0.001	< 0.001	< 0.001	0.025	< 0.001
Kenya	Female	< 0.001	0.997	< 0.001	0.675	< 0.001	1	< 0.001	0.985	< 0.001	< 0.001	< 0.001	< 0.001	0.127	< 0.001
Kenya	Male	< 0.001	1	< 0.001	0.992	< 0.001	1	0.005	1	0.012	< 0.001	< 0.001	< 0.001	0.543	0.685
Kiribati	Both	0.996	1	1	1	0.999	1	1	1	1	1	0.932	0.474	0.914	0.961
Kiribati	Female	0.886	1	1	1	0.844	1	1	1	1	1	0.992	0.579	0.918	0.939
Kiribati	Male	0.988	1	1	1	0.993	1	1	1	1	1	0.891	0.65	0.974	0.986
Kuwait	Both	< 0.001	1	1	0.995	0.045	1	0.783	1	0.772	0.823	0.78	< 0.001	0.471	0.353
Kuwait	Female	< 0.001	1	1	0.994	0.868	1	0.979	1	0.982	0.896	0.386	< 0.001	0.501	0.664

Kuwait	Male	< 0.001	1	1	0.998	0.136	1	0.786	1	0.82	0.886	0.524	< 0.001	0.599	0.413
Kyrgyzstan	Both	< 0.001	< 0.001	< 0.001	< 0.001	< 0.001	< 0.001	0.024	< 0.001	0.002	< 0.001	0.004	< 0.001	0.013	0.009
Kyrgyzstan	Female	< 0.001	< 0.001	< 0.001	< 0.001	< 0.001	0.004	0.018	< 0.001	0.003	0.004	0.114	< 0.001	0.095	0.022
Kyrgyzstan	Male	< 0.001	0.954	0.838	0.259	< 0.001	0.993	0.703	0.866	0.621	0.381	0.079	< 0.001	0.207	0.265
Lao People's Democratic Republic	Both	< 0.001	1	0.923	0.946	< 0.001	1	0.997	1	1	0.254	0.013	< 0.001	0.824	0.954
Lao People's Democratic Republic	Female	< 0.001	1	0.987	0.983	< 0.001	1	0.998	1	1	0.52	0.046	< 0.001	0.969	0.955
Lao People's Democratic Republic	Male	< 0.001	1	1	0.995	0.009	1	0.999	1	1	0.839	0.168	< 0.001	0.785	0.911
Latvia	Both	< 0.001	1	< 0.001	1	< 0.001	1	0.131	0.998	0.213	0.016	0.005	< 0.001	0.827	0.545
Latvia	Female	< 0.001	0.999	0.352	0.985	< 0.001	0.999	0.61	0.998	0.381	0.379	0.339	< 0.001	0.72	0.105
Latvia	Male	< 0.001	1	0.046	1	< 0.001	1	0.175	1	0.188	0.025	0.006	< 0.001	0.937	0.363

Lebanon	Both	< 0.001	1	1	0.995	< 0.001	1	1	1	1	1	0.734	< 0.001	0.789	0.984
Lebanon	Female	< 0.001	1	0.68	0.998	0.002	1	0.881	0.999	0.81	0.371	0.087	< 0.001	0.815	0.267
Lebanon	Male	< 0.001	1	0.995	0.998	0.006	1	0.86	1	0.826	0.479	0.13	< 0.001	0.715	0.3
Lesotho	Both	< 0.001	1	0.044	0.919	0.007	1	0.71	1	0.829	0.003	< 0.001	< 0.001	0.754	0.822
Lesotho	Female	0.007	1	0.15	0.956	0.494	1	0.504	1	0.672	0.043	0.003	< 0.001	0.618	0.832
Lesotho	Male	< 0.001	1	0.967	1	0.306	1	1	1	0.946	0.322	0.02	< 0.001	0.769	0.394
Liberia	Both	< 0.001	1	1	0.981	< 0.001	1	0.796	1	0.819	0.828	0.498	< 0.001	0.764	0.401
Liberia	Female	< 0.001	1	1	0.996	0.043	1	0.897	1	0.946	0.967	0.698	< 0.001	0.81	0.622
Liberia	Male	< 0.001	1	1	0.998	0.206	1	0.937	1	0.944	0.95	0.584	< 0.001	0.835	0.518
Libya	Both	< 0.001	1	0.978	0.892	< 0.001	1	0.974	1	0.987	0.634	0.111	< 0.001	0.461	0.669
Libya	Female	< 0.001	1	1	0.97	0.01	1	0.953	1	0.968	0.878	0.348	< 0.001	0.566	0.555
Libya	Male	< 0.001	1	1	0.988	0.062	1	0.985	1	0.997	0.842	0.184	< 0.001	0.669	0.978
Lithuania	Both	< 0.001	0.999	0.004	1	< 0.001	0.999	0.303	0.993	0.084	0.018	0.01	< 0.001	0.944	0.049

Lithuania	Female	< 0.001	1	0.606	1	< 0.001	1	0.524	0.998	0.275	0.31	0.279	< 0.001	0.888	0.119
Lithuania	Male	< 0.001	1	0.134	0.994	< 0.001	1	0.68	1	0.611	0.146	0.014	< 0.001	0.769	0.323
Luxembourg	Both	< 0.001	1	1	0.999	0.659	1	0.777	1	0.152	0.164	0.505	< 0.001	0.875	0.028
Luxembourg	Female	< 0.001	1	1	0.996	0.974	1	0.988	1	0.668	0.655	0.449	0.001	0.821	0.15
Luxembourg	Male	< 0.001	1	1	0.999	0.649	1	0.736	1	0.391	0.458	0.664	< 0.001	0.924	0.135
Madagascar	Both	< 0.001	1	1	0.989	< 0.001	1	0.046	0.999	0.076	0.076	0.226	< 0.001	0.761	0.295
Madagascar	Female	< 0.001	1	1	0.951	< 0.001	1	0.234	1	0.368	0.307	0.186	< 0.001	0.737	0.639
Madagascar	Male	< 0.001	1	1	1	< 0.001	1	0.253	1	0.284	0.313	0.375	< 0.001	0.91	0.236
Malawi	Both	< 0.001	0.994	0.589	0.939	< 0.001	1	0.931	0.969	0.756	0.337	0.093	< 0.001	0.272	0.233
Malawi	Female	< 0.001	0.998	0.985	0.966	< 0.001	1	0.941	0.986	0.247	0.335	0.926	< 0.001	0.252	0.024
Malawi	Male	< 0.001	1	0.982	1	< 0.001	1	0.984	1	0.95	0.491	0.045	< 0.001	0.792	0.45
Malaysia	Both	< 0.001	0.001	< 0.001	< 0.001	< 0.001	0.004	0.002	0.001	< 0.001	< 0.001	< 0.001	< 0.001	0.56	< 0.001
Malaysia	Female	< 0.001	< 0.001	< 0.001	< 0.001	< 0.001	0.019	0.006	< 0.001	0.001	< 0.001	< 0.001	< 0.001	0.197	0.002

Malaysia	Male	< 0.001	0.962	0.003	0.495	< 0.001	0.943	0.163	0.939	0.105	< 0.001	< 0.001	< 0.001	0.69	0.083
Maldives	Both	0.327	1	1	1	0.987	1	0.999	1	0.999	1	0.78	0.002	0.881	0.877
Maldives	Female	0.905	1	1	1	0.999	1	1	1	1	1	0.758	0.035	0.905	0.936
Maldives	Male	0.819	1	1	1	0.999	1	1	1	1	1	0.896	0.021	0.917	0.912
Mali	Both	< 0.001	1	0.835	0.929	< 0.001	1	0.997	0.999	0.998	0.529	0.043	< 0.001	0.697	0.789
Mali	Female	< 0.001	1	1	0.998	< 0.001	1	0.996	1	0.94	0.93	0.366	< 0.001	0.839	0.378
Mali	Male	< 0.001	1	0.87	0.89	< 0.001	1	0.969	0.998	0.981	0.428	0.047	< 0.001	0.614	0.616
Malta	Both	< 0.001	1	0.996	0.997	0.995	1	0.985	1	0.997	0.993	0.58	< 0.001	0.955	0.853
Malta	Female	< 0.001	1	1	0.997	0.998	1	0.984	1	0.997	0.997	0.699	0.002	0.992	0.984
Malta	Male	< 0.001	1	1	1	0.985	1	0.989	1	0.994	0.99	0.542	< 0.001	0.963	0.718
Marshall Islands	Both	1	1	1	1	1	1	1	1	1	1	0.759	0.409	0.906	0.84
Marshall Islands	Female	0.997	1	1	1	0.995	1	1	1	0.999	1	0.757	0.532	0.931	0.792
Marshall Islands	Male	0.997	1	1	1	0.999	1	1	1	1	1	0.895	0.578	0.967	0.998
Mauritania	Both	< 0.001	1	1	0.998	< 0.001	1	0.977	1	0.986	0.996	0.842	< 0.001	0.923	0.651

Mauritania	Female	< 0.001	1	1	0.999	0.019	1	0.984	1	0.997	0.999	0.928	< 0.001	0.956	0.894
Mauritania	Male	< 0.001	1	1	0.999	0.303	1	0.994	1	0.986	0.996	0.92	< 0.001	0.876	0.568
Mauritius	Both	< 0.001	1	1	0.992	< 0.001	1	0.909	1	0.938	0.976	0.974	< 0.001	0.907	0.554
Mauritius	Female	< 0.001	1	1	0.992	0.002	1	0.926	1	0.977	0.988	0.716	< 0.001	0.874	0.956
Mauritius	Male	< 0.001	1	1	0.999	0.014	1	0.987	1	0.948	0.974	0.62	< 0.001	0.894	0.43
Mexico	Both	< 0.001	< 0.001	< 0.001	< 0.001	< 0.001	< 0.001	0.008	< 0.001	0.012	0.009	0.208	< 0.001	0.065	0.854
Mexico	Female	< 0.001	< 0.001	< 0.001	< 0.001	< 0.001	< 0.001	0.002	< 0.001	0.006	0.002	0.044	< 0.001	0.231	0.298
Mexico	Male	< 0.001	< 0.001	< 0.001	< 0.001	< 0.001	< 0.001	0.121	< 0.001	0.024	< 0.001	< 0.001	< 0.001	0.008	0.066
Micronesia (Federated States of)	Both	0.989	1	1	1	0.999	1	1	1	1	0.999	0.623	0.253	0.996	0.981
Micronesia (Federated States of)	Female	0.916	1	1	1	0.897	1	1	1	1	1	0.74	0.35	0.977	0.975
Micronesia	Male	0.923	1	1	1	0.946	1	1	1	1	1	0.746	0.471	0.974	0.947

(Federated States of)															
Monaco	Both	0.418	1	1	1	1	1	1	1	1	1	0.787	0.132	0.936	0.951
Monaco	Female	0.684	1	1	1	1	1	1	1	1	1	0.891	0.469	0.962	0.995
Monaco	Male	0.887	1	1	1	1	1	1	1	1	1	0.786	0.178	0.963	0.926
Mongolia	Both	< 0.001	0.999	0.999	0.876	< 0.001	0.998	0.763	0.994	0.689	0.813	0.947	< 0.001	0.804	0.237
Mongolia	Female	< 0.001	1	1	0.873	0.001	1	0.938	0.999	0.976	0.989	0.755	< 0.001	0.663	0.741
Mongolia	Male	< 0.001	1	1	0.999	< 0.001	1	0.84	1	0.641	0.76	0.69	< 0.001	0.896	0.157
Montenegro	Both	< 0.001	0.584	0.002	0.397	< 0.001	0.999	0.6	0.43	0.737	0.644	0.262	< 0.001	0.159	0.627
Montenegro	Female	< 0.001	0.992	0.764	0.748	0.031	1	0.659	0.963	0.808	0.865	0.589	< 0.001	0.565	0.944
Montenegro	Male	< 0.001	0.862	0.121	0.84	0.005	1	0.707	0.728	0.788	0.752	0.377	< 0.001	0.225	0.523
Morocco	Both	< 0.001	< 0.001	< 0.001	< 0.001	< 0.001	0.939	0.885	< 0.001	< 0.001	< 0.001	< 0.001	< 0.001	< 0.001	< 0.001
Morocco	Female	< 0.001	< 0.001	< 0.001	< 0.001	< 0.001	0.007	0.081	< 0.001	0.143	< 0.001	< 0.001	< 0.001	0.004	0.841
Morocco	Male	< 0.001	0.097	< 0.001	0.373	< 0.001	0.792	0.129	0.087	< 0.001	< 0.001	0.174	< 0.001	0.01	< 0.001
Mozambique	Both	< 0.001	1	< 0.001	0.94	< 0.001	0.999	0.21	0.998	0.081	< 0.001	< 0.001	< 0.001	0.836	0.022

Mozambique	Female	< 0.001	0.993	< 0.001	0.854	< 0.001	1	0.762	0.977	0.037	< 0.001	< 0.001	< 0.001	0.275	0.002
Mozambique	Male	< 0.001	1	< 0.001	0.975	< 0.001	1	0.254	1	0.359	< 0.001	< 0.001	< 0.001	0.41	0.851
Myanmar	Both	< 0.001	< 0.001	< 0.001	< 0.001	< 0.001	0.963	0.419	< 0.001	0.47	< 0.001	< 0.001	< 0.001	< 0.001	0.421
Myanmar	Female	< 0.001	< 0.001	< 0.001	0.001	< 0.001	0.999	0.13	< 0.001	0.227	0.022	0.008	< 0.001	0.003	0.868
Myanmar	Male	< 0.001	0.137	< 0.001	0.034	< 0.001	0.998	0.973	0.056	0.77	0.005	< 0.001	< 0.001	0.014	0.188
Namibia	Both	< 0.001	1	1	0.988	0.012	1	0.802	1	0.91	0.932	0.581	< 0.001	0.449	0.874
Namibia	Female	0.01	1	1	0.996	0.376	1	0.9	1	0.963	0.987	0.944	< 0.001	0.554	0.976
Namibia	Male	< 0.001	1	1	0.999	0.282	1	0.937	1	0.979	0.953	0.411	< 0.001	0.601	0.826
Nauru	Both	1	1	1	1	1	1	1	1	1	1	0.972	0.796	0.982	0.879
Nauru	Female	1	1	1	1	1	1	1	1	1	1	0.985	0.847	0.978	0.892
Nauru	Male	1	1	1	1	1	1	1	1	1	1	0.963	0.853	0.994	0.945
Nepal	Both	< 0.001	< 0.001	< 0.001	< 0.001	< 0.001	0.748	0.964	< 0.001	< 0.001	< 0.001	< 0.001	< 0.001	< 0.001	< 0.001
Nepal	Female	< 0.001	< 0.001	< 0.001	< 0.001	< 0.001	0.952	0.982	< 0.001	0.015	< 0.001	< 0.001	< 0.001	0.003	< 0.001
Nepal	Male	< 0.001	0.221	< 0.001	0.057	< 0.001	0.999	0.984	0.101	0.077	< 0.001	< 0.001	< 0.001	0.025	0.007

Netherlands	Both	< 0.001	0.007	< 0.001	0.426	< 0.001	0.065	< 0.001	0.002	< 0.001	< 0.001	< 0.001	< 0.001	0.915	< 0.001
Netherlands	Female	< 0.001	0.071	< 0.001	0.99	< 0.001	0.058	< 0.001	0.045	< 0.001	< 0.001	< 0.001	< 0.001	0.686	0.941
Netherlands	Male	< 0.001	0.036	< 0.001	0.839	< 0.001	0.372	< 0.001	0.012	< 0.001	< 0.001	< 0.001	< 0.001	0.602	< 0.001
New Zealand	Both	< 0.001	0.353	0.006	0.052	< 0.001	0.802	0.769	0.182	0.569	0.341	0.062	< 0.001	0.854	0.236
New Zealand	Female	< 0.001	0.384	0.036	0.04	< 0.001	1	0.627	0.205	0.527	0.152	0.017	< 0.001	0.475	0.298
New Zealand	Male	< 0.001	0.702	0.369	0.347	< 0.001	0.639	0.998	0.492	0.896	0.953	0.966	< 0.001	0.882	0.325
Nicaragua	Both	< 0.001	0.047	< 0.001	0.001	< 0.001	0.568	0.046	0.021	< 0.001	< 0.001	0.003	< 0.001	0.08	< 0.001
Nicaragua	Female	< 0.001	0.158	< 0.001	0.011	< 0.001	0.739	< 0.001	0.081	< 0.001	< 0.001	0.013	< 0.001	0.231	< 0.001
Nicaragua	Male	< 0.001	0.999	0.855	0.742	< 0.001	1	0.958	0.995	0.942	0.653	0.087	< 0.001	0.294	0.579
Niger	Both	< 0.001	1	0.996	0.962	< 0.001	1	0.973	0.998	0.994	0.872	0.22	< 0.001	0.59	0.961
Niger	Female	< 0.001	1	0.999	0.982	< 0.001	1	0.977	1	0.99	0.926	0.277	< 0.001	0.639	0.751
Niger	Male	< 0.001	1	1	0.991	< 0.001	1	0.988	1	0.997	0.963	0.396	< 0.001	0.759	0.796
Nigeria	Both	< 0.001	0.944	< 0.001	0.89	< 0.001	0.991	< 0.001	0.908	< 0.001	< 0.001	< 0.001	< 0.001	0.235	0.357

Nigeria	Female	< 0.001	1	< 0.001	0.952	< 0.001	1	0.001	0.997	0.003	< 0.001	0.001	< 0.001	0.495	0.904
Nigeria	Male	< 0.001	1	1	0.922	< 0.001	1	0.139	1	0.24	0.357	0.963	< 0.001	0.733	0.761
Niue	Both	1	1	1	1	1	1	1	1	1	1	0.995	0.773	0.986	0.98
Niue	Female	1	1	1	1	1	1	1	1	1	1	0.991	0.853	0.993	0.985
Niue	Male	1	1	1	1	1	1	1	1	1	1	0.995	0.837	0.968	0.988
North Macedonia	Both	< 0.001	0.902	0.894	0.337	< 0.001	0.971	0.98	0.822	0.57	0.678	0.491	< 0.001	0.392	0.114
North Macedonia	Female	< 0.001	0.998	0.993	0.793	< 0.001	1	0.982	0.992	0.391	0.485	0.407	< 0.001	0.619	0.057
North Macedonia	Male	< 0.001	0.974	0.982	0.451	< 0.001	0.994	0.993	0.921	0.99	0.998	0.898	< 0.001	0.352	0.677
Northern Mariana Islands	Both	0.999	1	1	1	1	1	0.982	1	0.988	0.996	0.78	0.295	0.938	0.763
Northern Mariana Islands	Female	0.994	1	1	1	0.99	1	0.969	1	0.965	0.989	0.942	0.506	0.993	0.636
Northern Mariana Islands	Male	0.961	1	1	1	0.976	1	1	1	1	1	0.728	0.409	0.923	0.961

Norway	Both	< 0.001	< 0.001	< 0.001	< 0.001	< 0.001	< 0.001	0.807	< 0.001	0.57	0.002	< 0.001	< 0.001	0.03	0.165
Norway	Female	< 0.001	< 0.001	< 0.001	< 0.001	< 0.001	< 0.001	0.912	< 0.001	0.43	0.14	0.023	< 0.001	0.206	0.068
Norway	Male	< 0.001	< 0.001	< 0.001	< 0.001	< 0.001	0.004	0.792	< 0.001	0.9	0.002	< 0.001	< 0.001	0.175	0.861
Oman	Both	0.006	0.999	1	0.967	0.025	1	0.975	0.997	0.961	0.987	0.876	< 0.001	0.293	0.57
Oman	Female	0.223	1	1	0.987	0.969	1	0.991	1	0.993	0.998	0.747	0.001	0.483	0.746
Oman	Male	0.008	0.998	0.998	0.991	0.006	1	0.993	0.992	0.995	0.996	0.739	0.031	0.43	0.765
Pakistan	Both	< 0.001	< 0.001	< 0.001	< 0.001	< 0.001	0.023	0.48	< 0.001	< 0.001	< 0.001	< 0.001	< 0.001	0.005	< 0.001
Pakistan	Female	< 0.001	< 0.001	< 0.001	< 0.001	< 0.001	0.719	0.684	< 0.001	0.001	< 0.001	< 0.001	< 0.001	0.006	< 0.001
Pakistan	Male	< 0.001	0.407	< 0.001	0.012	< 0.001	0.683	0.662	0.228	< 0.001	< 0.001	< 0.001	< 0.001	0.548	< 0.001
Palau	Both	1	1	1	1	1	1	1	1	1	1	0.971	0.476	0.999	0.961
Palau	Female	1	1	1	1	1	1	1	1	1	1	0.949	0.674	0.986	0.956
Palau	Male	1	1	1	1	1	1	1	1	1	1	0.973	0.565	0.99	0.997
Palestine	Both	< 0.001	1	1	0.99	0.007	1	0.88	1	0.947	0.764	0.218	< 0.001	0.767	0.866
Palestine	Female	< 0.001	1	1	0.996	0.002	1	0.943	1	0.974	0.937	0.442	< 0.001	0.946	0.779

Palestine	Male	< 0.001	1	1	0.997	0.016	1	0.949	1	0.984	0.961	0.466	< 0.001	0.675	0.946
Panama	Both	< 0.001	1	0.996	0.999	< 0.001	1	0.971	0.999	0.991	0.753	0.154	< 0.001	0.841	0.918
Panama	Female	< 0.001	1	1	0.996	0.012	1	0.997	0.999	0.998	0.979	0.492	< 0.001	0.971	0.815
Panama	Male	< 0.001	1	1	1	0.041	1	0.928	1	0.977	0.803	0.19	< 0.001	0.766	0.937
Papua New Guinea	Both	< 0.001	1	0.509	0.81	< 0.001	1	0.995	0.999	0.976	0.118	0.003	< 0.001	0.183	0.569
Papua New Guinea	Female	< 0.001	1	0.905	0.79	< 0.001	1	0.997	0.998	0.987	0.431	0.028	< 0.001	0.158	0.628
Papua New Guinea	Male	< 0.001	1	0.992	0.999	< 0.001	1	0.999	1	0.999	0.533	0.043	< 0.001	0.994	0.796
Paraguay	Both	< 0.001	0.752	0.239	0.205	< 0.001	0.796	0.392	0.604	0.414	0.178	0.033	< 0.001	0.604	0.18
Paraguay	Female	< 0.001	0.905	0.922	0.378	< 0.001	0.925	0.604	0.8	0.518	0.662	0.736	< 0.001	0.868	0.146
Paraguay	Male	< 0.001	1	0.543	0.957	< 0.001	1	0.764	0.999	0.884	0.123	0.008	< 0.001	0.673	0.732
Peru	Both	< 0.001	0.55	< 0.001	0.34	< 0.001	0.55	< 0.001	0.382	< 0.001	< 0.001	< 0.001	< 0.001	0.784	0.457

Peru	Female	< 0.001	< 0.001	< 0.001	< 0.001	< 0.001	0.004	< 0.001	< 0.001	< 0.001	< 0.001	< 0.001	< 0.001	0.508	0.267
Peru	Male	< 0.001	0.889	< 0.001	0.558	< 0.001	0.991	0.002	0.83	< 0.001	< 0.001	< 0.001	< 0.001	0.253	0.027
Philippines	Both	< 0.001	< 0.001	< 0.001	< 0.001	< 0.001	0.004	0.004	< 0.001	< 0.001	< 0.001	< 0.001	< 0.001	< 0.001	< 0.001
Philippines	Female	< 0.001	< 0.001	< 0.001	< 0.001	< 0.001	0.023	0.006	< 0.001	< 0.001	< 0.001	< 0.001	< 0.001	< 0.001	< 0.001
Philippines	Male	< 0.001	< 0.001	< 0.001	< 0.001	< 0.001	0.021	0.014	< 0.001	< 0.001	< 0.001	< 0.001	< 0.001	< 0.001	< 0.001
Poland	Both	< 0.001	0.534	< 0.001	0.54	< 0.001	0.463	< 0.001	0.538	< 0.001	< 0.001	< 0.001	< 0.001	0.402	< 0.001
Poland	Female	< 0.001	0.44	< 0.001	0.586	< 0.001	0.495	0.001	0.652	< 0.001	< 0.001	0.009	< 0.001	0.287	< 0.001
Poland	Male	< 0.001	0.169	< 0.001	0.224	< 0.001	0.131	< 0.001	0.098	< 0.001	< 0.001	< 0.001	< 0.001	0.323	< 0.001
Portugal	Both	< 0.001	0.853	< 0.001	0.733	< 0.001	0.98	< 0.001	0.689	< 0.001	< 0.001	< 0.001	< 0.001	0.533	0.982
Portugal	Female	< 0.001	0.998	< 0.001	0.979	< 0.001	0.999	< 0.001	0.986	0.001	< 0.001	< 0.001	< 0.001	0.697	0.884
Portugal	Male	< 0.001	0.999	< 0.001	0.99	< 0.001	0.999	0.059	0.995	0.09	< 0.001	< 0.001	< 0.001	0.475	0.596
Puerto Rico	Both	< 0.001	1	1	0.991	< 0.001	1	0.973	1	0.703	0.673	0.462	< 0.001	0.921	0.147
Puerto Rico	Female	< 0.001	1	1	0.988	< 0.001	1	0.996	1	0.846	0.903	0.798	< 0.001	0.871	0.24

Puerto Rico	Male	< 0.001	1	1	1	< 0.001	1	0.975	1	0.923	0.889	0.469	< 0.001	0.886	0.376
Qatar	Both	< 0.001	0.966	0.508	0.95	0.329	0.975	0.995	0.898	0.999	0.484	0.046	< 0.001	0.59	0.876
Qatar	Female	0.063	0.994	0.996	0.97	0.411	1	0.998	0.973	0.999	0.999	0.702	0.012	0.421	0.851
Qatar	Male	0.004	0.999	0.795	0.984	0.279	0.999	0.986	0.994	0.997	0.816	0.155	0.024	0.73	0.949
Republic of Korea	Both	< 0.001	0.016	< 0.001	< 0.001	< 0.001	0.691	0.749	0.012	< 0.001	< 0.001	< 0.001	< 0.001	0.007	< 0.001
Republic of Korea	Female	< 0.001	0.089	< 0.001	0.002	< 0.001	0.997	0.815	0.033	0.007	< 0.001	< 0.001	< 0.001	0.057	< 0.001
Republic of Korea	Male	< 0.001	0.666	< 0.001	0.274	< 0.001	0.603	0.81	0.622	0.061	< 0.001	< 0.001	< 0.001	0.557	0.009
Republic of Moldova	Both	< 0.001	0.568	0.266	0.199	< 0.001	0.999	0.405	0.399	0.571	0.601	0.361	< 0.001	0.264	0.975
Republic of Moldova	Female	< 0.001	0.969	0.209	0.714	< 0.001	1	0.506	0.907	0.655	0.518	0.179	< 0.001	0.65	0.665
Republic of Moldova	Male	< 0.001	0.999	0.999	0.819	< 0.001	1	0.693	0.993	0.743	0.854	0.978	< 0.001	0.5	0.501
Romania	Both	< 0.001	0.299	< 0.001	0.472	< 0.001	0.657	< 0.001	0.161	< 0.001	< 0.001	< 0.001	< 0.001	0.874	< 0.001
Romania	Female	< 0.001	0.386	< 0.001	0.564	< 0.001	0.86	< 0.001	0.236	< 0.001	< 0.001	< 0.001	< 0.001	0.849	< 0.001

Romania	Male	< 0.001	0.987	< 0.001	0.886	< 0.001	1	< 0.001	0.953	< 0.001	< 0.001	< 0.001	< 0.001	0.643	0.003
Russian Federation	Both	< 0.001	< 0.001	< 0.001	< 0.001	< 0.001	< 0.001	0.015	< 0.001	< 0.001	< 0.001	< 0.001	< 0.001	0.938	< 0.001
Russian Federation	Female	< 0.001	< 0.001	< 0.001	< 0.001	< 0.001	< 0.001	0.004	< 0.001	< 0.001	< 0.001	< 0.001	< 0.001	0.667	< 0.001
Russian Federation	Male	< 0.001	< 0.001	< 0.001	< 0.001	< 0.001	< 0.001	0.069	< 0.001	< 0.001	< 0.001	< 0.001	< 0.001	0.013	< 0.001
Rwanda	Both	< 0.001	0.009	< 0.001	0.039	< 0.001	0.986	0.051	0.002	< 0.001	< 0.001	< 0.001	< 0.001	0.007	< 0.001
Rwanda	Female	< 0.001	0.444	< 0.001	0.272	< 0.001	1	0.31	0.253	< 0.001	< 0.001	< 0.001	< 0.001	0.036	< 0.001
Rwanda	Male	< 0.001	0.73	< 0.001	0.528	< 0.001	0.999	0.196	0.52	0.015	< 0.001	< 0.001	< 0.001	0.151	0.008
Saint Kitts and Nevis	Both	0.993	1	1	0.995	0.992	1	0.993	1	0.991	0.987	0.505	0.338	0.578	0.679
Saint Kitts and Nevis	Female	1	1	1	0.998	1	1	0.997	1	0.999	0.997	0.599	0.514	0.65	0.857
Saint Kitts and Nevis	Male	1	1	1	0.999	0.999	1	0.996	1	0.993	0.995	0.628	0.67	0.712	0.672
Saint Lucia	Both	0.894	1	1	1	0.846	1	0.877	1	0.798	0.854	0.427	0.156	0.965	0.411
Saint Lucia	Female	0.993	1	1	1	0.986	1	0.954	1	0.87	0.917	0.499	0.345	0.975	0.403
Saint Lucia	Male	0.99	1	1	1	0.98	1	0.944	1	0.97	0.983	0.613	0.366	0.89	0.784

Saint Vincent and the Grenadines	Both	0.991	1	1	1	0.991	1	0.98	1	0.829	0.896	0.874	0.214	0.77	0.236
Saint Vincent and the Grenadines	Female	0.999	1	1	1	0.999	1	0.963	1	0.85	0.925	0.999	0.443	0.798	0.272
Saint Vincent and the Grenadines	Male	1	1	1	1	0.999	1	0.999	1	0.988	0.996	0.923	0.405	0.925	0.583
Samoa	Both	0.675	1	1	1	0.99	1	1	1	1	1	0.977	0.017	0.827	0.845
Samoa	Female	0.657	1	1	1	0.705	1	1	1	1	1	0.774	0.117	0.794	0.897
Samoa	Male	0.715	1	1	1	0.966	1	1	1	1	1	0.856	0.068	0.989	0.898
San Marino	Both	0.845	1	1	1	1	1	0.999	1	1	1	0.851	0.187	0.903	0.864
San Marino	Female	0.932	1	1	1	1	1	0.997	1	1	1	0.987	0.51	0.968	0.938
San Marino	Male	0.978	1	1	1	1	1	1	1	1	1	0.833	0.228	0.967	0.882

Sao Tome and Principe	Both	0.97	1	1	1	1	1	1	1	1	0.997	0.608	0.119	0.85	0.877
Sao Tome and Principe	Female	0.998	1	1	1	1	1	1	1	1	1	0.739	0.301	0.878	0.928
Sao Tome and Principe	Male	0.999	1	1	1	1	1	1	1	1	0.999	0.724	0.245	0.911	0.899
Saudi Arabia	Both	< 0.001	0.933	0.953	0.46	< 0.001	0.994	0.47	0.825	0.182	0.236	0.214	< 0.001	0.31	0.036
Saudi Arabia	Female	< 0.001	1	0.646	0.911	< 0.001	1	0.257	0.997	0.392	0.025	0.003	< 0.001	0.641	0.97
Saudi Arabia	Male	< 0.001	0.916	0.377	0.208	< 0.001	0.982	0.908	0.796	0.101	0.003	< 0.001	< 0.001	0.168	0.009
Senegal	Both	< 0.001	1	0.618	0.899	< 0.001	1	0.846	0.998	0.936	0.257	0.02	< 0.001	0.714	0.878
Senegal	Female	< 0.001	1	0.991	0.987	< 0.001	1	0.912	1	0.967	0.781	0.193	< 0.001	0.716	0.804
Senegal	Male	< 0.001	1	0.949	0.968	< 0.001	1	0.957	1	0.988	0.48	0.043	< 0.001	0.827	0.951
Serbia	Both	< 0.001	< 0.001	< 0.001	< 0.001	< 0.001	< 0.001	0.004	< 0.001	< 0.001	< 0.001	0.136	< 0.001	< 0.001	< 0.001
Serbia	Female	< 0.001	< 0.001	< 0.001	< 0.001	< 0.001	0.014	0.042	< 0.001	< 0.001	< 0.001	0.012	< 0.001	0.01	< 0.001

Serbia	Male	< 0.001	< 0.001	< 0.001	< 0.001	< 0.001	0.041	0.132	< 0.001	< 0.001	< 0.001	0.942	< 0.001	0.011	< 0.001
Seychelles	Both	0.822	1	1	1	1	1	1	1	1	1	0.965	0.023	0.927	0.994
Seychelles	Female	0.966	1	1	1	1	1	1	1	1	1	0.94	0.116	0.951	0.998
Seychelles	Male	0.993	1	1	1	1	1	0.999	1	1	1	0.837	0.12	0.972	0.96
Sierra Leone	Both	< 0.001	1	0.989	0.975	< 0.001	1	0.972	1	0.993	0.782	0.132	< 0.001	0.805	0.957
Sierra Leone	Female	< 0.001	1	0.999	0.999	< 0.001	1	0.983	1	0.99	0.884	0.206	< 0.001	0.866	0.768
Sierra Leone	Male	< 0.001	1	1	0.992	0.04	1	0.994	1	0.999	0.978	0.406	< 0.001	0.837	0.93
Singapore	Both	< 0.001	1	< 0.001	0.983	< 0.001	1	0.943	0.997	0.907	< 0.001	< 0.001	< 0.001	0.796	0.358
Singapore	Female	< 0.001	1	0.011	0.967	< 0.001	1	0.97	1	0.989	0.004	< 0.001	< 0.001	0.694	0.74
Singapore	Male	< 0.001	1	0.003	0.998	< 0.001	1	0.976	1	0.941	< 0.001	< 0.001	< 0.001	0.892	0.398
Slovakia	Both	< 0.001	0.938	0.745	0.392	< 0.001	1	0.018	0.859	< 0.001	< 0.001	0.822	< 0.001	0.182	< 0.001
Slovakia	Female	< 0.001	0.76	0.796	0.189	< 0.001	1	0.399	0.581	< 0.001	< 0.001	0.706	< 0.001	0.433	< 0.001
Slovakia	Male	< 0.001	0.998	0.959	0.927	< 0.001	1	0.035	0.99	< 0.001	< 0.001	0.643	< 0.001	0.298	< 0.001
Slovenia	Both	< 0.001	1	0.735	0.962	< 0.001	1	0.795	1	0.406	0.172	0.087	< 0.001	0.733	0.082

Slovenia	Female	< 0.001	1	0.07	0.916	< 0.001	1	0.975	0.999	0.959	0.526	0.071	< 0.001	0.803	0.53
Slovenia	Male	< 0.001	0.963	0.955	0.502	< 0.001	0.999	0.705	0.892	0.48	0.542	0.575	< 0.001	0.437	0.138
Solomon Islands	Both	0.15	1	1	1	0.895	1	0.999	1	1	0.995	0.539	0.058	0.994	0.891
Solomon Islands	Female	0.04	1	1	1	0.05	1	0.999	1	1	0.999	0.645	0.183	0.987	0.935
Solomon Islands	Male	0.046	1	1	1	0.303	1	1	1	1	1	0.717	0.161	0.981	0.892
Somalia	Both	< 0.001	0.989	0.614	0.815	< 0.001	1	0.988	0.953	0.173	0.067	0.019	< 0.001	0.16	0.012
Somalia	Female	< 0.001	1	0.994	0.942	< 0.001	1	0.985	0.999	0.293	0.243	0.085	< 0.001	0.242	0.027
Somalia	Male	< 0.001	1	0.938	0.999	< 0.001	1	0.985	1	0.864	0.465	0.055	< 0.001	0.707	0.292
South Africa	Both	< 0.001	< 0.001	< 0.001	< 0.001	< 0.001	< 0.001	0.003	< 0.001	< 0.001	< 0.001	0.27	< 0.001	< 0.001	< 0.001
South Africa	Female	< 0.001	< 0.001	< 0.001	< 0.001	< 0.001	< 0.001	0.073	< 0.001	0.007	< 0.001	0.005	< 0.001	< 0.001	0.003
South Africa	Male	< 0.001	< 0.001	< 0.001	< 0.001	< 0.001	0.54	0.029	< 0.001	< 0.001	< 0.001	0.091	< 0.001	< 0.001	< 0.001
South Sudan	Both	< 0.001	1	0.266	0.913	< 0.001	1	0.933	0.999	0.092	0.001	< 0.001	< 0.001	0.321	0.006
South Sudan	Female	< 0.001	1	0.978	0.974	< 0.001	1	0.97	1	0.239	0.066	0.013	< 0.001	0.371	0.02

South Sudan	Male	< 0.001	1	0.856	0.962	0.013	1	0.966	1	0.581	0.185	0.021	< 0.001	0.505	0.104
Spain	Both	< 0.001	0.35	< 0.001	0.081	< 0.001	0.887	0.006	0.197	< 0.001	< 0.001	< 0.001	< 0.001	0.392	< 0.001
Spain	Female	< 0.001	0.094	< 0.001	0.079	< 0.001	0.959	0.001	0.038	< 0.001	< 0.001	< 0.001	< 0.001	0.62	< 0.001
Spain	Male	< 0.001	0.016	< 0.001	< 0.001	< 0.001	0.568	0.046	0.006	< 0.001	< 0.001	< 0.001	< 0.001	0.043	< 0.001
Sri Lanka	Both	< 0.001	< 0.001	< 0.001	< 0.001	< 0.001	0.834	0.016	< 0.001	0.002	< 0.001	< 0.001	< 0.001	0.007	0.002
Sri Lanka	Female	< 0.001	0.053	< 0.001	0.09	< 0.001	0.99	0.104	0.02	0.03	< 0.001	0.009	< 0.001	0.106	0.008
Sri Lanka	Male	< 0.001	0.349	< 0.001	0.136	< 0.001	0.999	0.197	0.189	0.203	0.003	0.001	< 0.001	0.064	0.151
Sudan	Both	< 0.001	0.859	0.013	0.425	< 0.001	1	0.97	0.693	0.245	0.011	0.01	< 0.001	0.068	0.02
Sudan	Female	< 0.001	0.949	0.067	0.719	< 0.001	1	0.952	0.855	0.511	0.082	0.03	< 0.001	0.182	0.076
Sudan	Male	< 0.001	0.998	0.76	0.837	< 0.001	1	0.998	0.988	0.667	0.259	0.091	< 0.001	0.224	0.129
Suriname	Both	0.003	1	1	0.99	0.557	1	0.758	1	0.866	0.928	0.817	< 0.001	0.939	0.768
Suriname	Female	0.102	1	1	0.996	0.848	1	0.918	1	0.949	0.981	0.992	0.009	0.92	0.65
Suriname	Male	0.516	1	1	0.995	0.973	1	0.873	1	0.95	0.978	0.759	0.004	0.759	0.916

Sweden	Both	< 0.001	< 0.001	< 0.001	< 0.001	< 0.001	0.002	0.001	< 0.001	< 0.001	< 0.001	< 0.001	< 0.001	< 0.001	< 0.001
Sweden	Female	< 0.001	< 0.001	< 0.001	< 0.001	< 0.001	0.319	0.003	< 0.001	< 0.001	< 0.001	0.093	< 0.001	0.04	< 0.001
Sweden	Male	< 0.001	< 0.001	< 0.001	< 0.001	< 0.001	< 0.001	0.031	< 0.001	< 0.001	< 0.001	< 0.001	< 0.001	< 0.001	< 0.001
Switzerland	Both	< 0.001	0.203	0.003	0.912	< 0.001	0.159	0.025	0.096	< 0.001	< 0.001	0.16	< 0.001	0.819	< 0.001
Switzerland	Female	< 0.001	0.699	0.012	0.931	< 0.001	0.769	0.353	0.533	< 0.001	< 0.001	0.166	< 0.001	0.854	< 0.001
Switzerland	Male	< 0.001	0.758	0.076	0.932	< 0.001	0.704	0.043	0.565	< 0.001	< 0.001	0.152	< 0.001	0.908	< 0.001
Syrian Arab Republic	Both	< 0.001	0.683	0.612	0.056	< 0.001	1	0.48	0.477	0.374	0.433	0.65	< 0.001	0.042	0.26
Syrian Arab Republic	Female	< 0.001	0.756	0.793	0.077	< 0.001	0.974	0.679	0.562	0.793	0.879	0.869	< 0.001	0.185	0.808
Syrian Arab Republic	Male	< 0.001	0.858	0.721	0.129	< 0.001	1	0.765	0.705	0.703	0.613	0.358	< 0.001	0.099	0.368
Taiwan (Province of China)	Both	< 0.001	< 0.001	< 0.001	< 0.001	< 0.001	0.043	0.377	< 0.001	0.538	0.142	0.024	< 0.001	0.056	0.868

Taiwan (Province of China)	Female	< 0.001	< 0.001	< 0.001	< 0.001	< 0.001	0.557	0.285	< 0.001	0.415	< 0.001	< 0.001	< 0.001	0.065	0.476
Taiwan (Province of China)	Male	< 0.001	0.127	0.118	0.008	< 0.001	0.636	0.956	0.056	0.959	0.703	0.167	< 0.001	0.264	0.663
Tajikistan	Both	< 0.001	0.778	0.002	0.578	< 0.001	1	0.6	0.594	0.76	0.072	0.006	< 0.001	0.185	0.95
Tajikistan	Female	< 0.001	0.998	0.65	1	< 0.001	0.999	0.305	0.99	0.348	0.259	0.15	< 0.001	0.811	0.342
Tajikistan	Male	< 0.001	< 0.001	< 0.001	< 0.001	< 0.001	0.994	0.923	< 0.001	0.361	< 0.001	< 0.001	< 0.001	< 0.001	0.051
Thailand	Both	< 0.001	< 0.001	< 0.001	0.001	< 0.001	0.524	0.003	< 0.001	0.006	< 0.001	< 0.001	< 0.001	0.011	0.234
Thailand	Female	< 0.001	0.002	< 0.001	0.008	< 0.001	0.818	0.103	0.001	0.018	< 0.001	< 0.001	< 0.001	0.08	0.008
Thailand	Male	< 0.001	0.937	0.47	0.813	< 0.001	0.998	0.02	0.9	0.004	0.007	0.148	< 0.001	0.252	0.154
Timor-Leste	Both	< 0.001	1	0.998	0.995	0.723	1	0.983	1	0.981	0.475	0.067	< 0.001	0.791	0.552
Timor-Leste	Female	0.013	1	1	0.999	0.938	1	0.996	1	0.998	0.824	0.188	< 0.001	0.922	0.758
Timor-Leste	Male	0.01	1	1	0.999	0.975	1	0.992	1	0.991	0.803	0.201	< 0.001	0.772	0.618
Togo	Both	< 0.001	1	1	0.994	< 0.001	1	1	1	0.999	0.903	0.265	< 0.001	0.862	0.792

Togo	Female	< 0.001	1	1	0.998	0.004	1	0.999	1	0.982	0.98	0.702	< 0.001	0.846	0.525
Togo	Male	< 0.001	1	1	1	0.042	1	1	1	0.999	0.9	0.206	< 0.001	0.9	0.751
Tokelau	Both	1	1	1	1	1	1	1	1	1	1	0.977	0.805	0.992	0.999
Tokelau	Female	1	1	1	1	1	1	1	1	1	1	0.974	0.87	0.994	0.991
Tokelau	Male	1	1	1	1	1	1	1	1	1	1	0.983	0.843	0.997	0.993
Tonga	Both	0.902	1	1	1	1	1	0.998	1	0.999	0.999	0.746	0.083	0.985	0.84
Tonga	Female	0.989	1	1	1	0.995	1	1	1	1	1	0.865	0.373	0.987	0.998
Tonga	Male	0.936	1	1	1	0.999	1	0.997	1	0.998	0.999	0.772	0.117	0.974	0.765
Trinidad and Tobago	Both	< 0.001	1	1	1	0.005	1	0.824	1	0.913	0.896	0.458	< 0.001	0.78	0.748
Trinidad and Tobago	Female	< 0.001	1	1	0.999	0.006	1	0.899	1	0.949	0.972	0.763	< 0.001	0.659	0.67
Trinidad and Tobago	Male	< 0.001	1	1	1	0.314	1	0.944	1	0.984	0.973	0.496	< 0.001	0.933	0.994
Tunisia	Both	< 0.001	0.999	0.241	0.942	< 0.001	1	0.773	0.996	0.639	0.211	0.018	< 0.001	0.45	0.361
Tunisia	Female	< 0.001	1	0.994	0.998	< 0.001	1	0.791	1	0.721	0.645	0.179	< 0.001	0.738	0.457

Tunisia	Male	< 0.001	1	0.792	0.921	< 0.001	1	0.963	0.998	0.961	0.619	0.073	< 0.001	0.481	0.655
Turkey	Both	< 0.001	0.021	< 0.001	< 0.001	< 0.001	0.625	0.23	0.008	0.249	< 0.001	< 0.001	< 0.001	0.062	0.512
Turkey	Female	< 0.001	0.283	< 0.001	0.024	< 0.001	0.923	0.51	0.172	0.349	< 0.001	< 0.001	< 0.001	0.315	0.152
Turkey	Male	< 0.001	0.064	< 0.001	0.001	< 0.001	0.417	< 0.001	0.044	< 0.001	< 0.001	< 0.001	< 0.001	0.027	0.116
Turkmenistan	Both	< 0.001	1	0.007	1	< 0.001	1	0.396	1	0.316	0.001	< 0.001	< 0.001	0.798	0.142
Turkmenistan	Female	< 0.001	1	0.607	0.999	0.003	1	0.745	1	0.689	0.246	0.025	< 0.001	0.876	0.287
Turkmenistan	Male	< 0.001	1	0.433	1	< 0.001	1	0.612	1	0.619	0.024	0.001	< 0.001	0.783	0.296
Tuvalu	Both	1	1	1	1	1	1	1	1	1	1	0.991	0.615	0.974	0.992
Tuvalu	Female	1	1	1	1	1	1	1	1	1	1	0.99	0.713	0.978	0.993
Tuvalu	Male	1	1	1	1	1	1	1	1	1	1	0.99	0.722	0.99	0.985
Uganda	Both	< 0.001	0.416	< 0.001	0.191	< 0.001	1	0.864	0.23	0.555	0.007	0.002	< 0.001	0.022	0.161
Uganda	Female	< 0.001	0.991	0.018	0.842	< 0.001	1	0.944	0.958	0.598	0.027	0.008	< 0.001	0.119	0.131
Uganda	Male	< 0.001	0.994	0.94	0.765	< 0.001	1	0.9	0.971	0.96	0.602	0.1	< 0.001	0.229	0.912
Ukraine	Both	< 0.001	< 0.001	< 0.001	< 0.001	< 0.001	< 0.001	< 0.001	< 0.001	< 0.001	< 0.001	< 0.001	< 0.001	< 0.001	< 0.001

Ukraine	Female	< 0.001	< 0.001	< 0.001	0.042	< 0.001	< 0.001	< 0.001	< 0.001	< 0.001	< 0.001	< 0.001	< 0.001	0.013	< 0.001
Ukraine	Male	< 0.001	< 0.001	< 0.001	0.007	< 0.001	0.211	< 0.001	< 0.001	< 0.001	< 0.001	< 0.001	< 0.001	< 0.001	0.001
United Arab Emirates	Both	< 0.001	0.942	0.96	0.281	< 0.001	1	0.998	0.846	0.287	0.387	0.887	< 0.001	0.729	0.04
United Arab Emirates	Female	< 0.001	1	1	0.839	0.081	1	0.823	0.998	0.161	0.244	0.976	0.03	0.82	0.046
United Arab Emirates	Male	< 0.001	0.999	1	0.77	< 0.001	1	0.979	0.996	0.878	0.94	0.949	< 0.001	0.855	0.294
United Kingdom	Both	< 0.001	< 0.001	< 0.001	0.024	< 0.001	< 0.001	0.416	< 0.001	< 0.001	< 0.001	< 0.001	< 0.001	0.112	< 0.001
United Kingdom	Female	< 0.001	< 0.001	< 0.001	0.077	< 0.001	< 0.001	0.171	< 0.001	< 0.001	< 0.001	0.037	< 0.001	0.599	< 0.001
United Kingdom	Male	< 0.001	< 0.001	< 0.001	0.007	< 0.001	< 0.001	0.741	< 0.001	< 0.001	< 0.001	0.004	< 0.001	0.002	< 0.001
United Republic of Tanzania	Both	< 0.001	0.646	0.222	0.254	< 0.001	1	0.106	0.428	< 0.001	< 0.001	0.003	< 0.001	0.033	< 0.001
United Republic	Female	< 0.001	1	0.972	0.979	< 0.001	1	0.331	0.998	< 0.001	< 0.001	0.12	< 0.001	0.418	< 0.001

of Tanzania															
United Republic of Tanzania	Male	< 0.001	0.908	< 0.001	0.828	< 0.001	1	0.034	0.77	0.002	< 0.001	< 0.001	< 0.001	0.235	0.009
United States of America	Both	< 0.001	< 0.001	< 0.001	< 0.001	< 0.001	< 0.001	< 0.001	< 0.001	< 0.001	< 0.001	< 0.001	< 0.001	< 0.001	< 0.001
United States of America	Female	< 0.001	< 0.001	< 0.001	< 0.001	< 0.001	< 0.001	< 0.001	< 0.001	< 0.001	< 0.001	< 0.001	< 0.001	0.048	< 0.001
United States of America	Male	< 0.001	< 0.001	< 0.001	< 0.001	< 0.001	0.007	< 0.001	< 0.001	< 0.001	< 0.001	< 0.001	< 0.001	< 0.001	< 0.001
United States Virgin Islands	Both	0.969	1	1	1	0.995	1	0.911	1	0.965	0.941	0.406	0.127	0.694	0.969
United States Virgin Islands	Female	0.996	1	1	0.998	0.999	1	0.997	1	0.999	1	0.959	0.338	0.793	0.919
United States	Male	1	1	0.999	1	1	1	0.888	1	0.958	0.849	0.25	0.329	0.78	0.889

Virgin Islands															
Uruguay	Both	< 0.001	0.996	0.998	0.789	< 0.001	1	0.908	0.982	0.844	0.923	0.826	< 0.001	0.619	0.345
Uruguay	Female	< 0.001	0.999	0.999	0.933	0.002	1	0.92	0.99	0.797	0.892	0.834	< 0.001	0.695	0.26
Uruguay	Male	< 0.001	1	1	0.991	0.006	1	0.983	1	0.996	0.999	0.955	< 0.001	0.811	0.891
Uzbekistan	Both	< 0.001	< 0.001	< 0.001	< 0.001	< 0.001	< 0.001	< 0.001	< 0.001	< 0.001	< 0.001	< 0.001	< 0.001	< 0.001	0.003
Uzbekistan	Female	< 0.001	< 0.001	< 0.001	< 0.001	< 0.001	< 0.001	0.007	< 0.001	< 0.001	< 0.001	< 0.001	< 0.001	< 0.001	< 0.001
Uzbekistan	Male	< 0.001	< 0.001	< 0.001	< 0.001	< 0.001	0.141	0.119	< 0.001	0.197	< 0.001	< 0.001	< 0.001	< 0.001	0.391
Vanuatu	Both	0.811	1	1	1	0.999	1	0.997	1	1	0.988	0.462	0.07	0.855	0.996
Vanuatu	Female	0.794	1	1	1	0.844	1	0.998	1	1	0.998	0.634	0.198	0.855	0.998
Vanuatu	Male	0.715	1	1	1	0.944	1	0.999	1	1	0.997	0.59	0.189	0.938	0.997
Venezuela (Bolivarian Republic of)	Both	< 0.001	0.844	0.874	0.737	< 0.001	0.805	0.023	0.929	0.013	0.023	0.952	< 0.001	0.586	0.281
Venezuela (Bolivarian	Female	< 0.001	0.169	0.194	0.155	< 0.001	0.558	0.025	0.266	< 0.001	< 0.001	0.135	< 0.001	0.702	< 0.001

Republic of)															
Venezuela (Bolivarian Republic of)	Male	< 0.001	0.798	0.708	0.293	< 0.001	0.984	0.012	0.726	< 0.001	< 0.001	0.074	< 0.001	0.24	< 0.001
Viet Nam	Both	< 0.001	0.001	< 0.001	< 0.001	< 0.001	0.004	0.011	< 0.001	< 0.001	< 0.001	< 0.001	< 0.001	0.134	< 0.001
Viet Nam	Female	< 0.001	0.006	< 0.001	0.018	< 0.001	0.006	0.102	0.002	< 0.001	< 0.001	< 0.001	< 0.001	0.91	< 0.001
Viet Nam	Male	< 0.001	0.006	< 0.001	< 0.001	< 0.001	0.852	0.128	0.001	0.197	< 0.001	< 0.001	< 0.001	< 0.001	0.925
Yemen	Both	< 0.001	1	0.802	0.873	< 0.001	1	0.754	0.998	0.864	0.171	0.016	< 0.001	0.372	0.589
Yemen	Female	< 0.001	0.999	0.58	0.864	< 0.001	1	0.857	0.991	0.934	0.302	0.03	< 0.001	0.415	0.659
Yemen	Male	< 0.001	1	1	0.995	< 0.001	1	0.925	1	0.976	0.765	0.157	< 0.001	0.627	0.822
Zambia	Both	< 0.001	0.984	< 0.001	0.889	< 0.001	1	0.84	0.936	< 0.001	< 0.001	< 0.001	< 0.001	0.243	< 0.001
Zambia	Female	< 0.001	0.98	< 0.001	0.853	< 0.001	1	0.832	0.925	0.001	< 0.001	< 0.001	< 0.001	0.139	< 0.001
Zambia	Male	< 0.001	1	< 0.001	0.997	< 0.001	1	0.921	1	0.421	< 0.001	< 0.001	< 0.001	0.795	0.098

Zimbabwe	Both	< 0.001	1	0.98	0.799	< 0.001	1	0.899	0.997	0.589	0.325	0.05	< 0.001	0.876	0.152
Zimbabwe	Female	< 0.001	1	0.833	0.99	< 0.001	1	0.195	1	0.302	0.064	0.015	< 0.001	0.996	0.933
Zimbabwe	Male	< 0.001	1	1	0.999	< 0.001	1	0.464	1	0.062	0.082	0.246	< 0.001	0.596	0.009
