# Supplementary material for: Differences in global, regional, and national time trends in disability-adjusted life years for atrial fibrillation and flutter, 1990–2019: an age-period-cohort analysis from the 2019 global burden of disease study
Source: Front Cardiovasc Med. 2024 Aug 29;11:1401722. doi: 10.3389/fcvm.2024.1401722 (PMC11390633; doi:10.3389/fcvm.2024.1401722)
Supplement: Supplementary file 3 [file Datasheet1.pdf]

Figure S1.The local drifts of atrial fibrillation and flutter DALY in all age groups in global,1990-2019.Local drifts of atrial fibrillation and flutter DALY (estimates from age-period-cohort models) for 19 age groups (5−9 to 95 plus years), 1990−2019. The dots and shaded areas indicate the annual percentage change of DALY (% per year) and the corresponding 95% CIs.

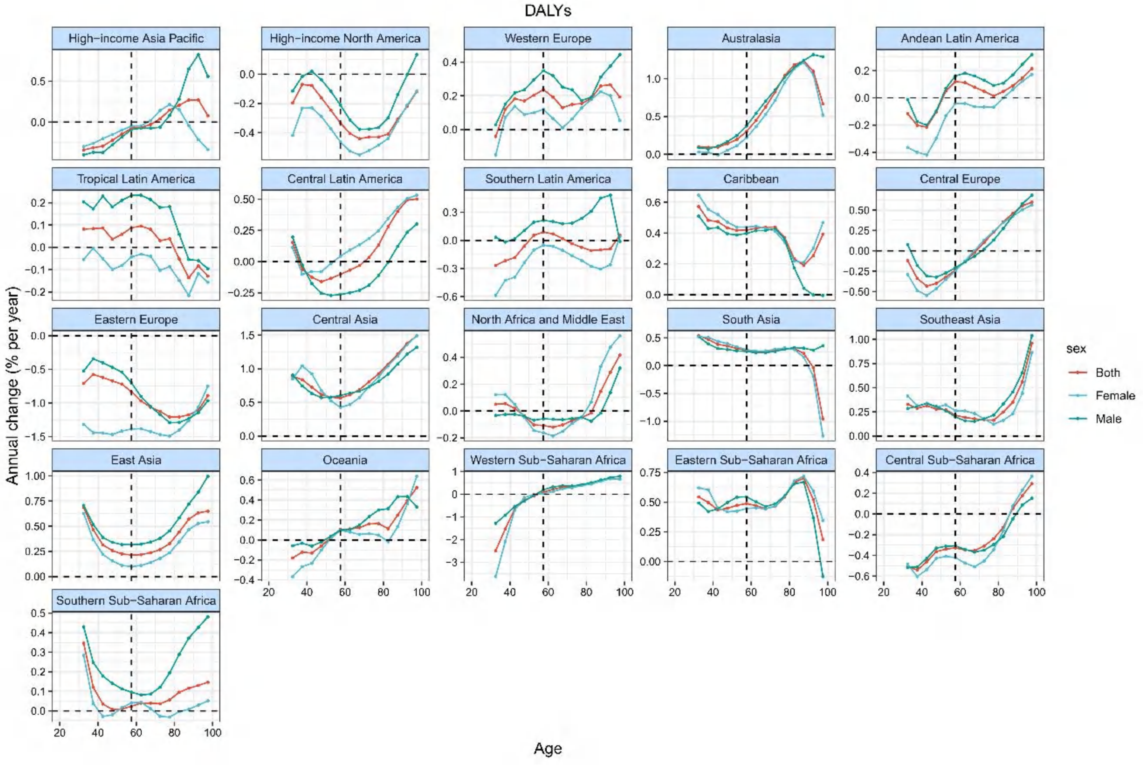

Figure S2.The local drifts of atrial fibrillation and flutter DALY in all age groups in high-SDI countries,1990-2019.Local drifts of atrial fibrillation and flutter DALY (estimates from age-period-cohort models) for 19 age groups (5−9 to 95 plus years), 1990−2019. The dots and shaded areas indicate the annual percentage change of DALY (% per year) and the corresponding 95% CIs.

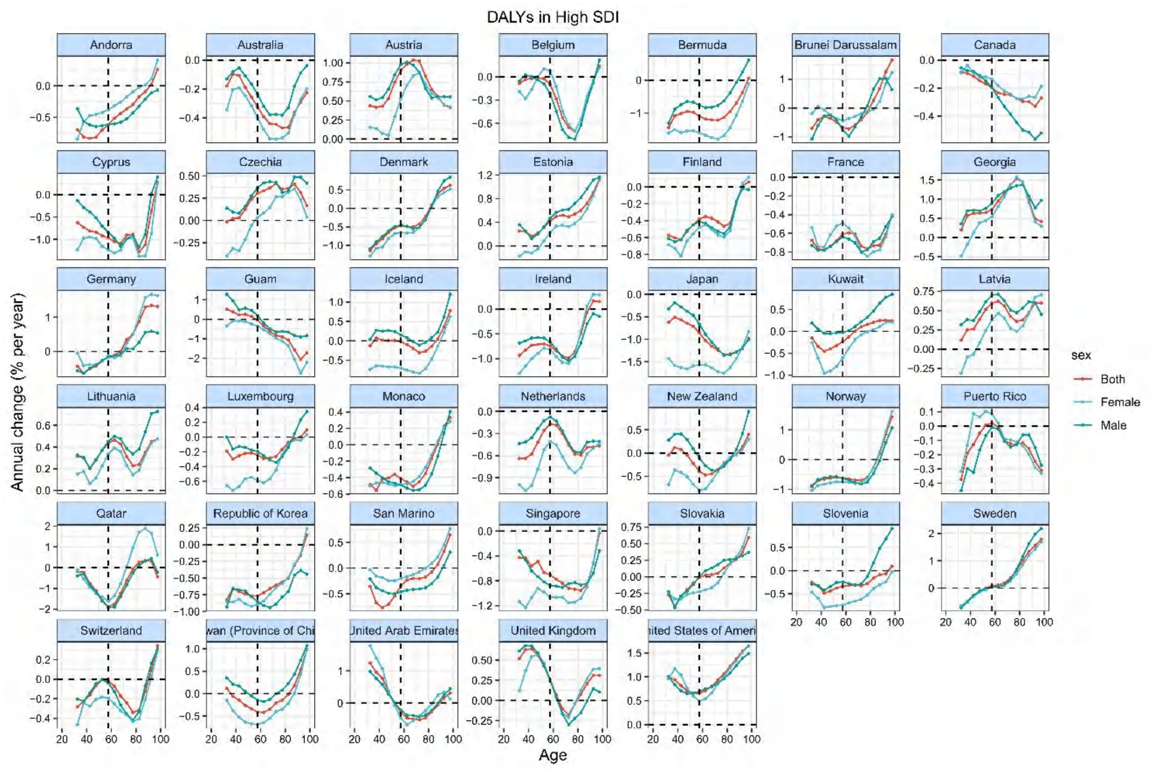

Figure S3.The local drifts of atrial fibrillation and flutter DALY in all age groups in high-middle SDI countries,1990-2019.Local drifts of atrial fibrillation and flutter DALY (estimates from age-period-cohort models) for 19 age groups (5−9 to 95 plus years), 1990−2019. The dots and shaded areas indicate the annual percentage change of DALY (% per year) and the corresponding 95% CIs.

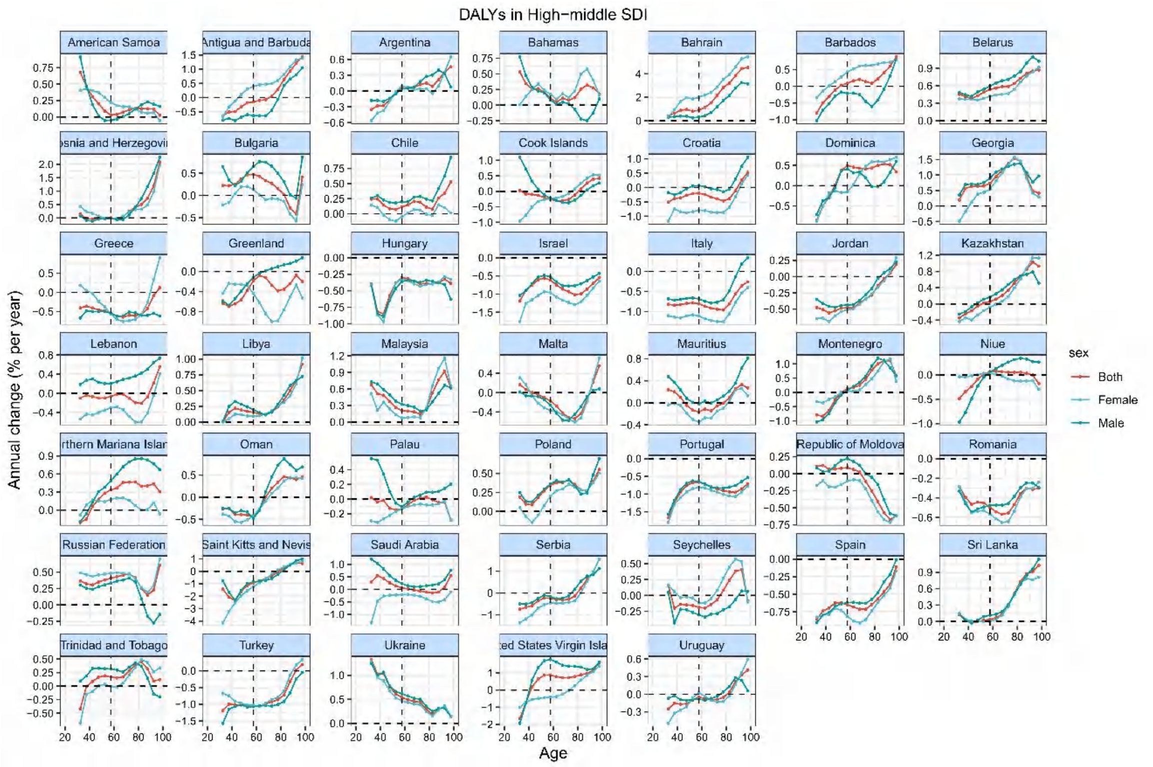

Figure S4.The local drifts of atrial fibrillation and flutter DALY in all age groups in middle-SDI countries,1990-2019.Local drifts of atrial fibrillation and flutter DALY (estimates from age-period-cohort models) for 19 age groups (5−9 to 95 plus years), 1990−2019. The dots and shaded areas indicate the annual percentage change of DALY (% per year) and the corresponding 95% CIs.

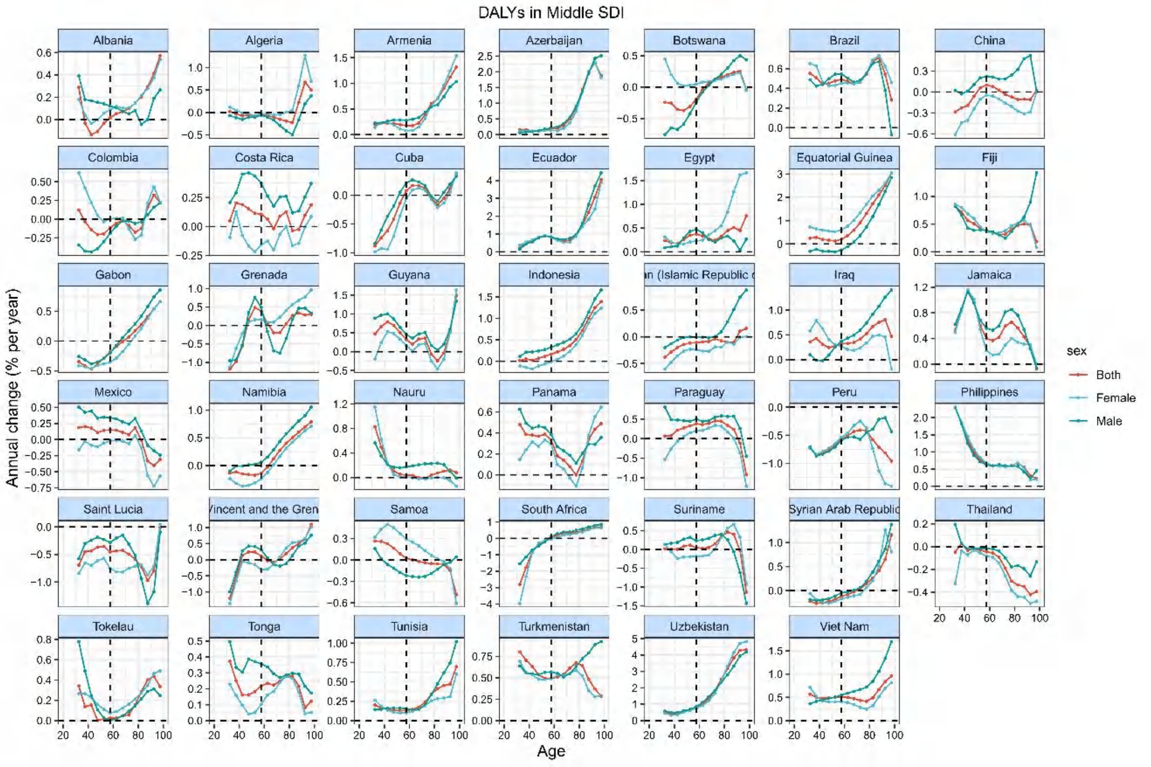

Figure S5.The local drifts of atrial fibrillation and flutter DALY in all age groups in low-middle SDI countries,1990-2019.Local drifts of atrial fibrillation and flutter DALY (estimates from age-period-cohort models) for 19 age groups (5−9 to 95 plus years), 1990−2019. The dots and shaded areas indicate the annual percentage change of DALY (% per year) and the corresponding 95% CIs.

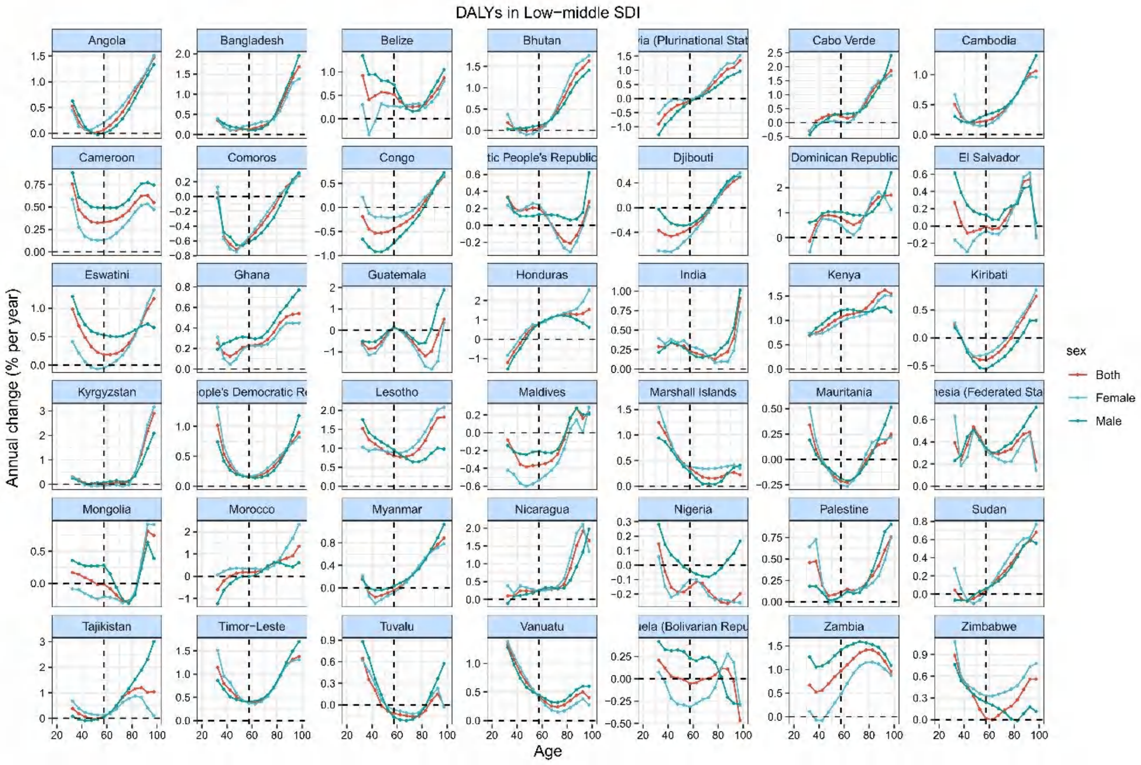

Figure S6.The local drifts of atrial fibrillation and flutter DALY in all age groups in low-SDI countries,1990-2019.Local drifts of atrial fibrillation and flutter DALY (estimates from age-period-cohort models) for 19 age groups (5−9 to 95 plus years), 1990−2019. The dots and shaded areas indicate the annual percentage change of DALY (% per year) and the corresponding 95% CIs.

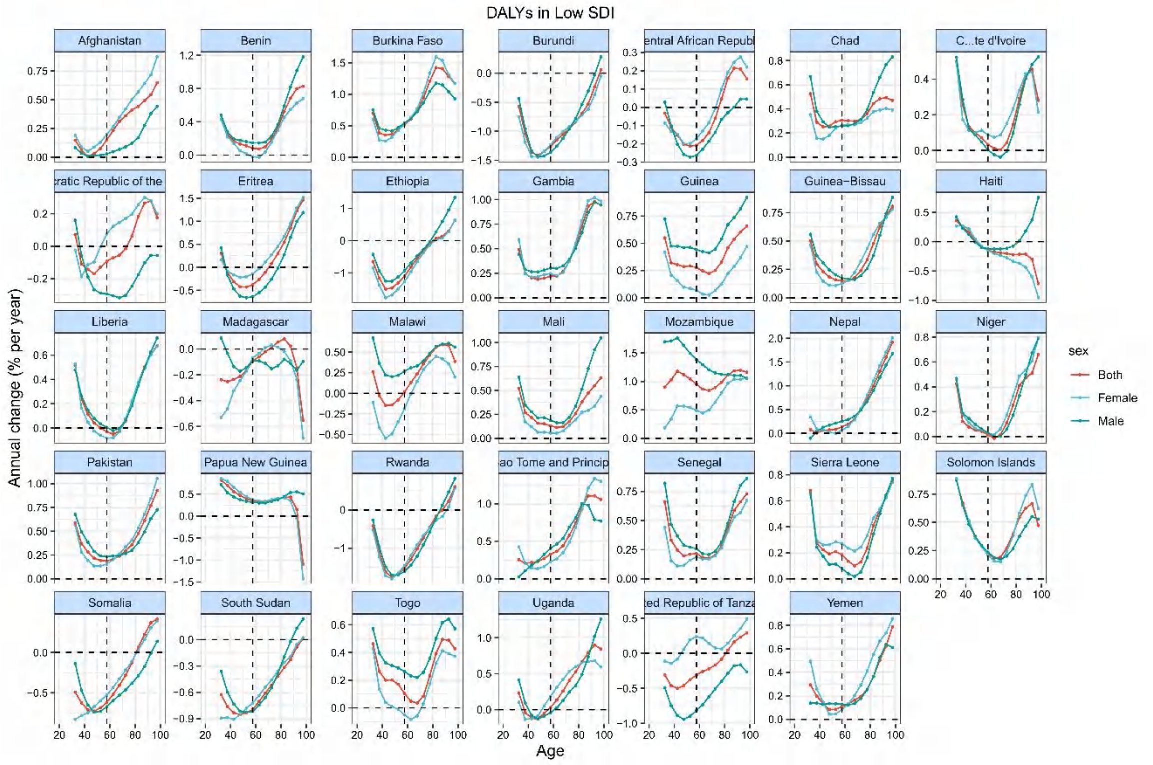

Figure S7.Age distribution of absolute cases of atrial fibrillation and flutter DALY in global,1990-2019.

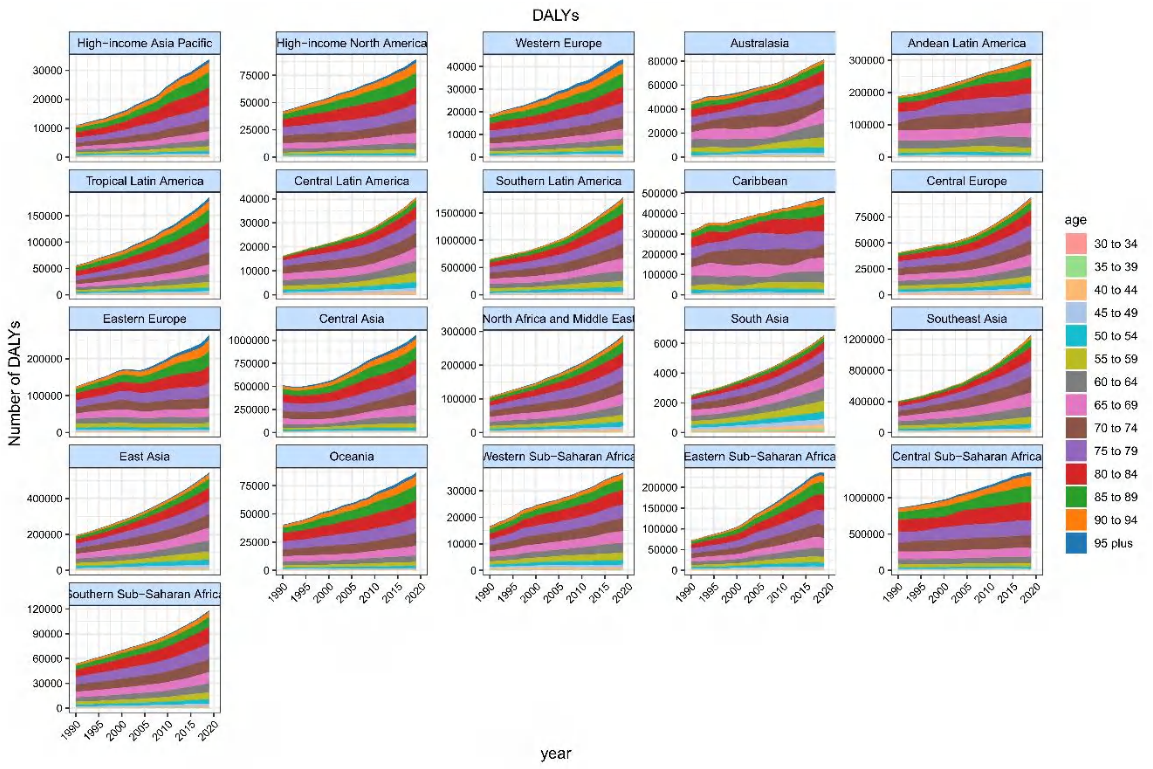

Figure S8. Age distribution of absolute cases of atrial fibrillation and flutter DALY in high-SDI countries, 1990–2019.

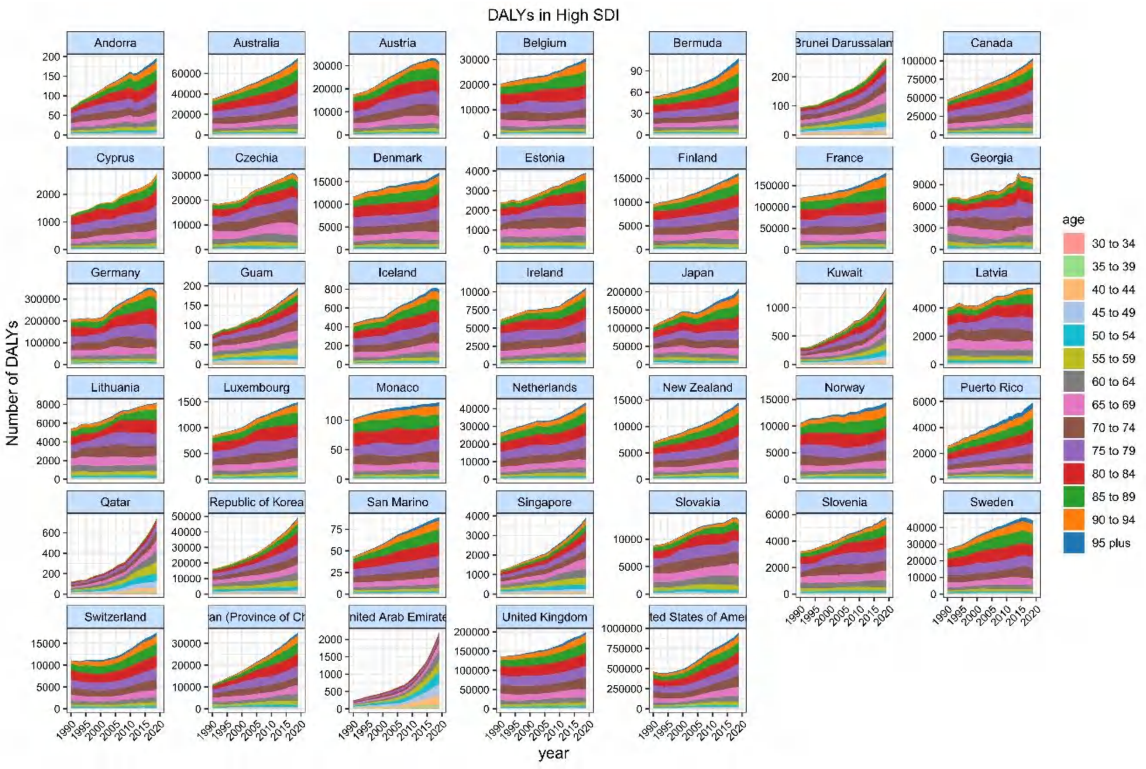

Figure S9. Age distribution of absolute cases of atrial fibrillation and flutter DALY in high-middle SDI countries, 1990–2019.

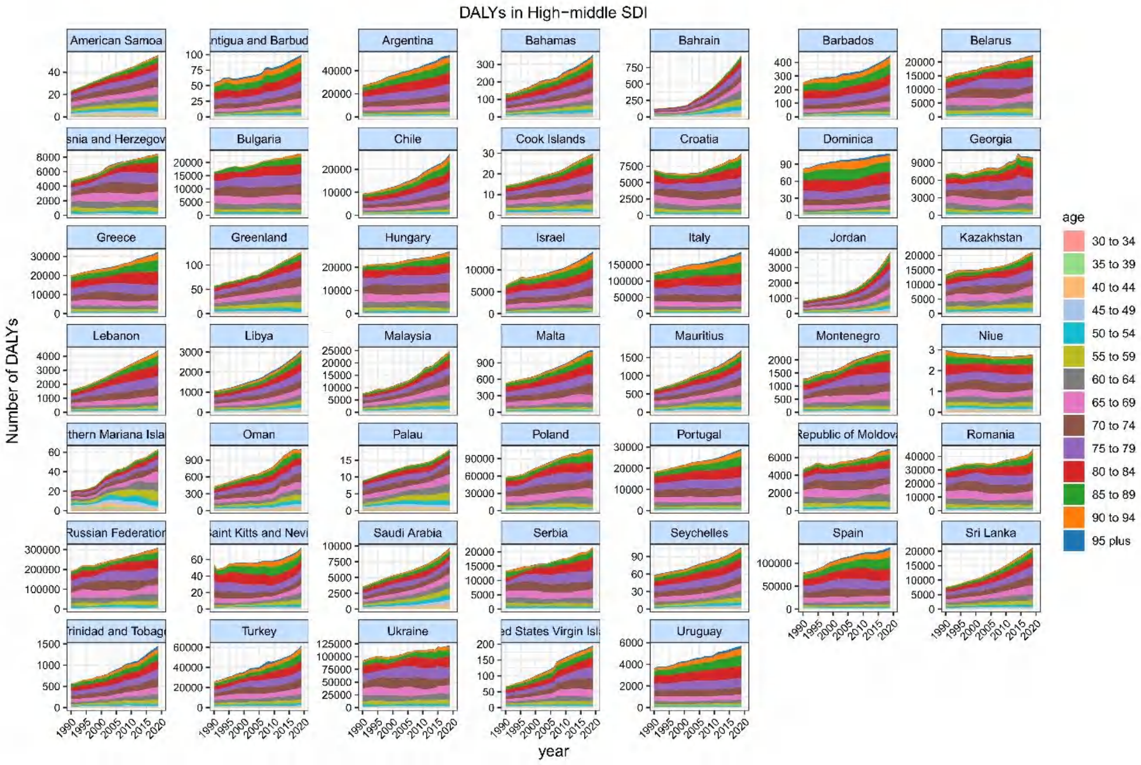

Figure S10. Age distribution of absolute cases of atrial fibrillation and flutter DALY in middle-SDI countries, 1990–2019.

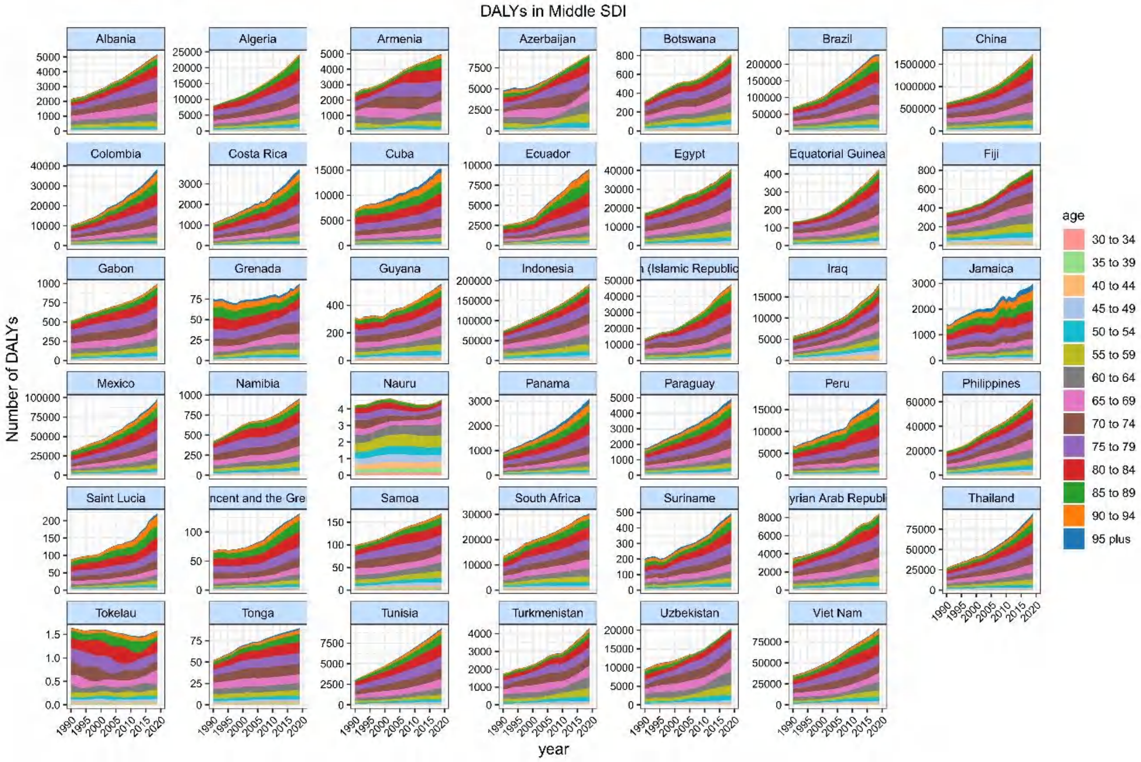

Figure S11. Age distribution of absolute cases of atrial fibrillation and flutter DALY in low-middle SDI countries, 1990–2019.

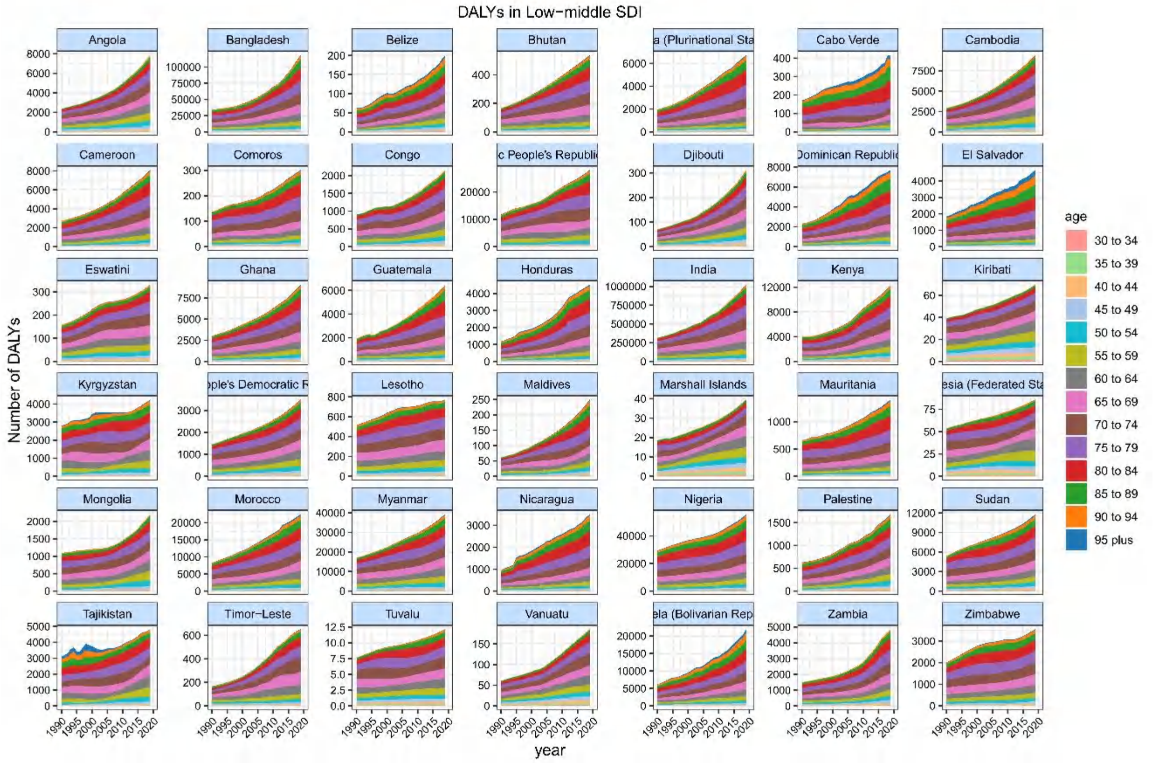

Figure S12. Age distribution of absolute cases of atrial fibrillation and flutter DALY in low-SDI countries, 1990–2019.

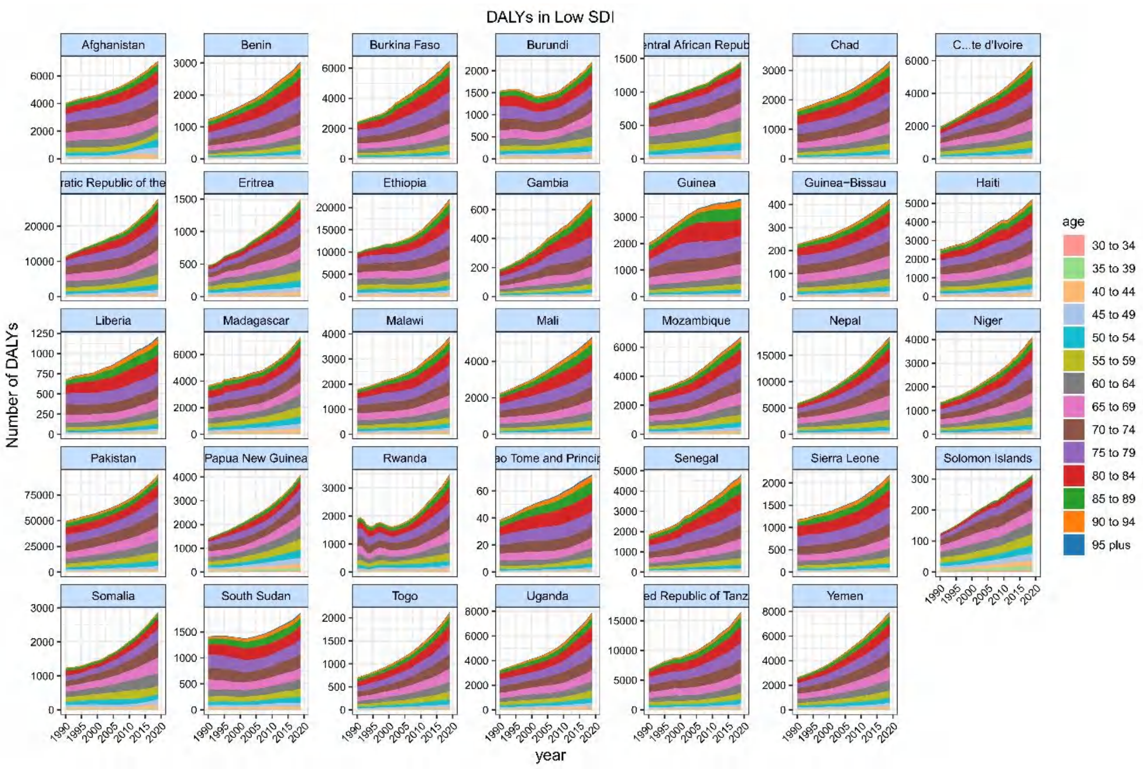

Figure S13. Age distribution of the relative proportion of atrial fibrillation and flutter DALY in the global, 1990-2019.

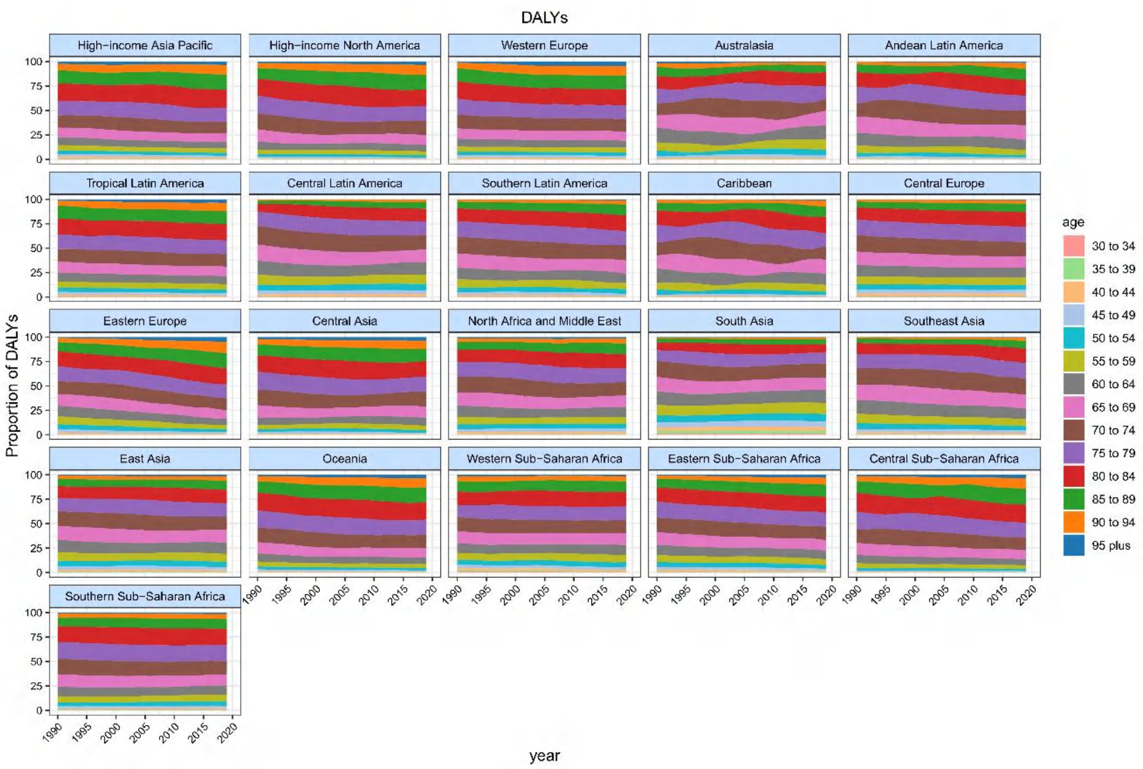

Figure S14. Age distribution of the relative proportion of atrial fibrillation and flutter DALY in high-SDI countries, 1990-2019.

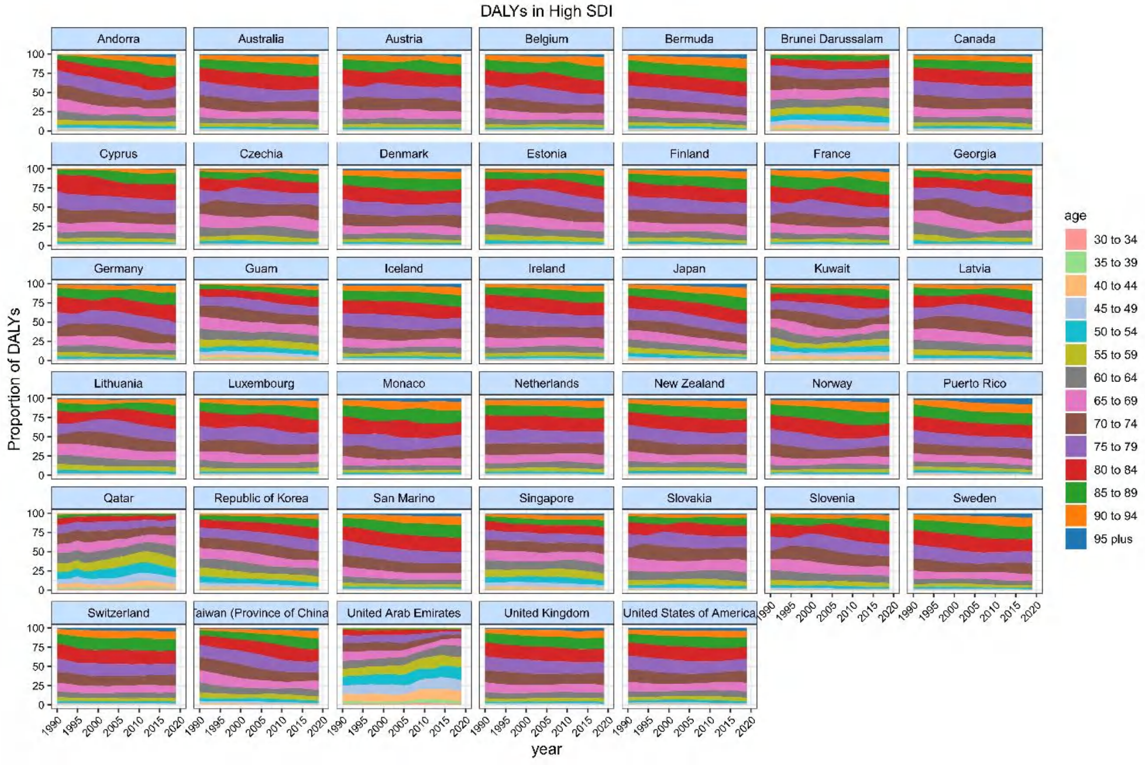

Figure S15. Age distribution of the relative proportion of atrial fibrillation and flutter DALY in high-middle SDI countries, 1990-2019.

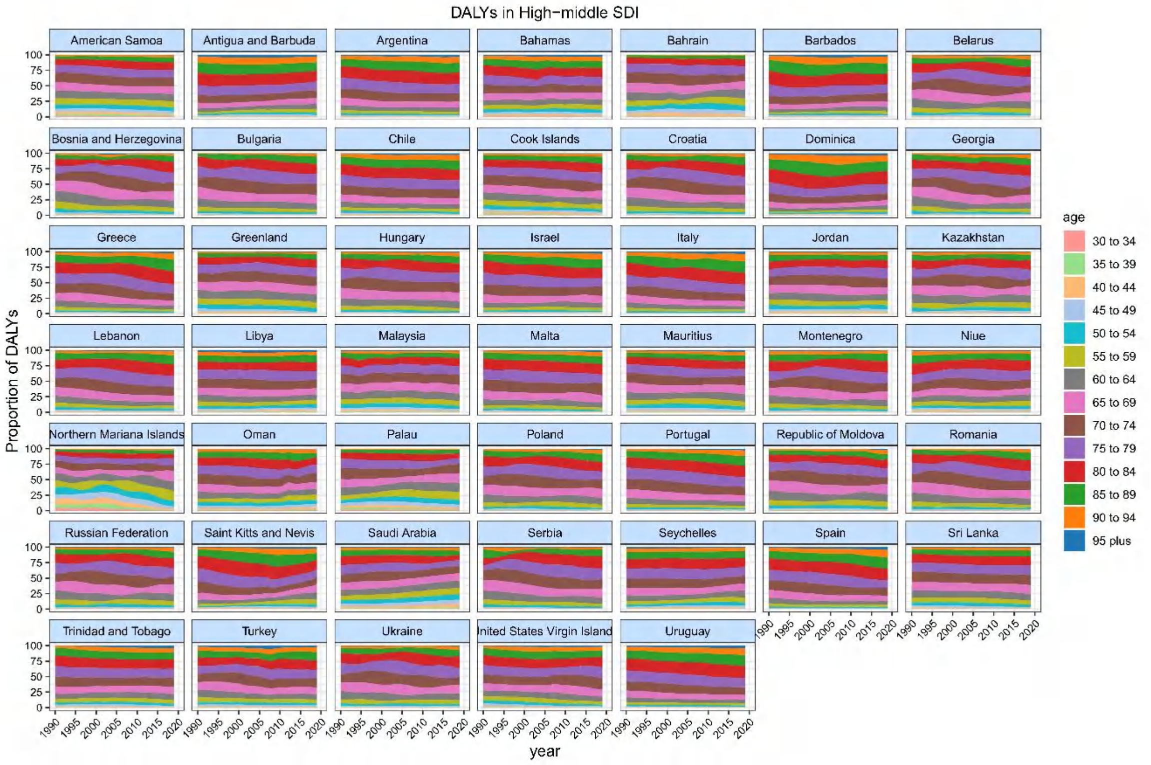

Figure S16.Age distribution of the relative proportion of atrial fibrillation and flutter DALY in middle-SDI countries, 1990-2019.

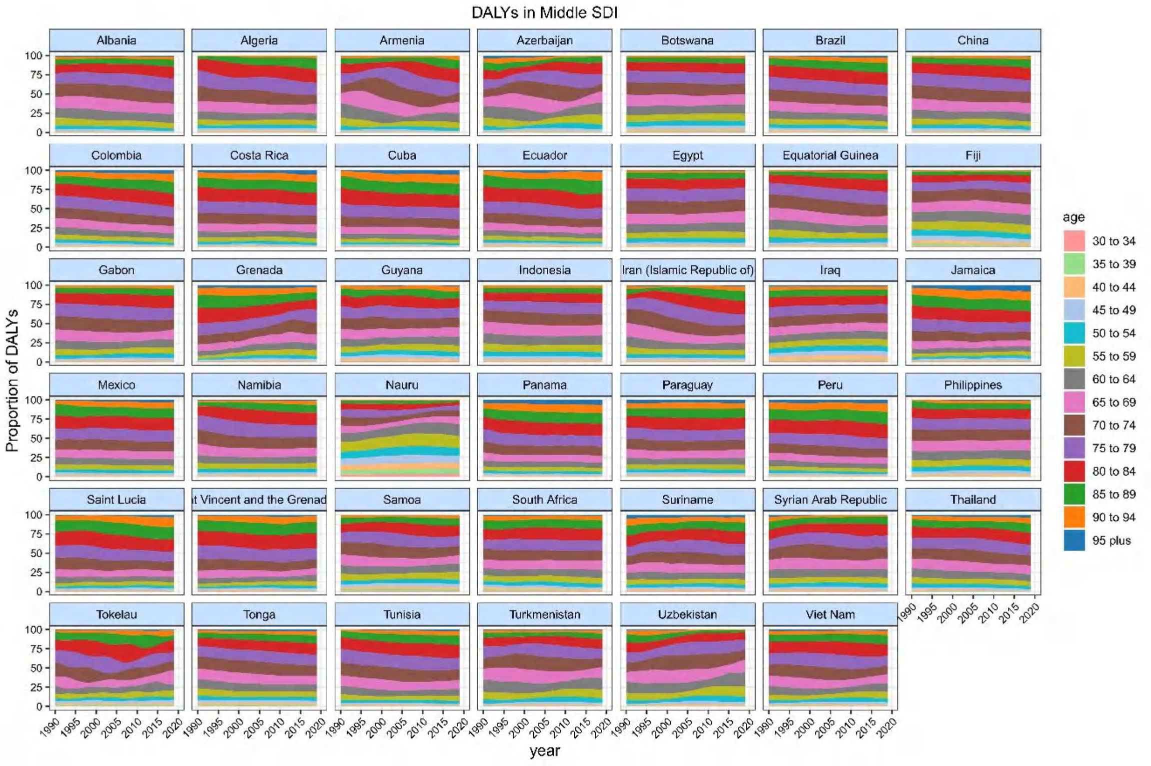

Figure S17. Age distribution of the relative proportion of atrial fibrillation and flutter DALY in low-middle SDI countries, 1990-2019.

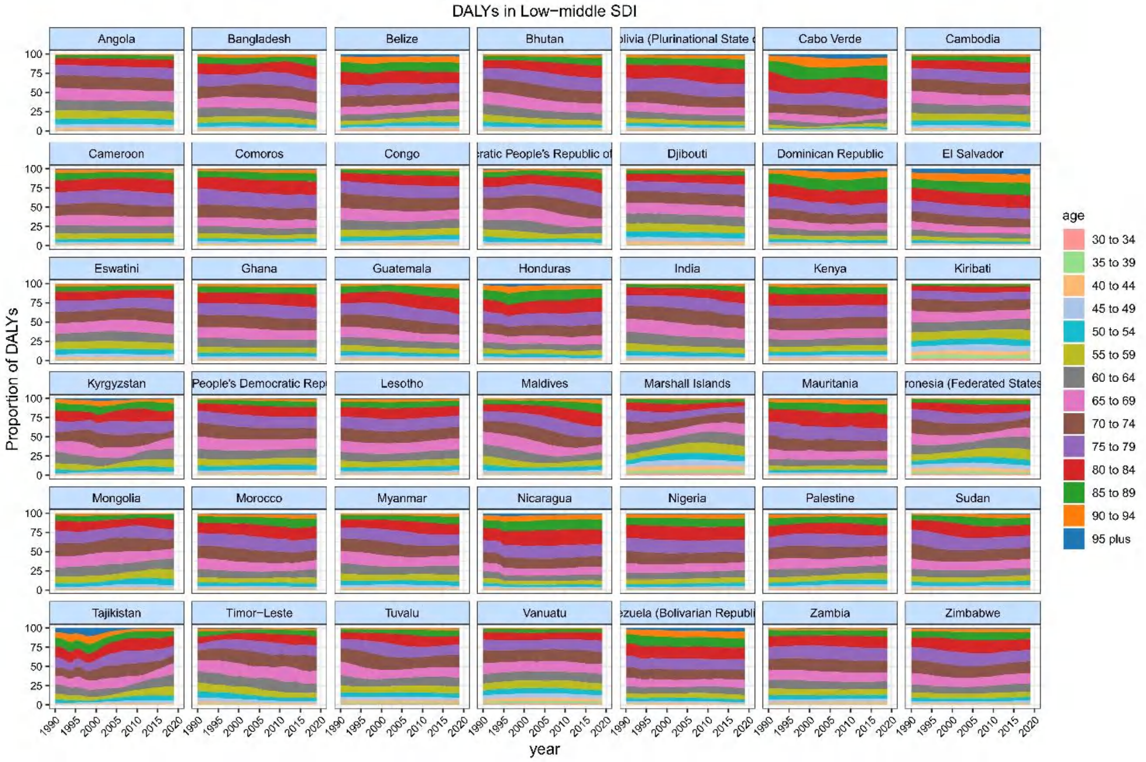

Figure S18. Age distribution of the relative proportion of atrial fibrillation and flutter DALY in low-SDI countries, 1990-2019.

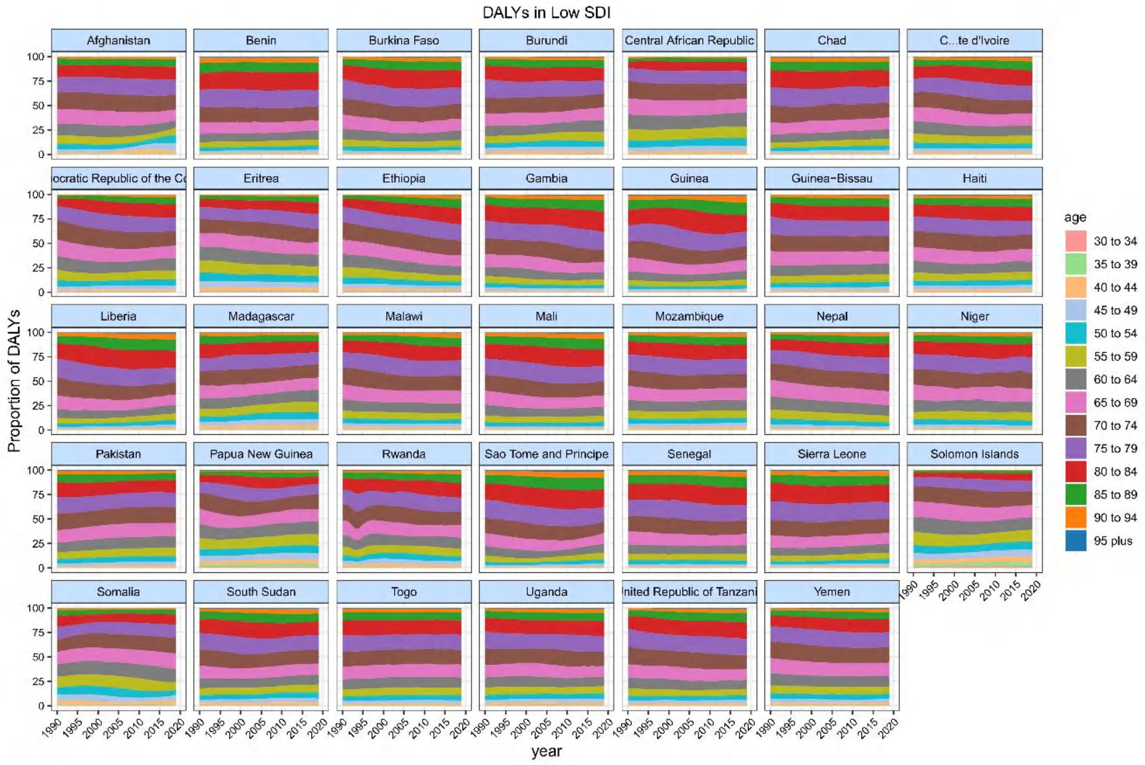

Figure S19. Age effects on atrial fibrillation and flutter DALY in global, 1990–2019 Age effects are shown by the fitted longitudinal age curves of incidence (per 100,000 person-years) adjusted for period deviations.

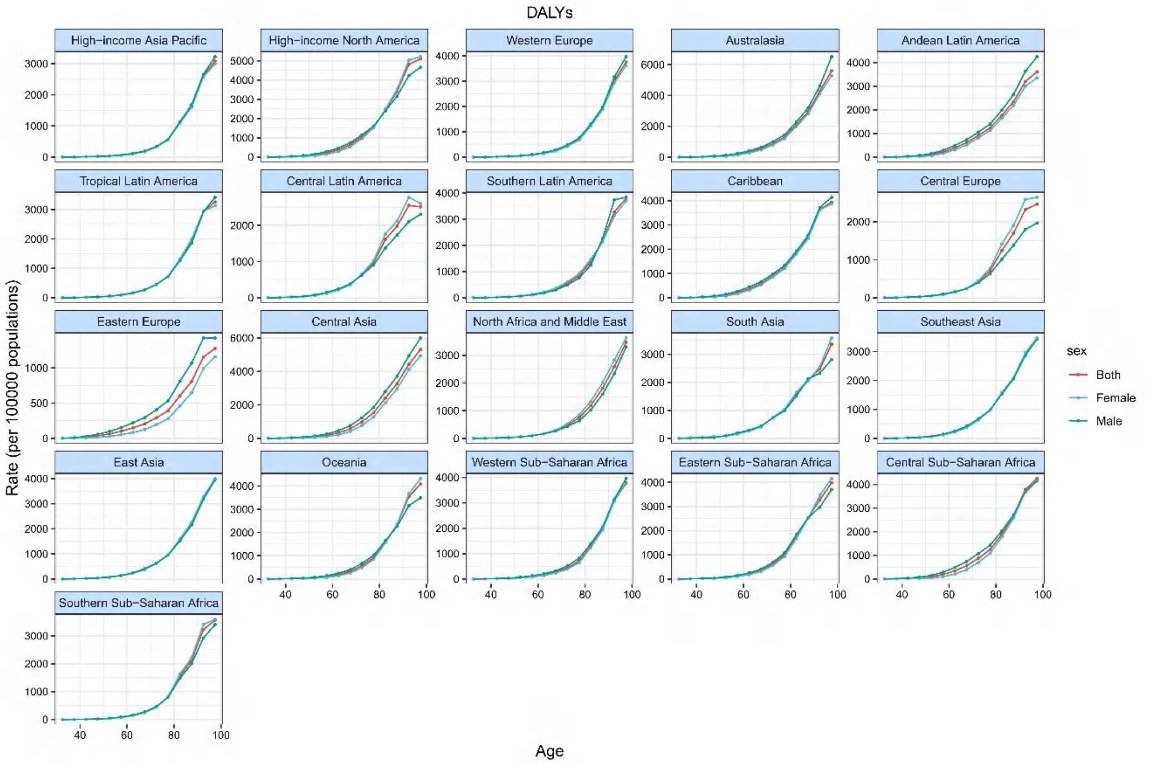

Figure S20. Age effects on atrial fibrillation and flutter DALY in high-SDI countries Age effects are shown by the fitted longitudinal age curves of incidence (per 100,000 person-years) adjusted for period deviations.

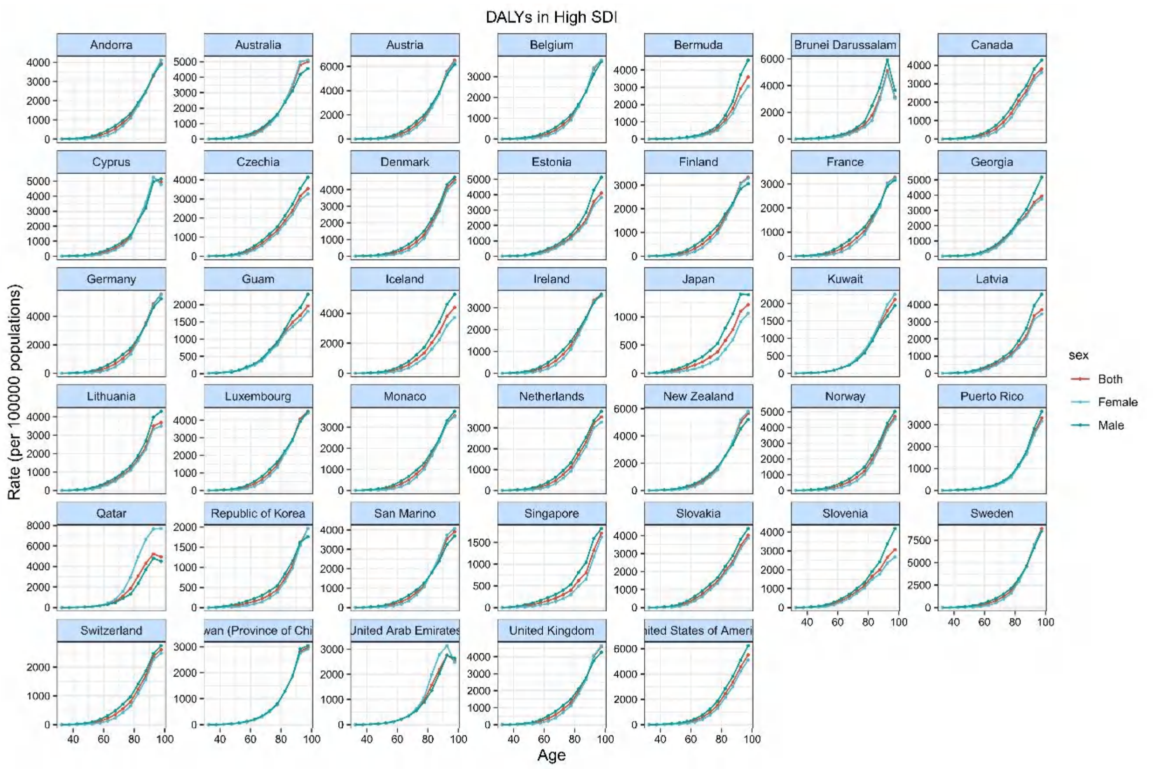

Figure S21. Age effects on atrial fibrillation and flutter DALY in high-middle SDI countries Age effects are shown by the fitted longitudinal age curves of incidence (per 100,000 person-years) adjusted for period deviations.

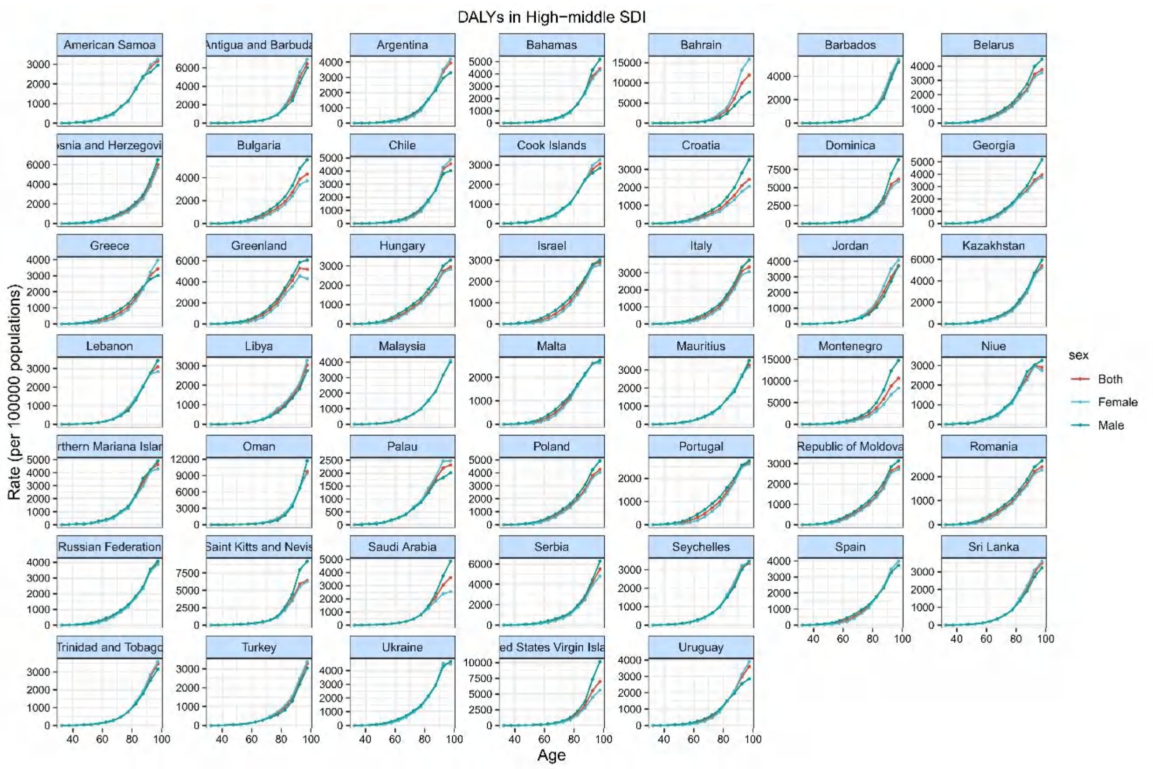

Figure S22. Age effects on atrial fibrillation and flutter DALY in middle-SDI countries Age effects are shown by the fitted longitudinal age curves of incidence (per 100,000 person-years) adjusted for period deviations.

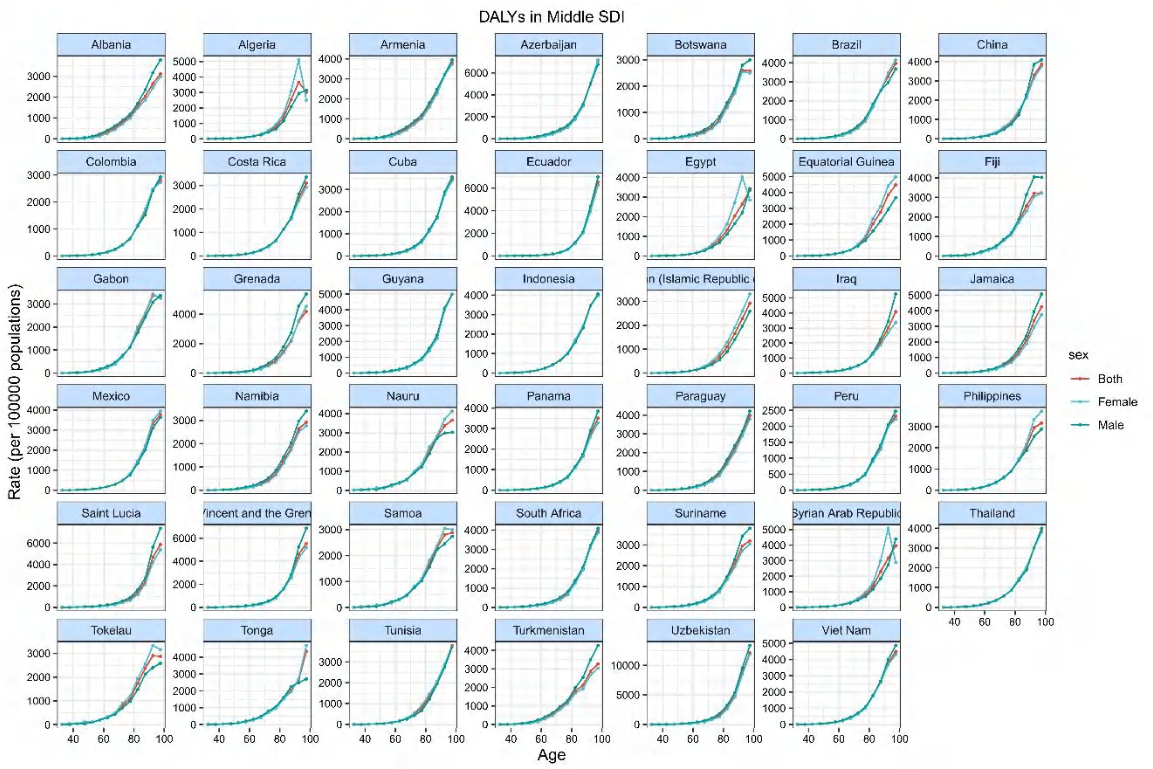

Figure S23. Age effects on atrial fibrillation and flutter DALY in low-middle SDI countries Age effects are shown by the fitted longitudinal age curves of incidence (per 100,000 person-years) adjusted for period deviations.

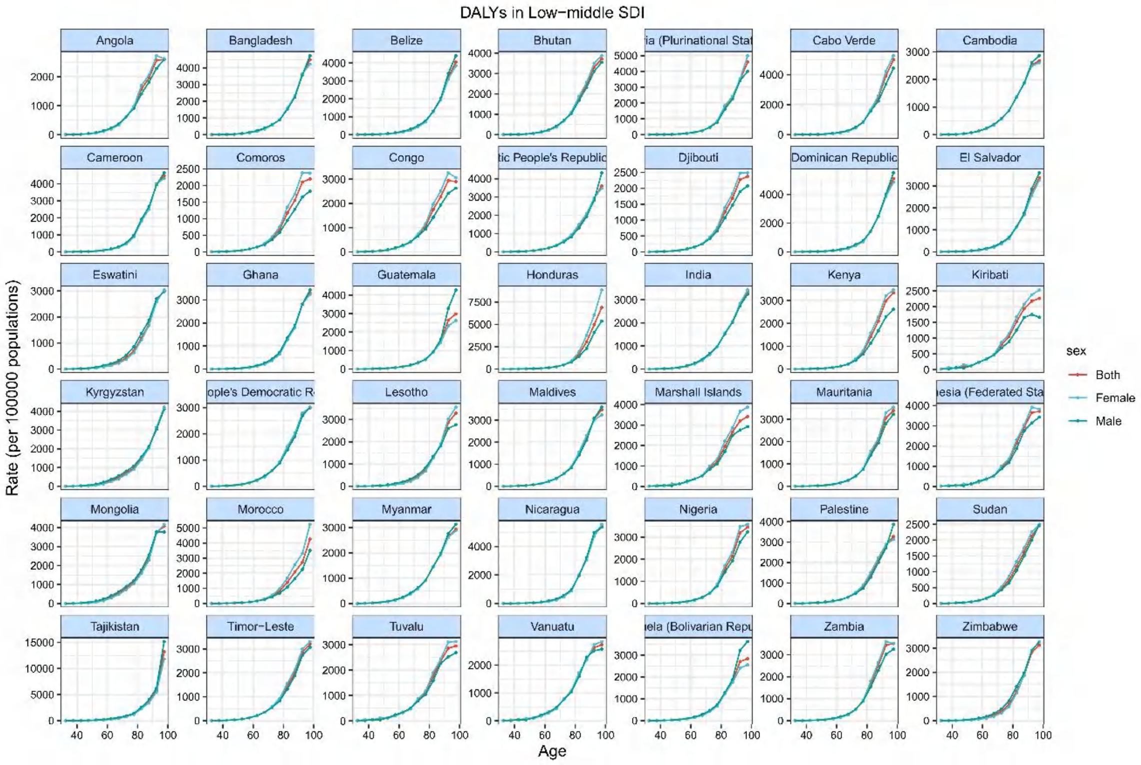

Figure S24. Age effects on atrial fibrillation and flutter DALY in low-SDI countries Age effects are shown by the fitted longitudinal age curves of incidence (per 100,000 person-years) adjusted for period deviations.

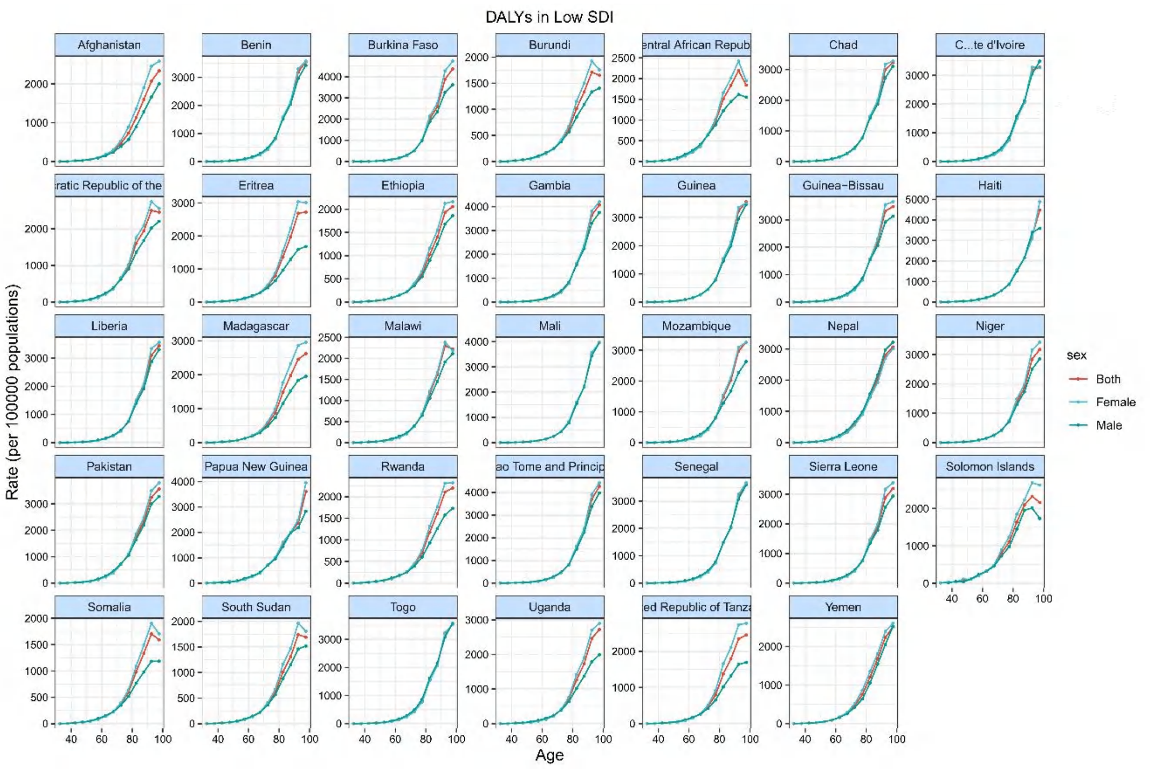

Figure S25. Period effects on atrial fibrillation and flutter DALY in global, 1990-2019 Period effects are shown by the relative risk of DALY (mortality rate ratio) and computed as the ratio of age-specific rates from 1990−1994 to 2015−2019 (2000−2005 as the referent period). The dots and shaded areas denote DALY rates or rate ratios and their corresponding 95% Cis.

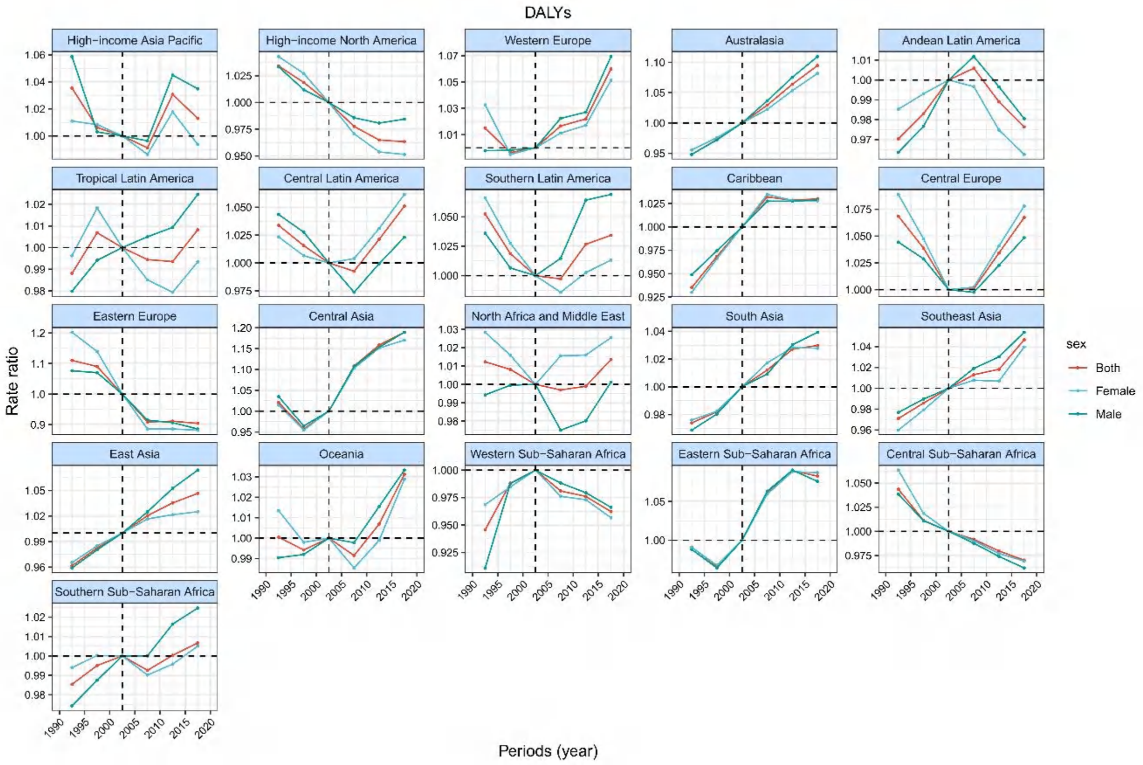

Figure S26. Period effects on atrial fibrillation and flutter DALY in high-SDI countries Period effects are shown by the relative risk of DALY (mortality rate ratio) and computed as the ratio of age-specific rates from 1990−1994 to 2015−2019 (2000−2005 as the referent period). The dots and shaded areas denote DALY rates or rate ratios and their corresponding 95% Cis.

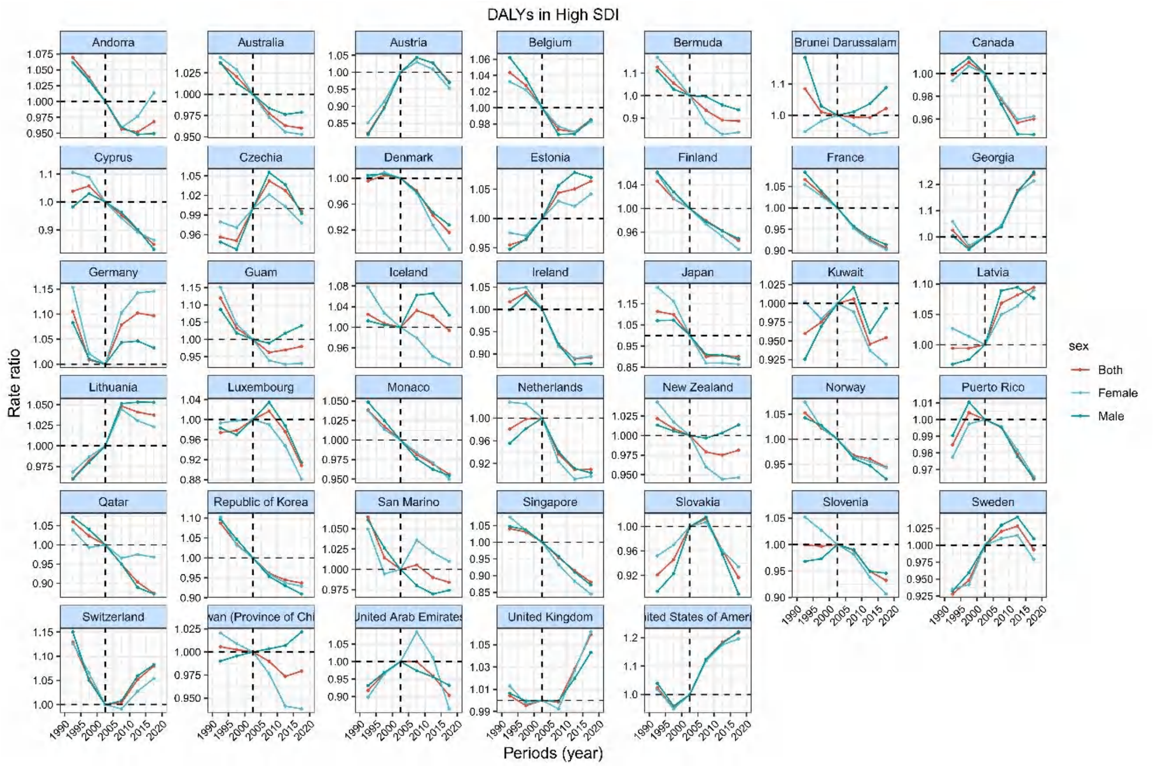

Figure S27. Period effects on atrial fibrillation and flutter DALY in high-middle SDI countries Period effects are shown by the relative risk of DALY (mortality rate ratio) and computed as the ratio of age-specific rates from 1990−1994 to 2015−2019 (2000−2005 as the referent period). The dots and shaded areas denote DALY rates or rate ratios and their corresponding 95% Cis.

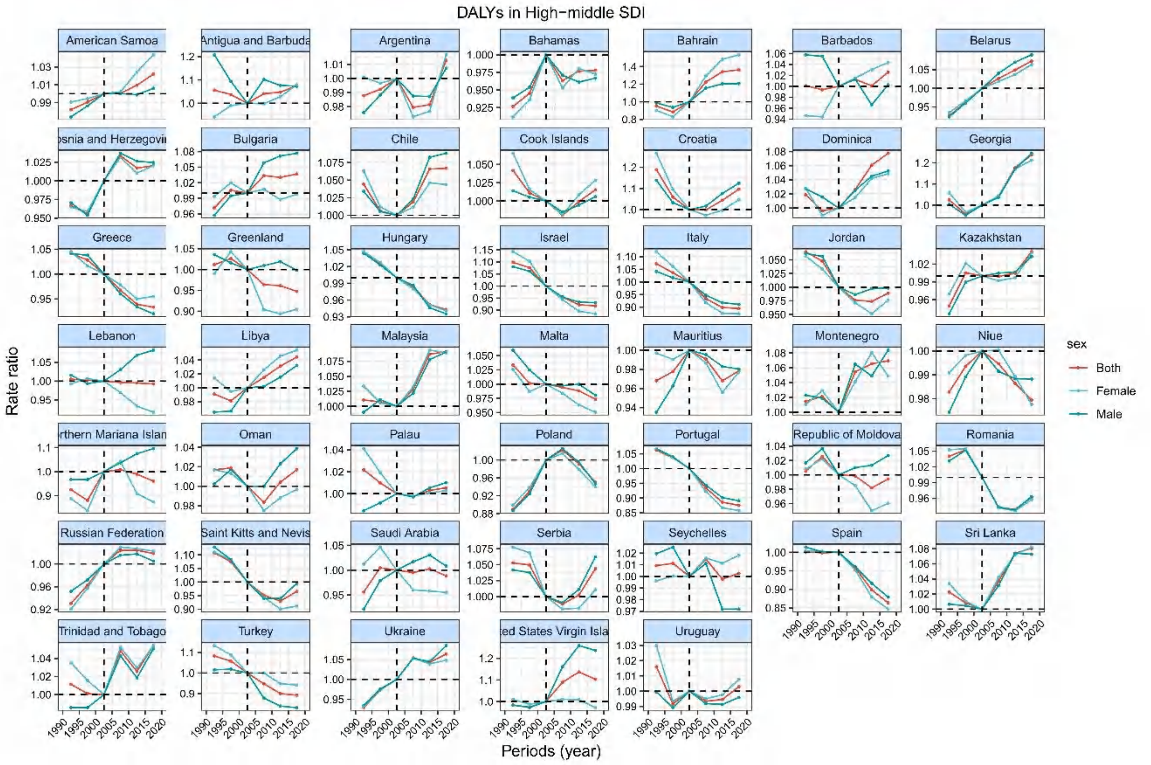

Figure S28. Period effects on atrial fibrillation and flutter DALY in middle-SDI countries Period effects are shown by the relative risk of DALY (mortality rate ratio) and computed as the ratio of age-specific rates from 1990−1994 to 2015−2019 (2000−2005 as the referent period). The dots and shaded areas denote DALY rates or rate ratios and their corresponding 95% Cis.

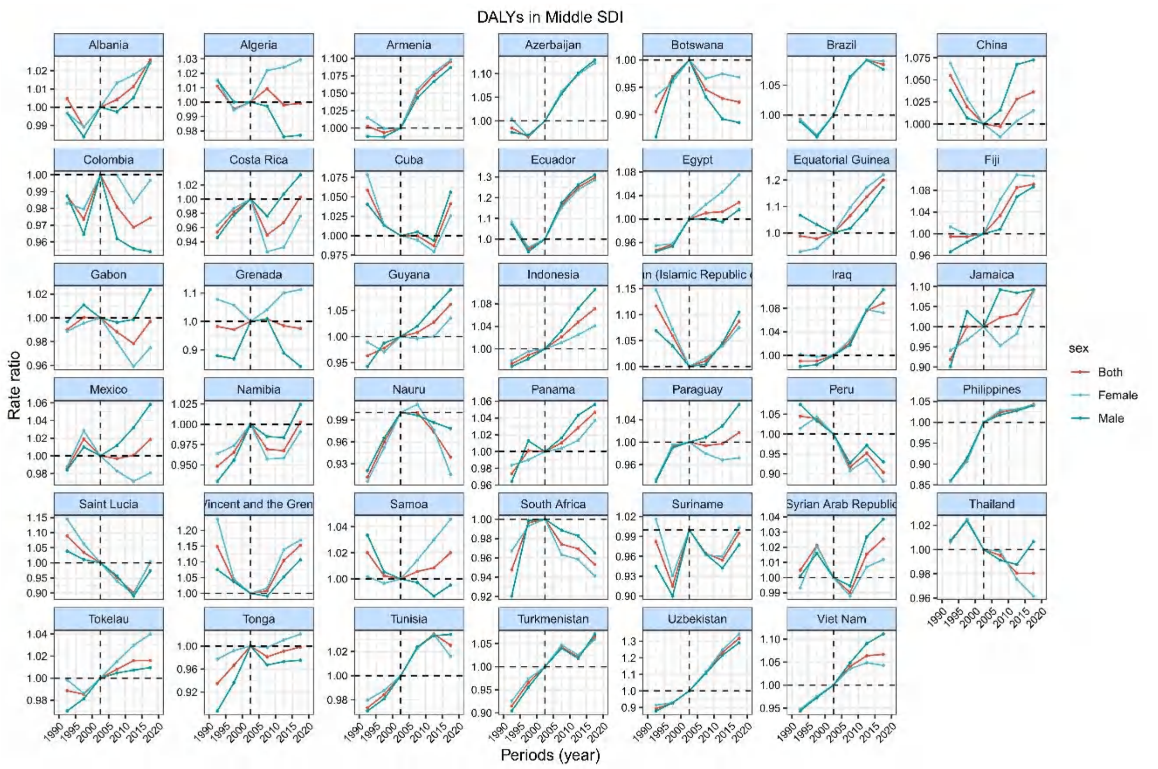

Figure S29. Period effects on atrial fibrillation and flutter DALY in low-middle SDI countries Period effects are shown by the relative risk of DALY (mortality rate ratio) and computed as the ratio of age-specific rates from 1990−1994 to 2015−2019 (2000−2005 as the referent period). The dots and shaded areas denote DALY rates or rate ratios and their corresponding 95% Cis.

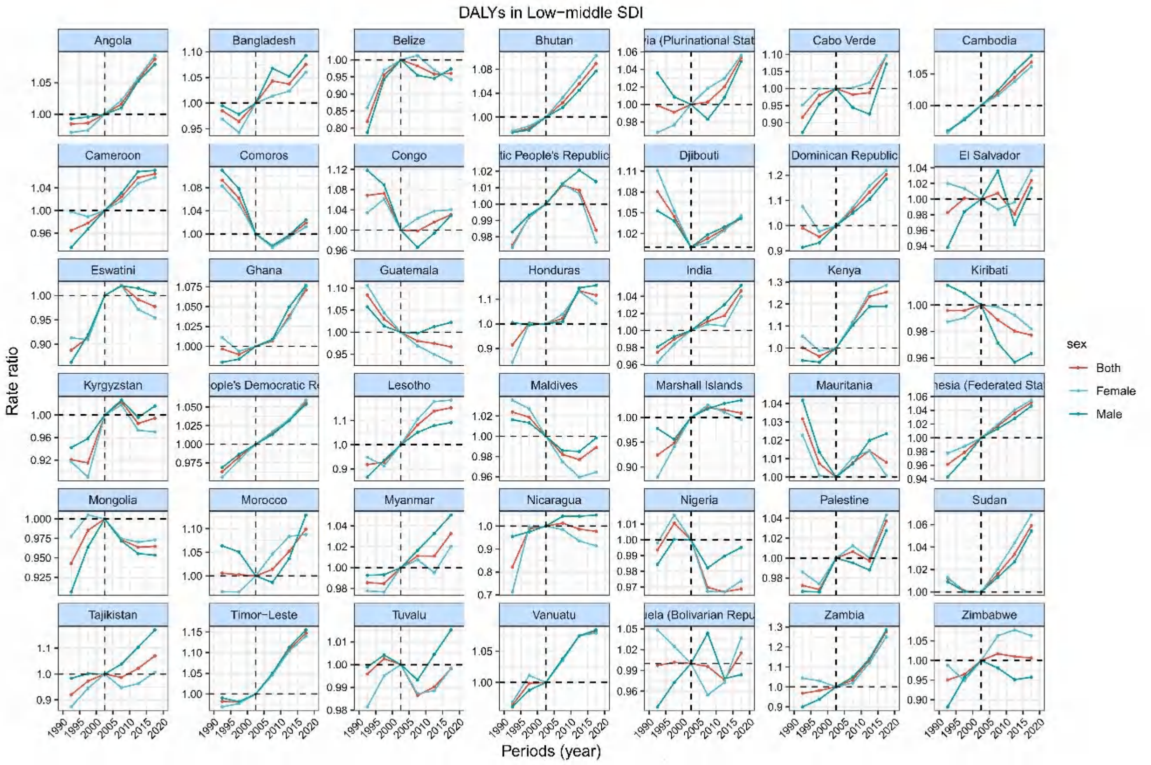

Figure S30. Period effects on atrial fibrillation and flutter DALY in low-SDI countries Period effects are shown by the relative risk of DALY (mortality rate ratio) and computed as the ratio of age-specific rates from 1990−1994 to 2015−2019 (2000−2005 as the referent period). The dots and shaded areas denote DALY rates or rate ratios and their corresponding 95% Cis.

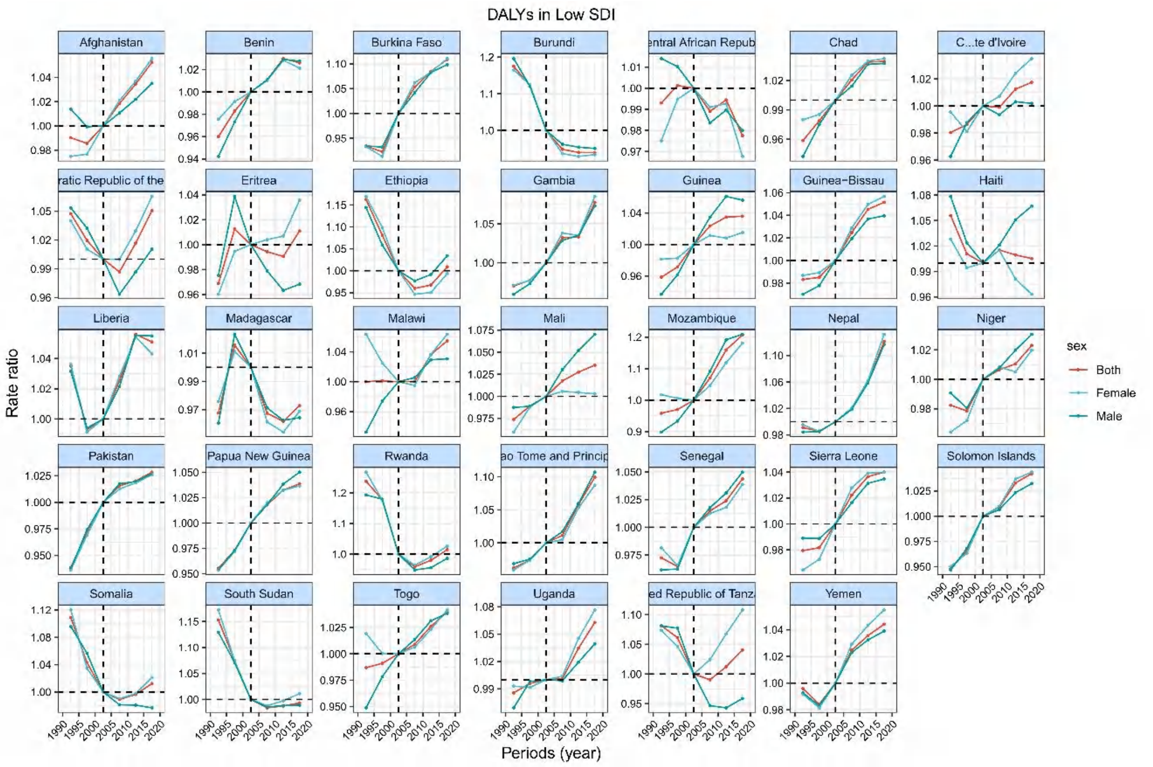

Figure S31. Cohort effects on atrial fibrillation and flutter DALY in global,1990-2019. Cohort effects are shown by the relative risk of DALY and computed as the ratio of age-specific rates from the 1895 cohort to the 2010 cohort, with the referent cohort set at 1955. The dots and shaded areas denote DALY rates or rate ratios and their corresponding 95% Cis.

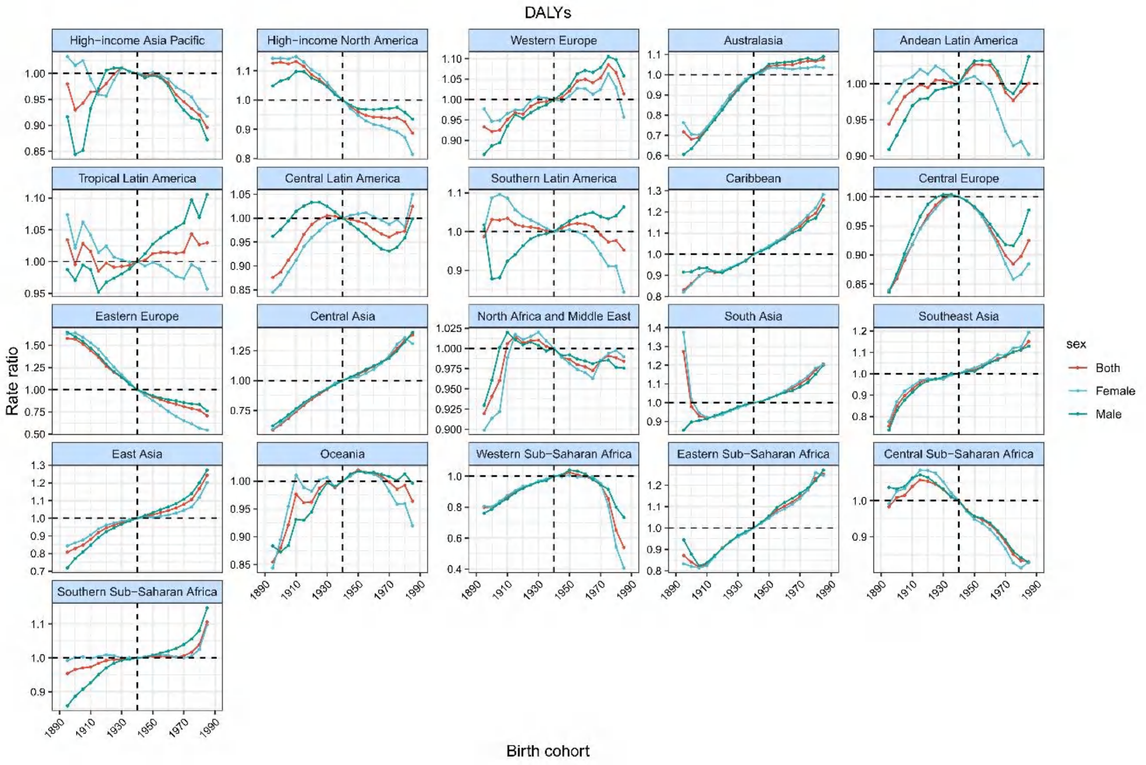

Figure S32. Cohort effects on atrial fibrillation and flutter DALY in high-SDI countries,1990-2019. Cohort effects are shown by the relative risk of DALY and computed as the ratio of age-specific rates from the 1895 cohort to the 2010 cohort, with the referent cohort set at 1955. The dots and shaded areas denote DALY rates or rate ratios and their corresponding 95% Cis.

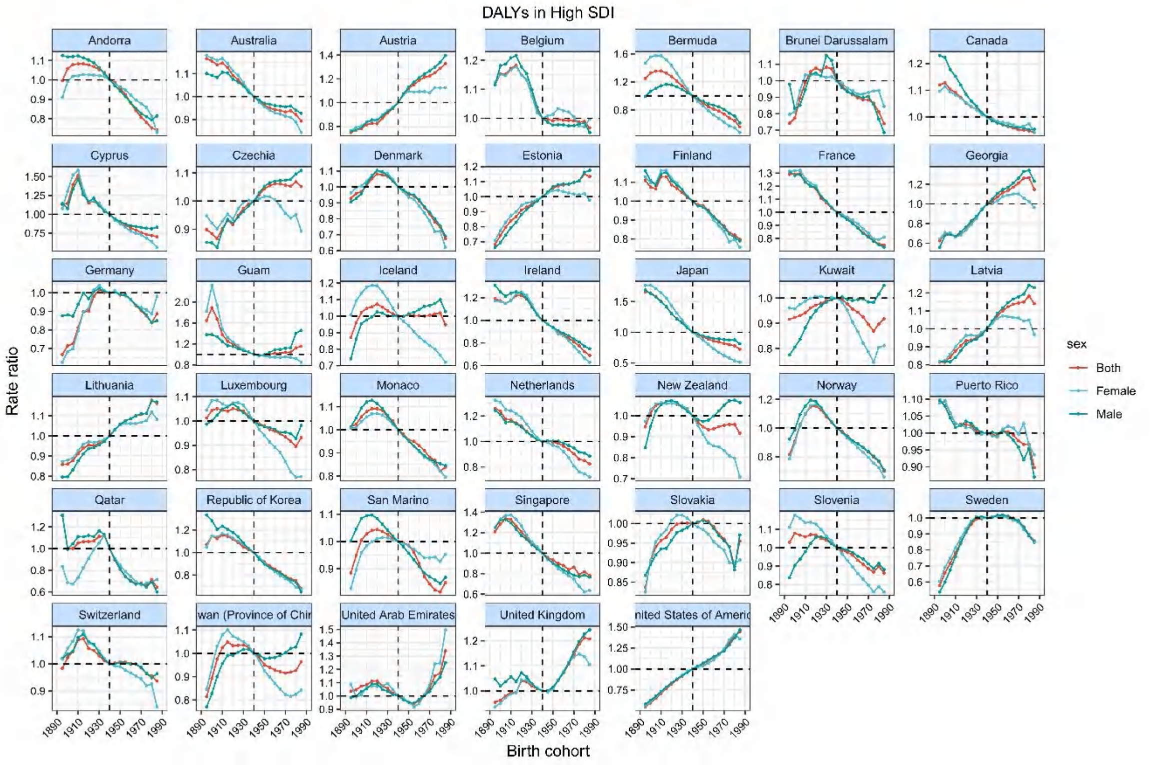

Figure S33. Cohort effects on atrial fibrillation and flutter DALY in high-middle SDI countries,1990-2019. Cohort effects are shown by the relative risk of DALY and computed as the ratio of age-specific rates from the 1895 cohort to the 2010 cohort, with the referent cohort set at 1955. The dots and shaded areas denote DALY rates or rate ratios and their corresponding 95% Cis.

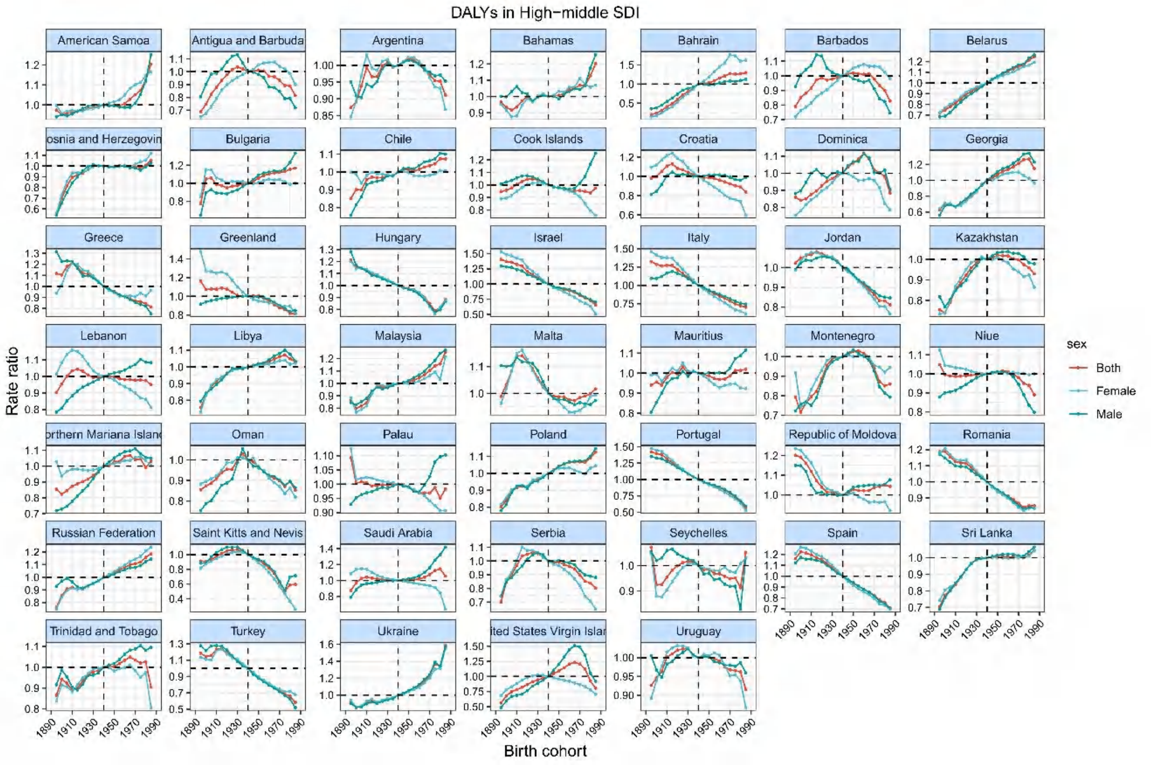

Figure S34. Cohort effects on atrial fibrillation and flutter DALY in middle-SDI countries,1990-2019. Cohort effects are shown by the relative risk of DALY and computed as the ratio of age-specific rates from the 1895 cohort to the 2010 cohort, with the referent cohort set at 1955. The dots and shaded areas denote DALY rates or rate ratios and their corresponding 95% Cis.

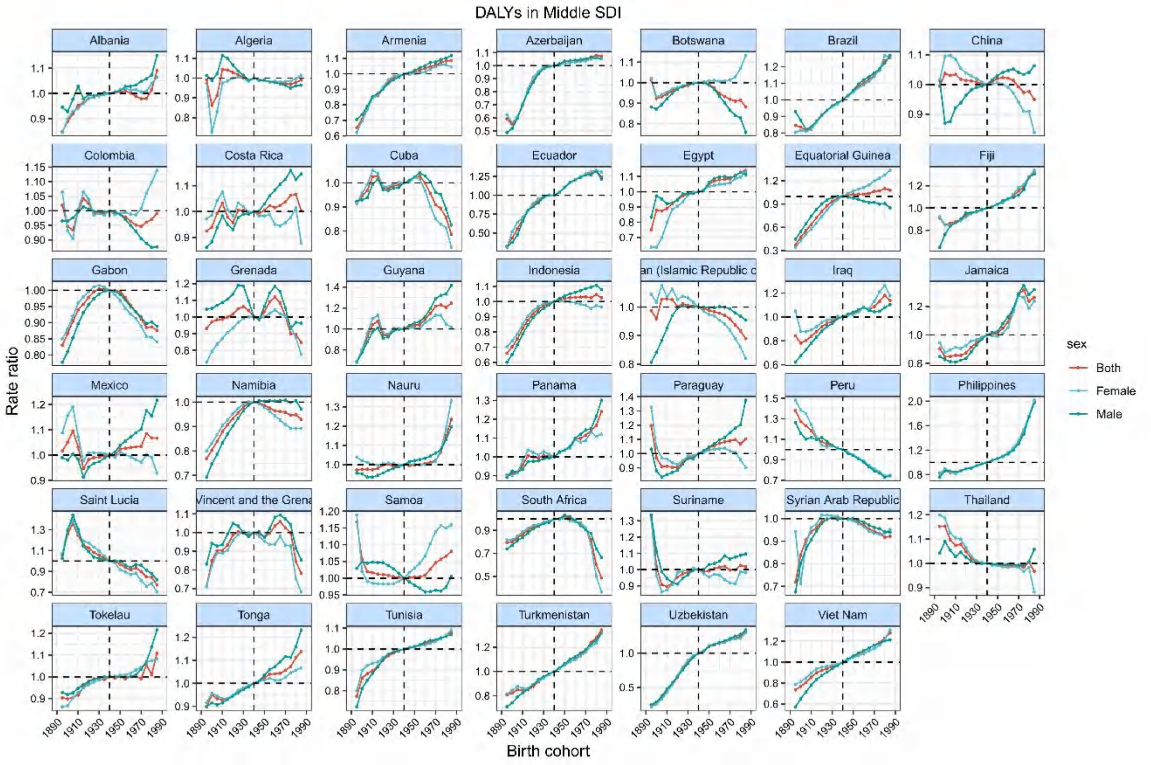

Figure S35. Cohort effects on atrial fibrillation and flutter DALY in low-middle SDI countries,1990-2019. Cohort effects are shown by the relative risk of DALY and computed as the ratio of age-specific rates from the 1895 cohort to the 2010 cohort, with the referent cohort set at 1955. The dots and shaded areas denote DALY rates or rate ratios and their corresponding 95% CIs.

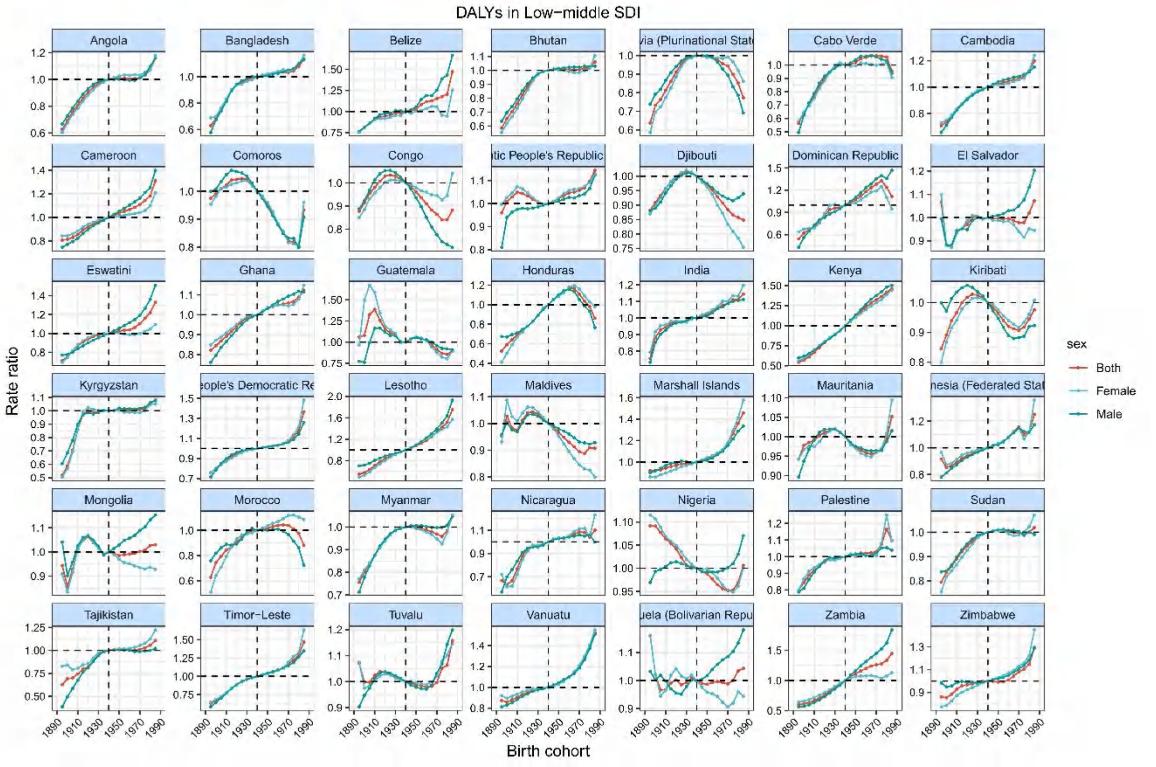

Figure S36. Cohort effects on atrial fibrillation and flutter DALY in low-SDI countries,1990-2019. Cohort effects are shown by the relative risk of DALY and computed as the ratio of age-specific rates from the 1895 cohort to the 2010 cohort, with the referent cohort set at 1955. The dots and shaded areas denote DALY rates or rate ratios and their corresponding 95% CIs.

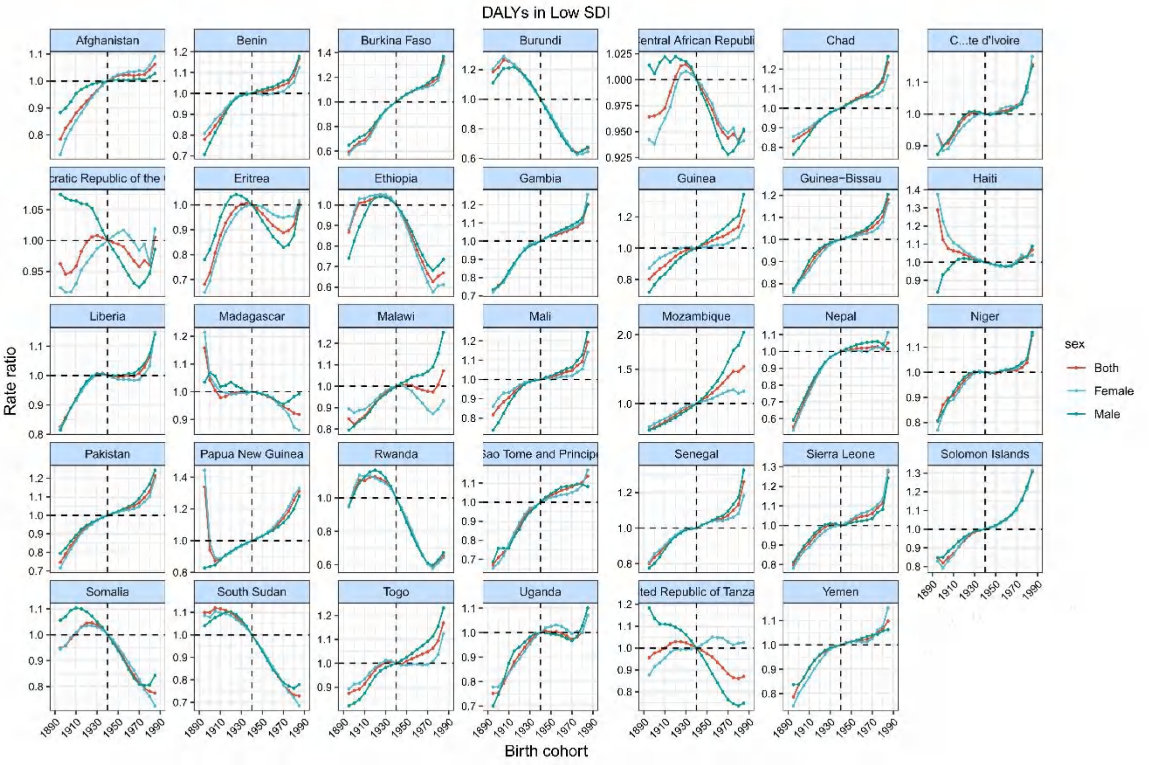

Figure S37.DALY rates of atrial fibrillation and flutter across different age groups by periods in global,1990-2019.

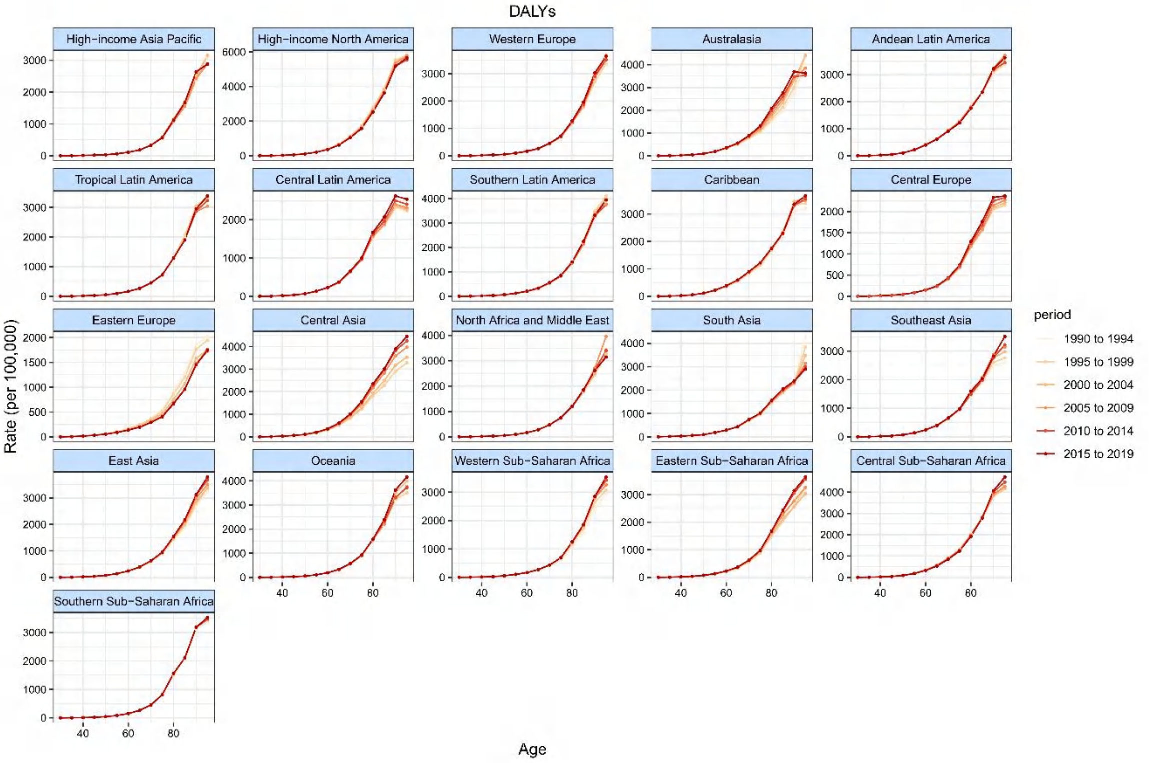

Figure S38. DALY rates of atrial fibrillation and flutter across different age

groups by periods in high-SDI countries, 1990-2019.

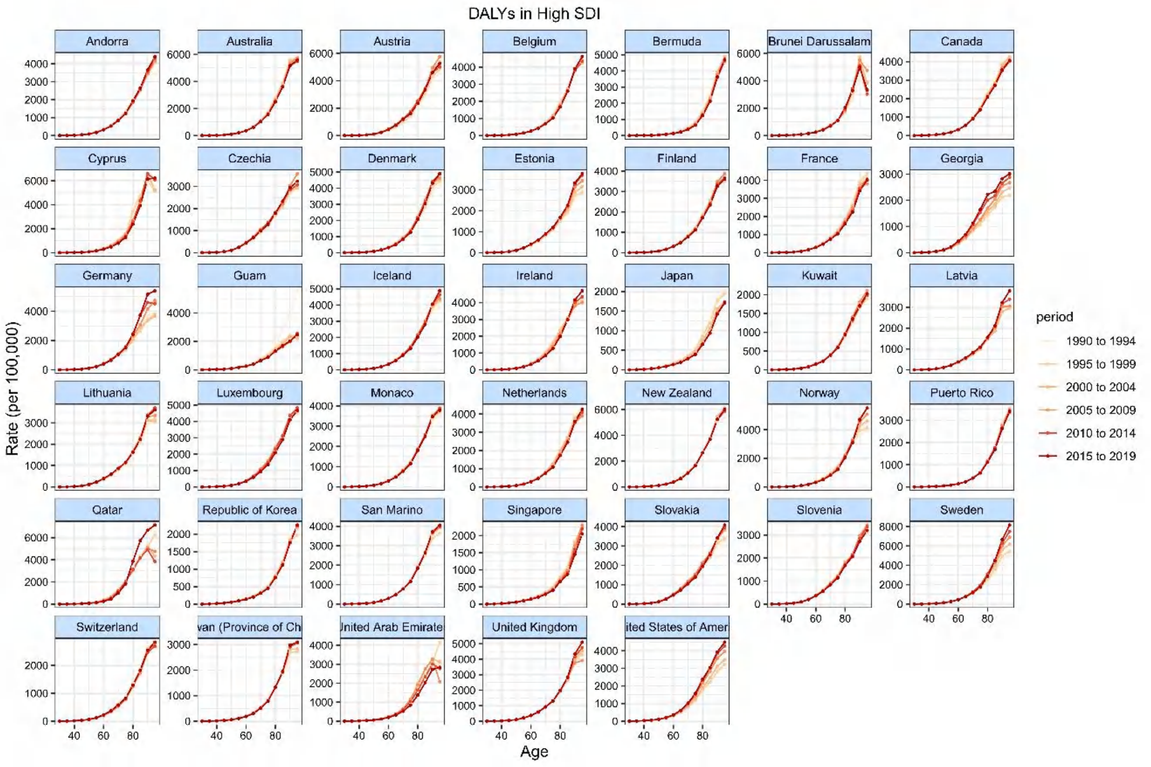

Figure S39. DALY rates of atrial fibrillation and flutter across different age groups by periods in high-middle-SDI countries, 1990-2019.

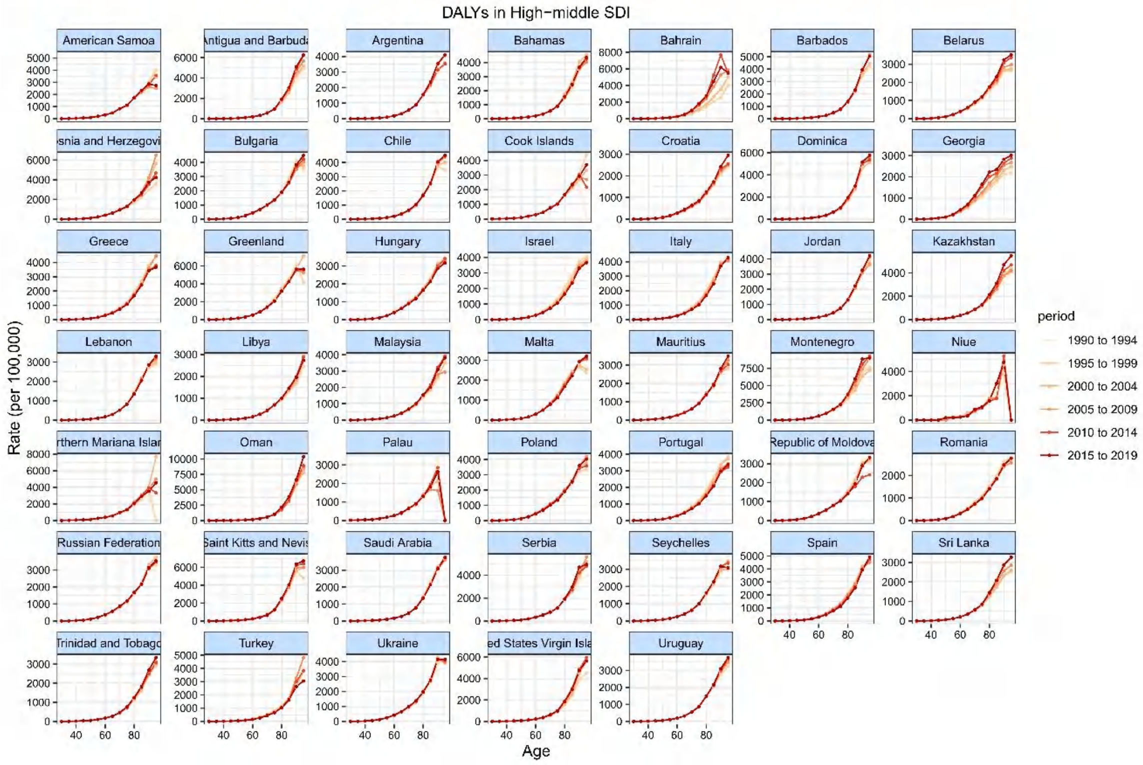

Figure S40. DALY rates of atrial fibrillation and flutter across different age groups by periods in middle-SDI countries, 1990-2019.

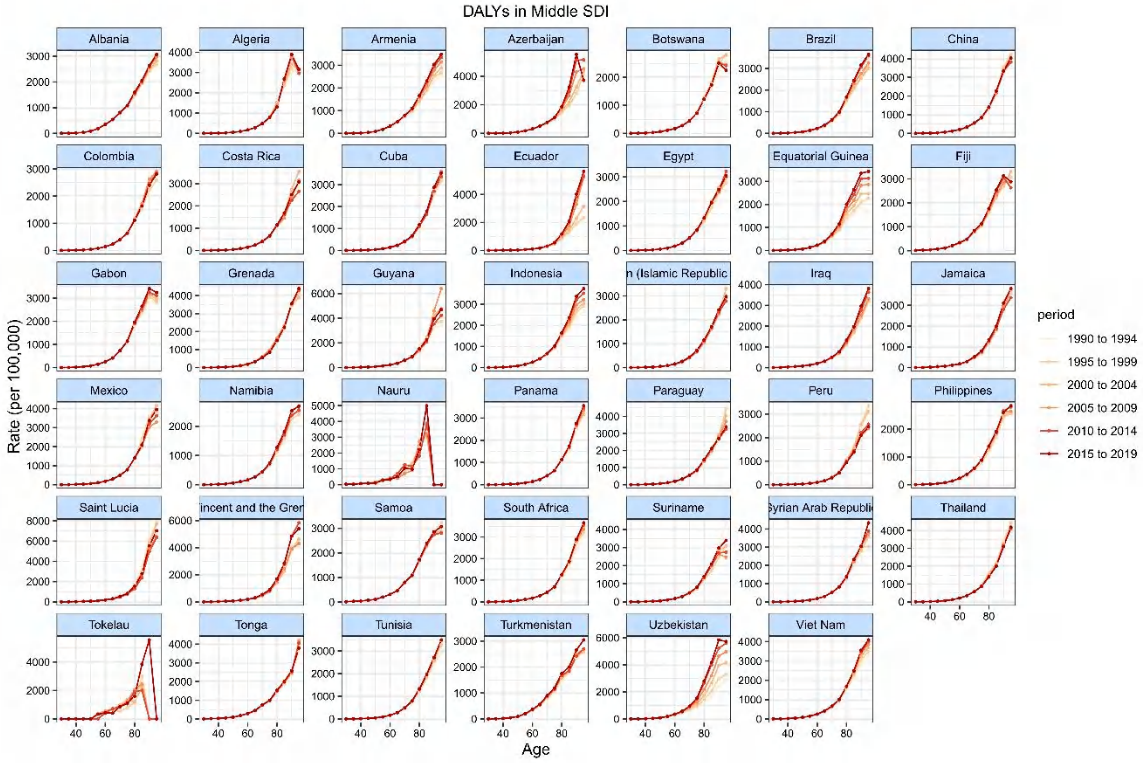

Figure S41. DALY rates of atrial fibrillation and flutter across different age groups by periods in low-middle-SDI countries, 1990-2019.

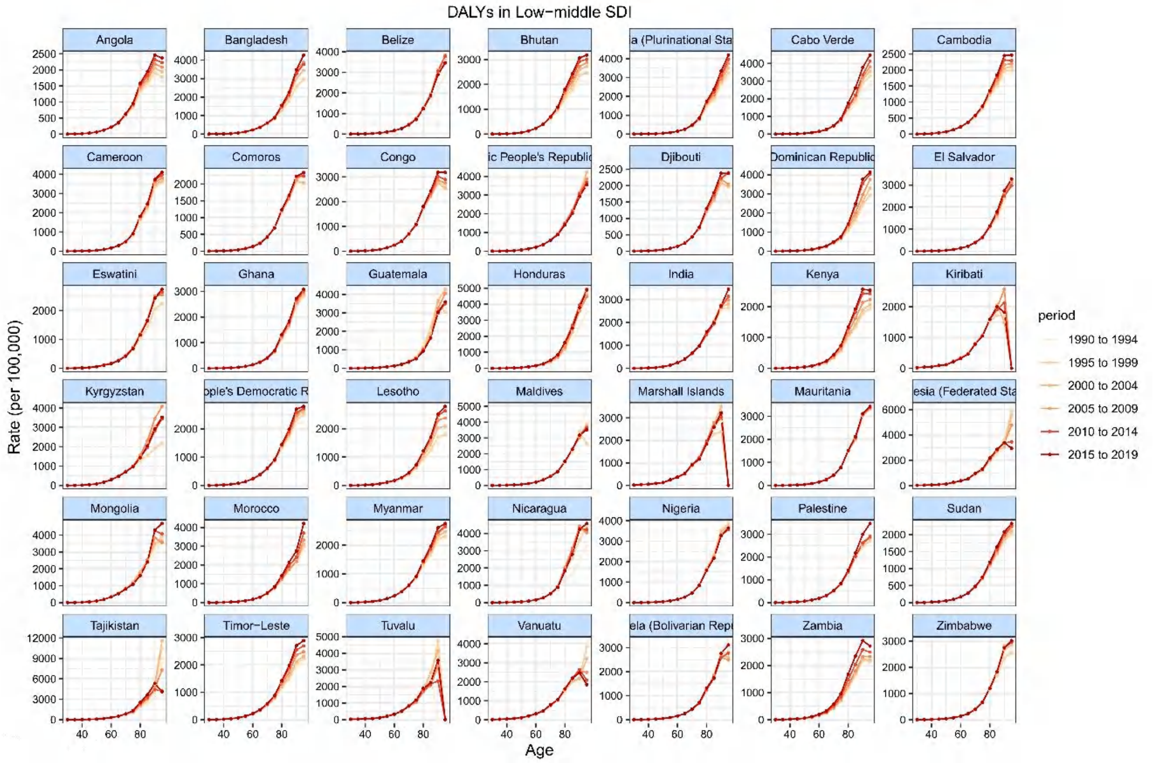

Figure S42. DALY rates of atrial fibrillation and flutter across different age groups by periods in low-SDI countries, 1990-2019.

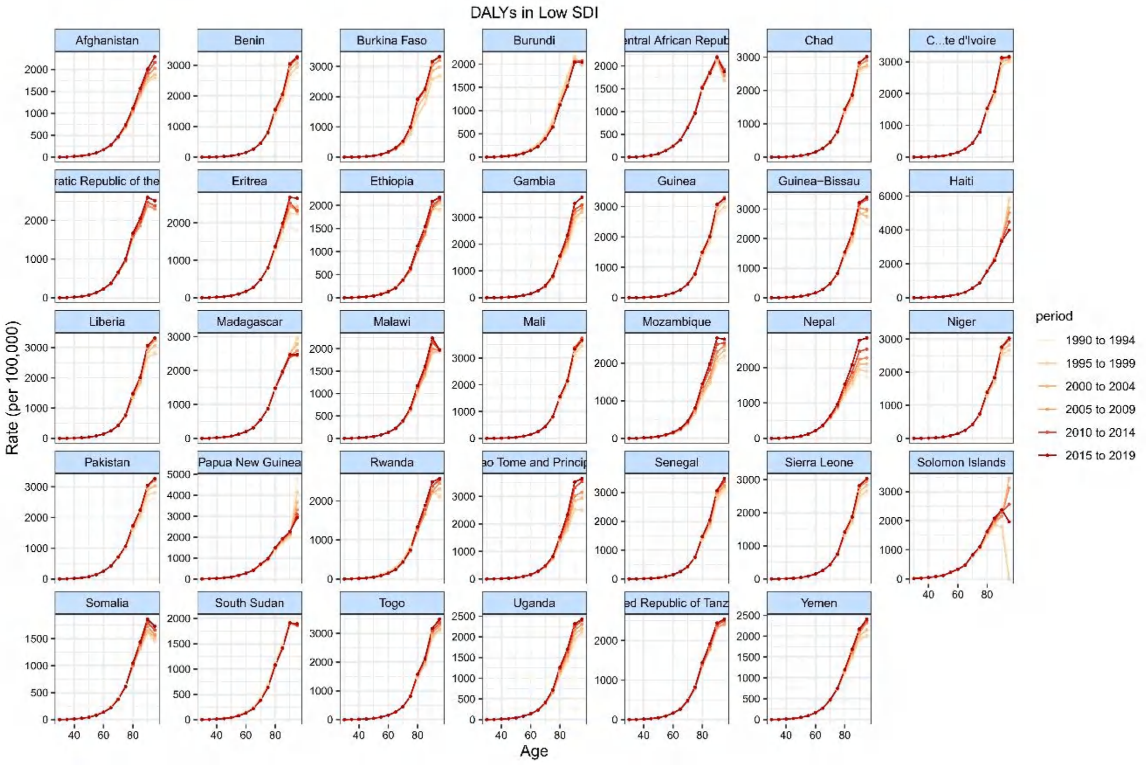

Figure S43.DALY rates of atrial fibrillation and flutter across different birth cohorts by periods in global,1990-2019.

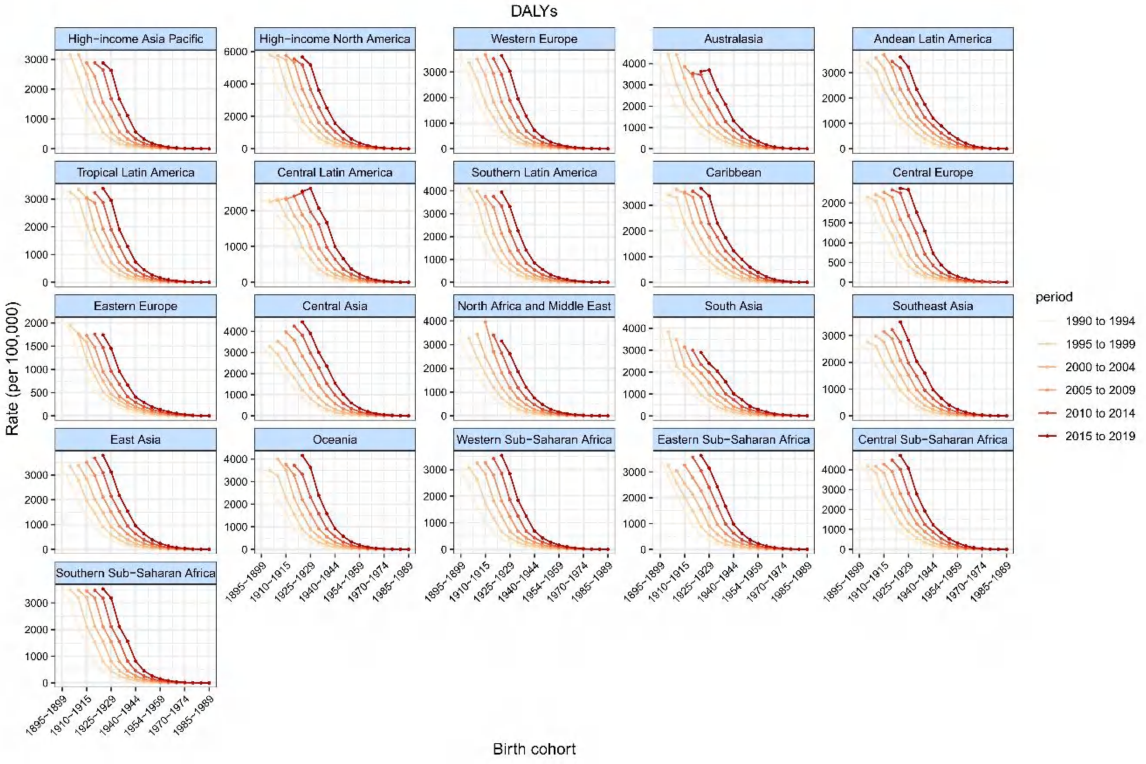

Figure S44. DALY rates of atrial fibrillation and flutter across different birth

cohorts by periods in high-SDI countries, 1990-2019.

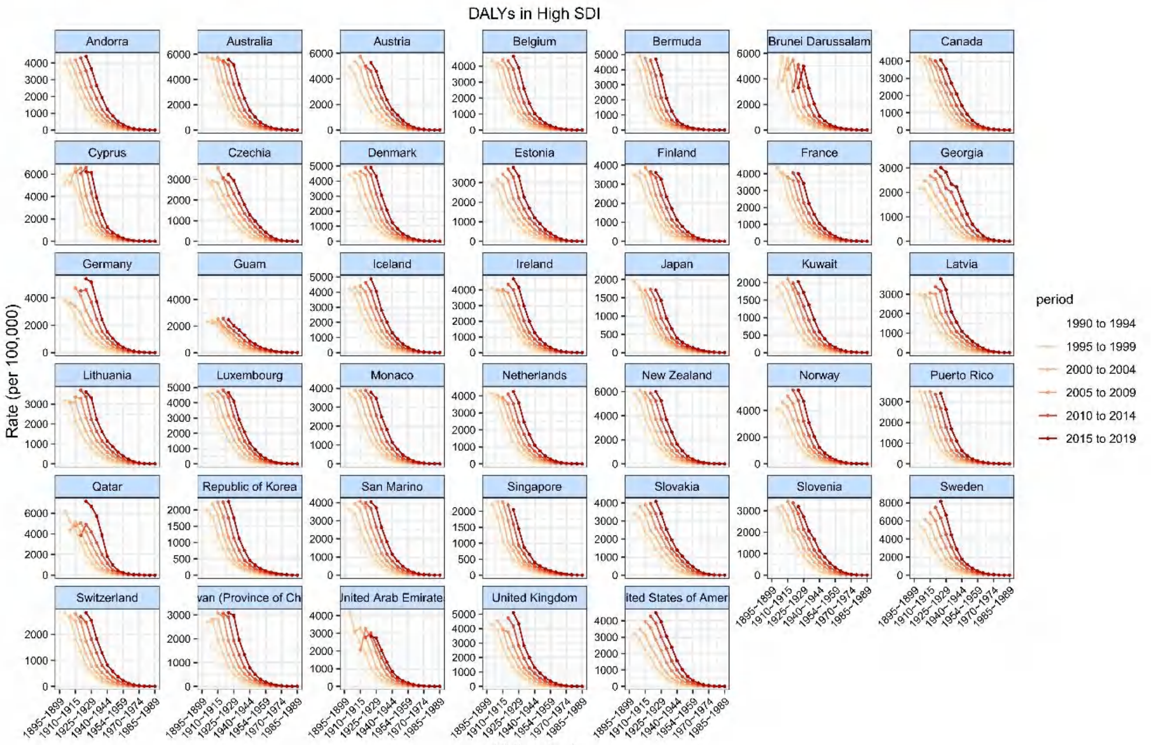

Figure S45. DALY rates of atrial fibrillation and flutter across different birth cohorts by periods in high-middle-SDI countries, 1990-2019.

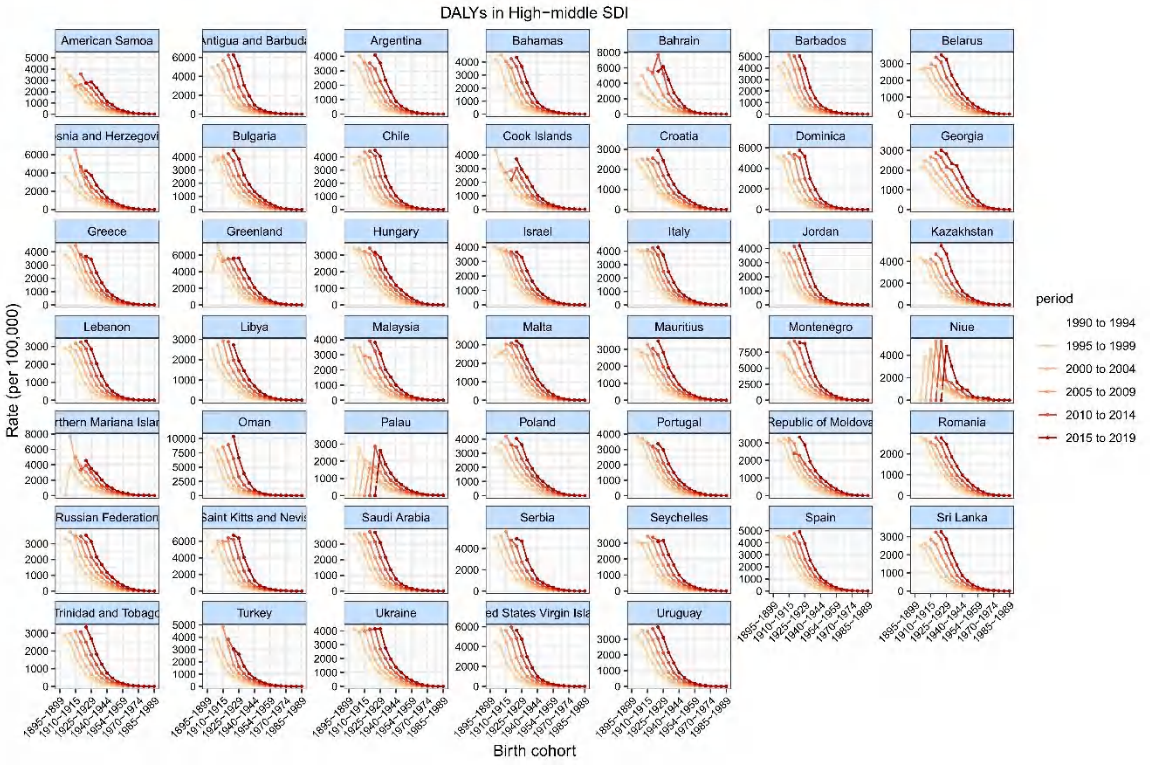

Figure S46. DALY rates of atrial fibrillation and flutter across different birth cohorts by periods in middle-SDI countries, 1990-2019.

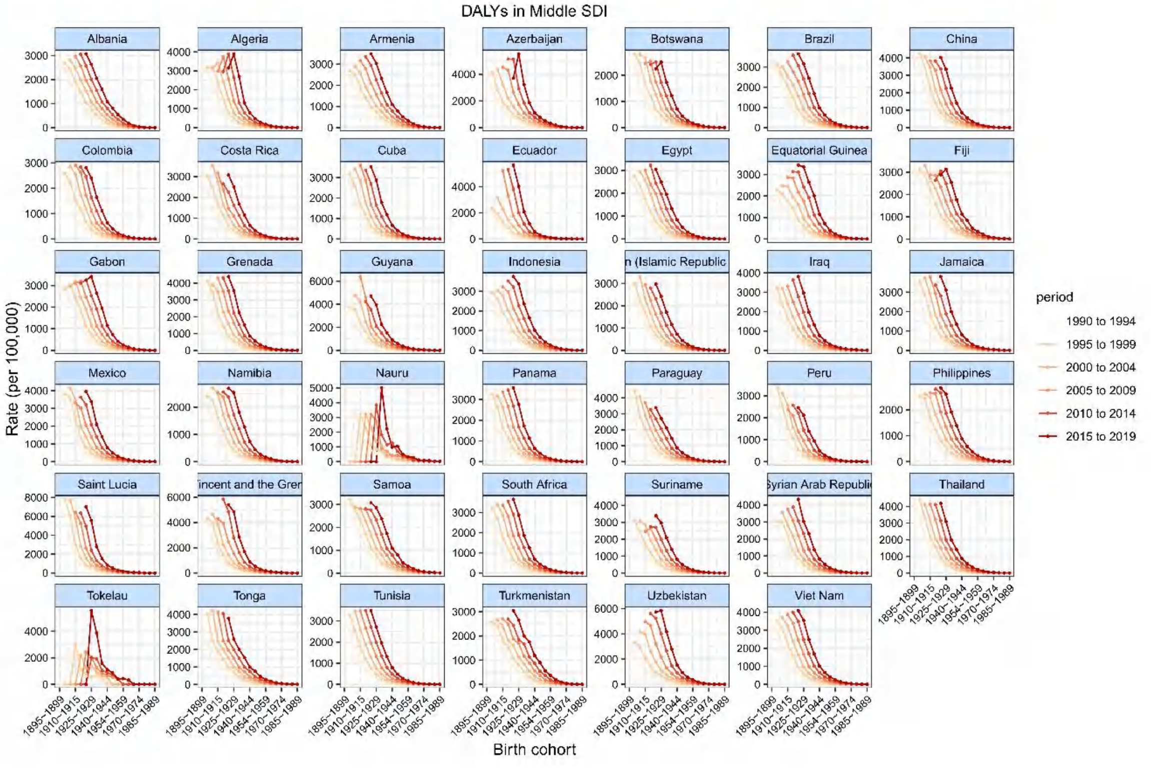

Figure S47. DALY rates of atrial fibrillation and flutter across different birth cohorts by periods in low-middle-SDI countries, 1990-2019.

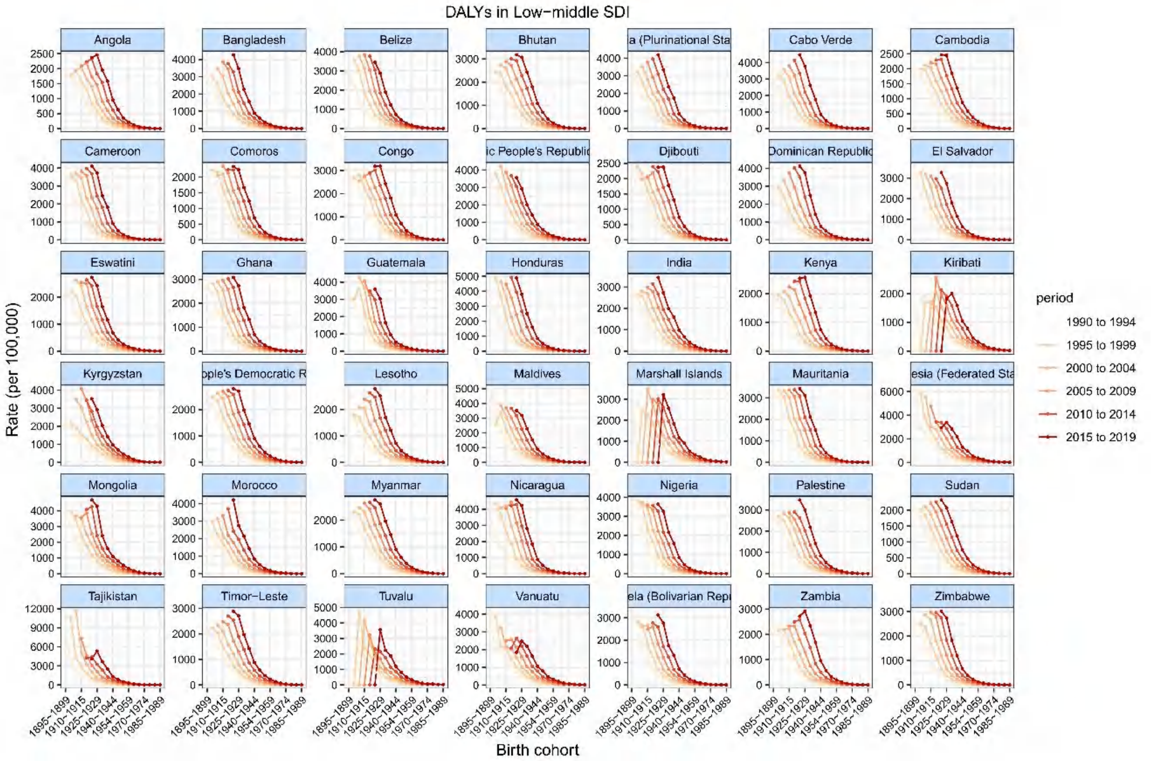

Figure S48. DALY rates of atrial fibrillation and flutter across different birth cohorts by periods in low-SDI countries, 1990-2019.

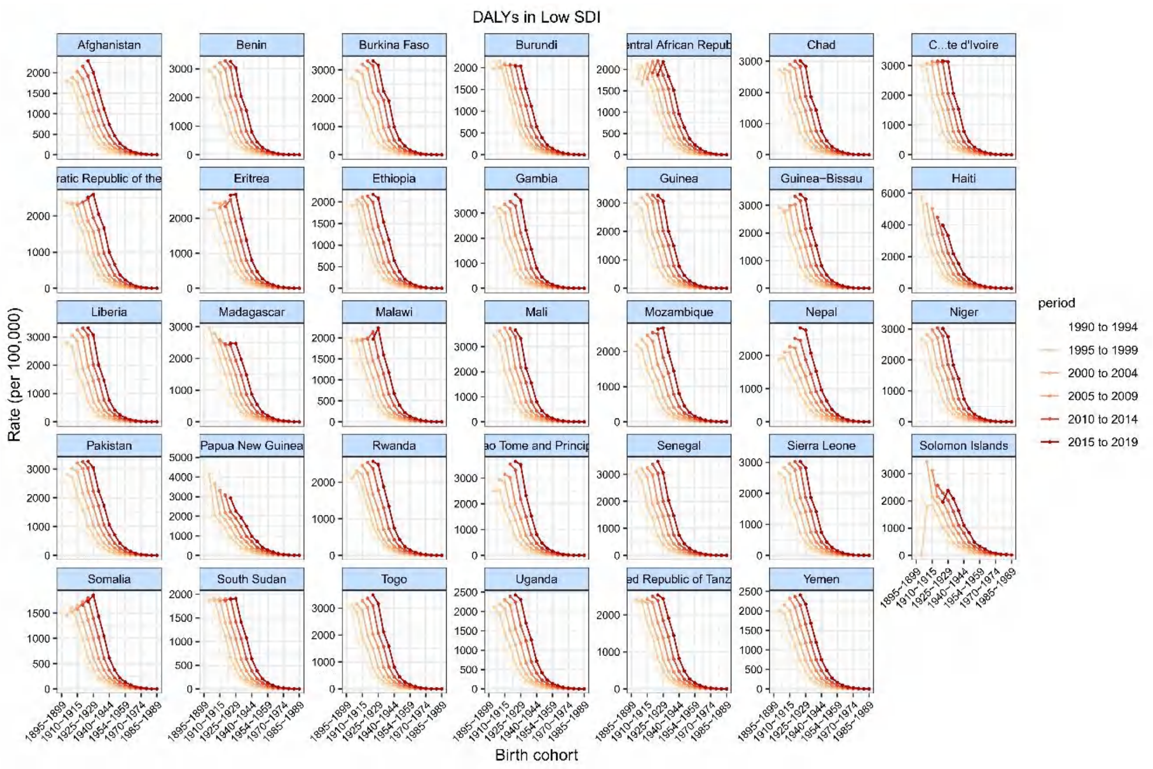

Figure S49.DALY rates of atrial fibrillation and flutter across different age groups by birth cohorts in global,1990-2019.

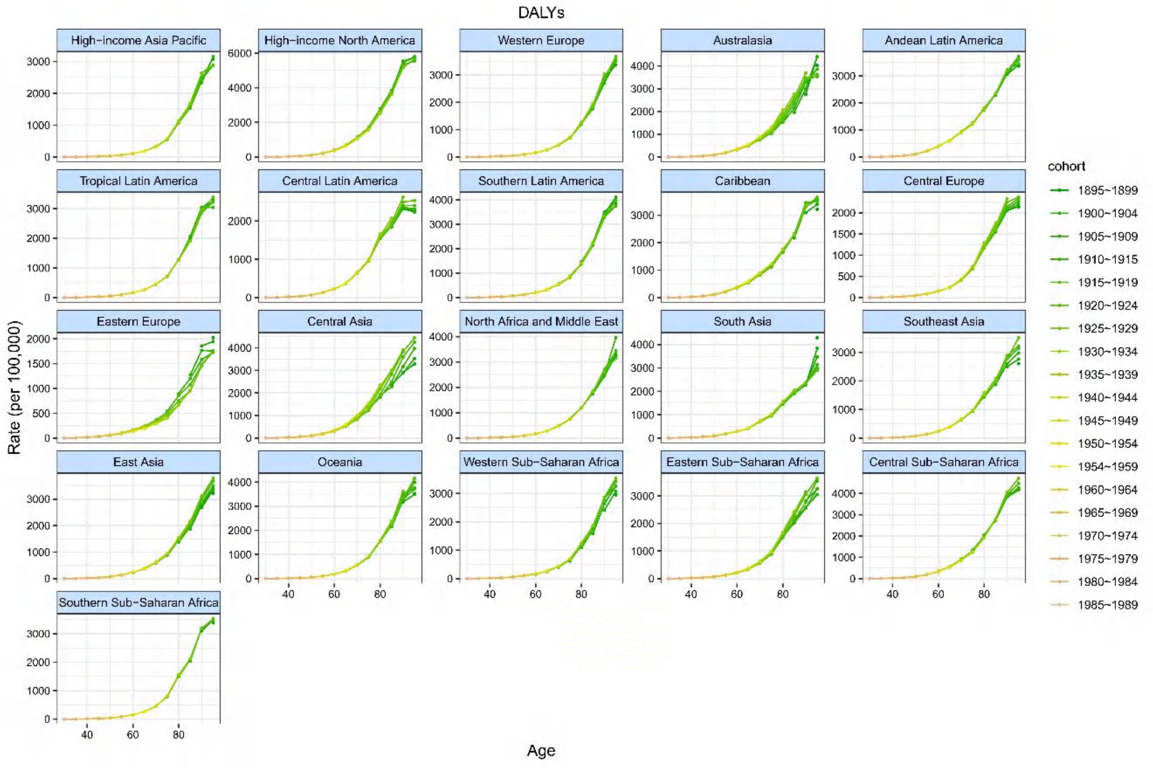

Figure S50. DALY rates of atrial fibrillation and flutter across different age groups by birth cohorts in high-SDI countries, 1990-2019.

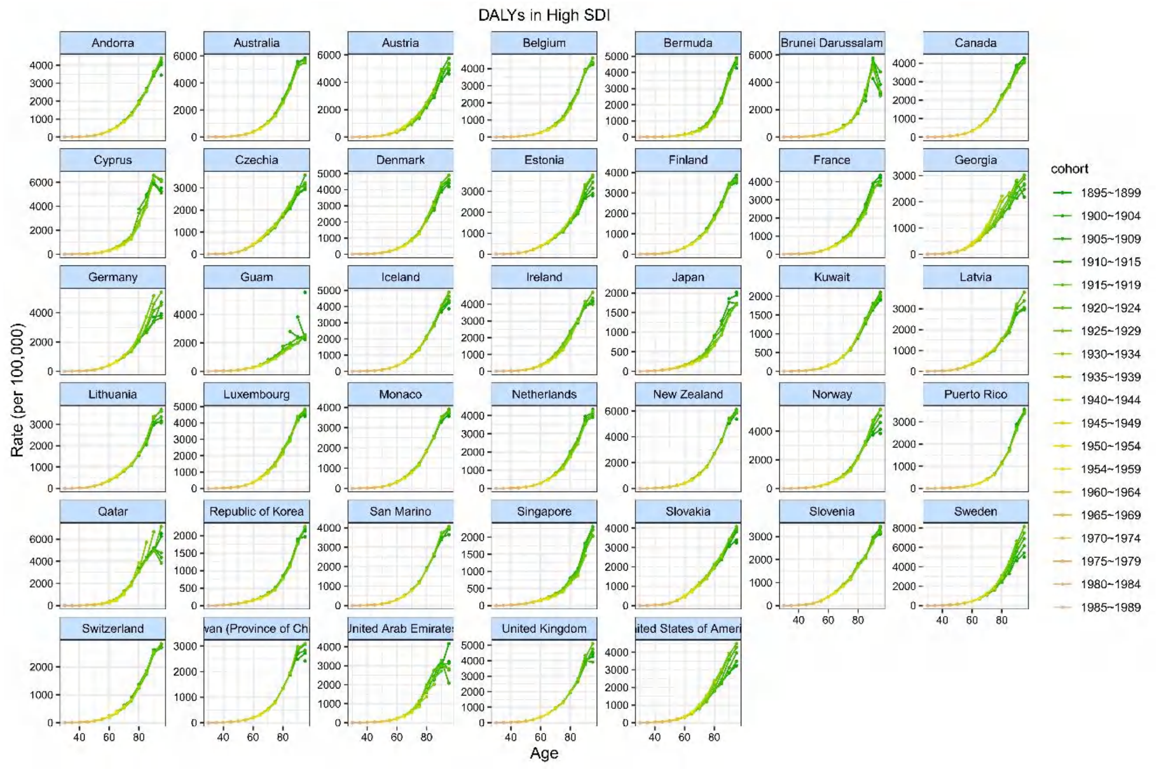

Figure S51. DALY rates of atrial fibrillation and flutter across different age groups by birth cohorts in high-middle-SDI countries, 1990-2019.

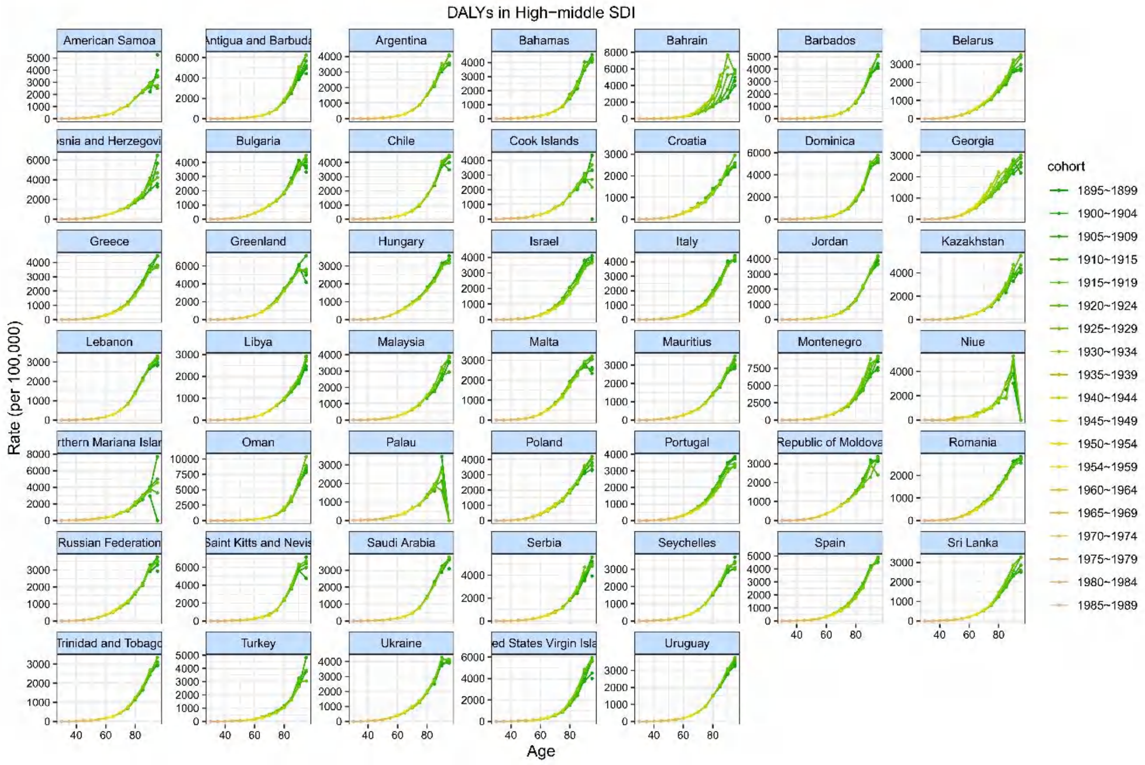

Figure S52. DALY rates of atrial fibrillation and flutter across different age groups by birth cohorts in middle-SDI countries, 1990-2019.

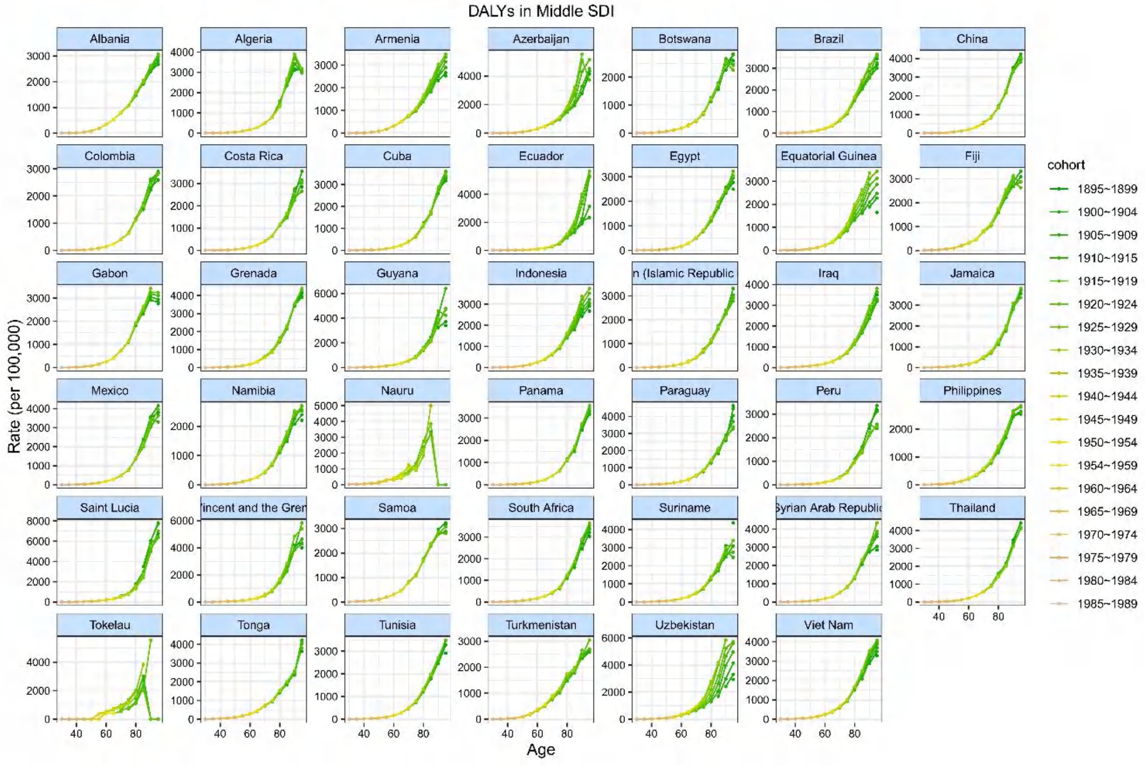

Figure S53. DALY rates of atrial fibrillation and flutter across different age groups by birth cohorts in low-middle-SDI countries, 1990-2019.

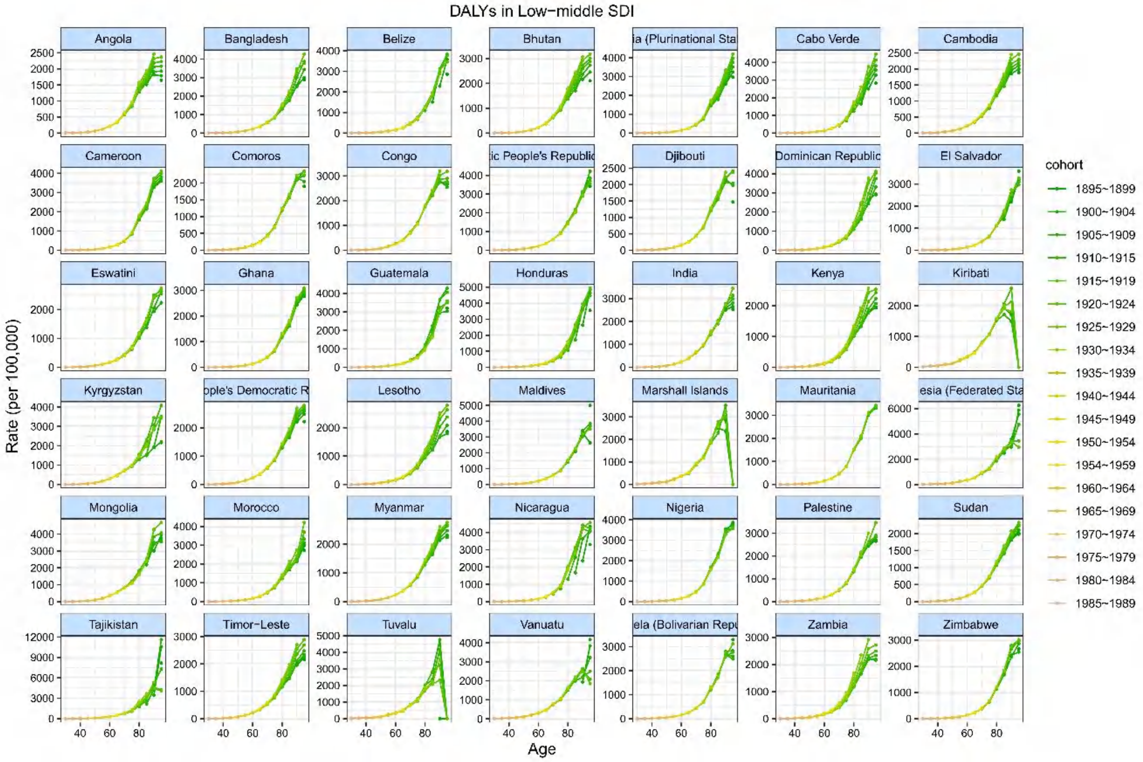

Figure S54. DALY rates of atrial fibrillation and flutter across different age groups by birth cohorts in low-SDI countries, 1990-2019.

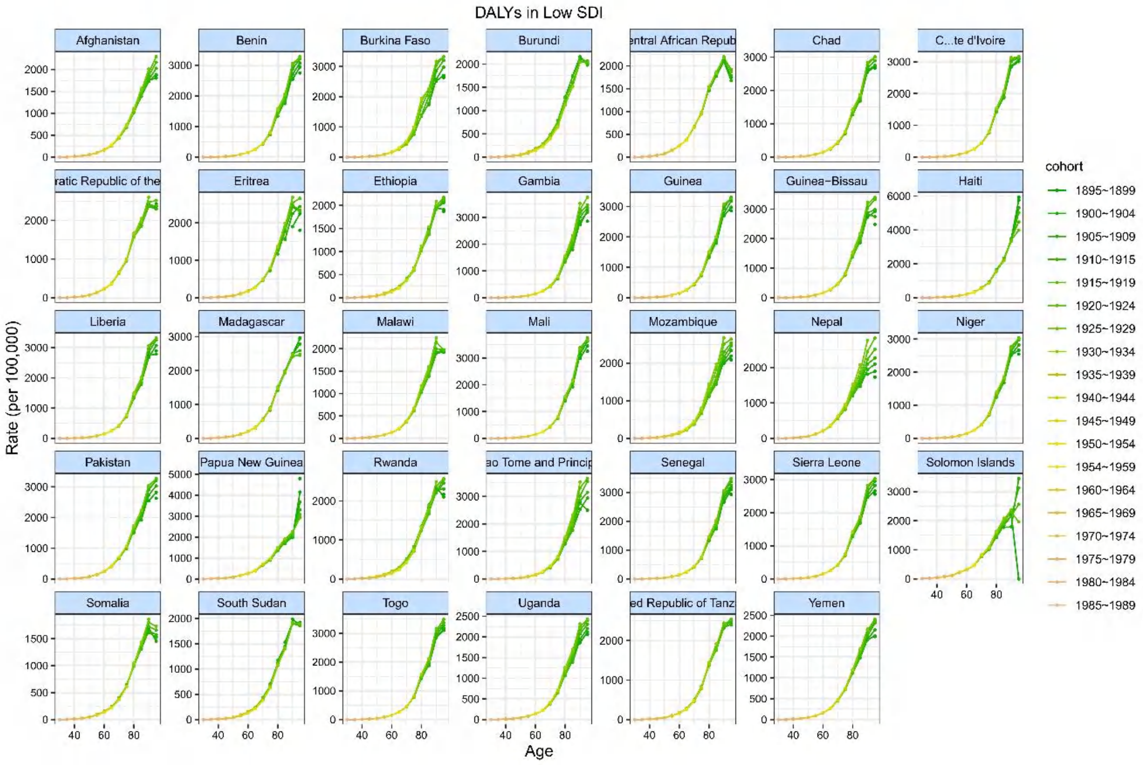
DALYs in Low SDI
Afghanistan
Benin
Burkina Faso
Burundi
entral African Repub
Chad
C...te d'Ivoire
ratic Republic of the
Eritrea
Ethiopia
Gambia
Guinea
Guinea-Bissau
Haiti
Liberia
Madagascar
Malawi
Mali
Mozambique
Nepal
Niger
Pakistan
Papua New Guinea
Rwanda
ao Tome and Princip
Senegal
Sierra Leone
Solomon Islands
Somalia
South Sudan
Togo
Uganda
ed Republic of Tanz
Yemen
Rate (per 100,000)
Age
cohort
1895~1899
1900~1904
1905~1909
1910~1915
1915~1919
1920~1924
1925~1929
1930~1934
1935~1939
1940~1944
1945~1949
1950~1954
1954~1959
1960~1964
1965~1969
1970~1974
1975~1979
1980~1984
1985~1989
